# Supplementary material for: Targeted protein degradation reveals a direct role of SPT6 in RNAPII elongation and termination
Source: Mol Cell. 2021 Aug 5;81(15):3110–3127.e14. doi: 10.1016/j.molcel.2021.06.016 (PMC8354102; doi:10.1016/j.molcel.2021.06.016)
Supplement: Document S1. Figures S1–S7 [file mmc1.pdf]

Molecular Cell, Volume *81*

Supplemental information

Targeted protein degradation reveals a direct role of SPT6 in RNAPII elongation and termination

Ashwin Narain, Pranjali Bhandare, Bikash Adhikari, Simone Backes, Martin Eilers, Lars Dölken, Andreas Schlosser, Florian Erhard, Apoorva Baluapuri, and Elmar Wolf

Figure S1

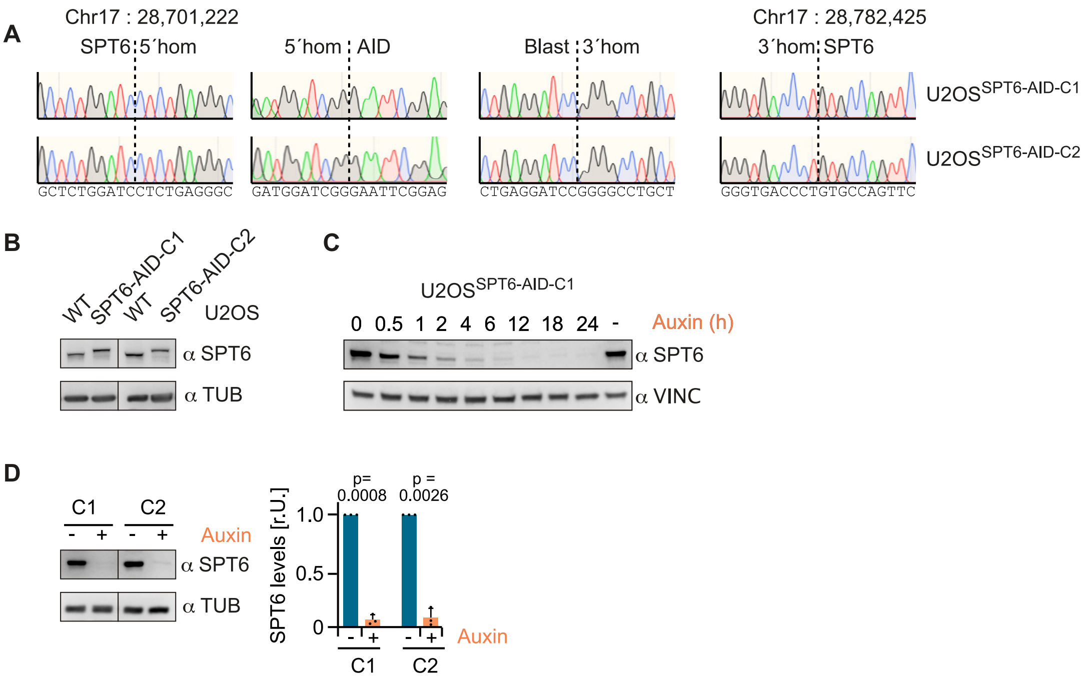

U2OS cells expressing SPT6 with C-terminal auxin-inducible degron. Related to Figure 1. (A) Sanger sequencing electropherogram of the genomic locus of U2OS$^{SPT6\text{-}AID\text{-}C1}$ and U2OS$^{SPT6\text{-}AID\text{-}C2}$ clones. 5'hom, 5' homology arms; 3'hom, 3' homology arms; Blast, blasticidin resistance gene. Genomic locations are indicated. **(B)** Immunoblot of SPT6 in wildtype (WT) U2OS, U2OS$^{SPT6\text{-}AID\text{-}C1}$ and U2OS$^{SPT6\text{-}AID\text{-}C2}$ cells. Both clones were analyzed on separate membranes. TUB, tubulin loading control. **(C)** Immunoblot of SPT6 in U2OS$^{SPT6\text{-}AID\text{-}C1}$ cells treated with auxin for the indicated times. VINC, vinculin loading control. **(D)** Immunoblot (left) of SPT6 in U2OS$^{SPT6\text{-}AID\text{-}C1}$ and U2OS$^{SPT6\text{-}AID\text{-}C2}$ cells treated with auxin (4 h). Both clones were analyzed on separate membranes. TUB, tubulin loading control. Quantification (right) of biological replicates. Values are mean ± SD, n=3. P-values were calculated with a two-tailed unpaired *t* test assuming equal variance.

Figure S2

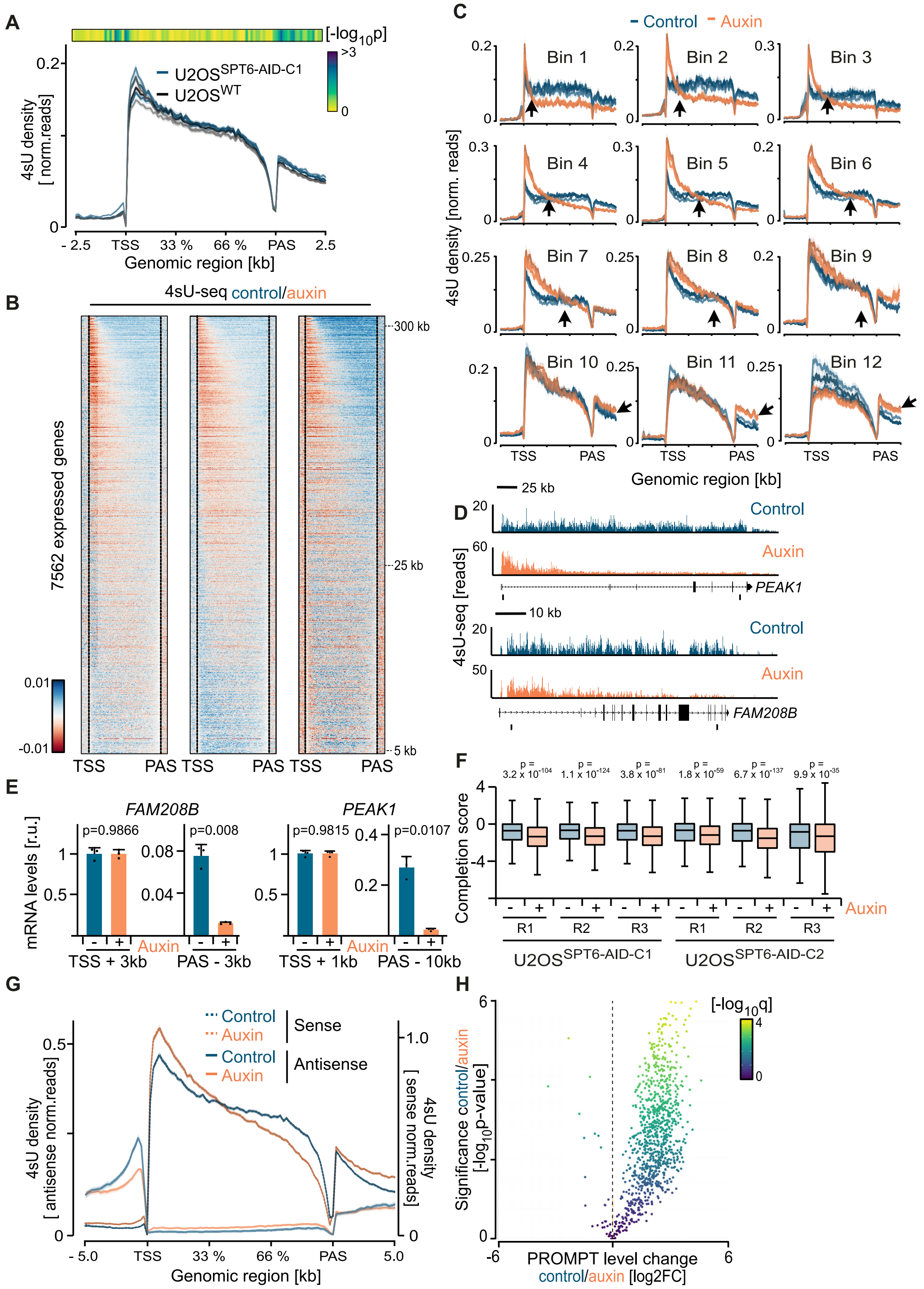
A
[-log10p]
>3
0
U2OS SPT6-AID-C1
U2OS WT
4sU density [norm.reads]
Genomic region [kb]
B
4sU-seq control/auxin
7562 expressed genes
300 kb
25 kb
5 kb
TSS
PAS
C
Control
Auxin
Bin 1
Bin 2
Bin 3
Bin 4
Bin 5
Bin 6
Bin 7
Bin 8
Bin 9
Bin 10
Bin 11
Bin 12
4sU density [norm. reads]
Genomic region [kb]
D
25 kb
10 kb
4sU-seq [reads]
PEAK1
FAM208B
E
FAM208B
PEAK1
p=0.9866
p=0.008
p=0.9815
p=0.0107
mRNA levels [r.u.]
TSS + 3kb
PAS - 3kb
TSS + 1kb
PAS - 10kb
F
Completion score
U2OS SPT6-AID-C1
U2OS SPT6-AID-C2
G
Sense
Antisense
4sU density [antisense norm.reads]
4sU density [sense norm.reads]
Genomic region [kb]
H
[-log10q]
Significance control/auxin [-log10p-value]
PROMPT level change control/auxin [log2FC]

Effect of SPT6 depletion is conserved across replicates. Related to Figure 2. (A) Metagene plot showing the distribution of read density over the gene body from TSS to PAS averaged over 7,562 expressed genes for U2OS^{WT} and untreated U2OS$^{SPT6\text{-}AID\text{-}C1}$ cells for three biological replicates. Shadows around curves indicate SEM values. P-values (two-sided Wilcoxon test) for the difference (auxin/control), calculated from the density values of individual genes (n=7,562) at each genomic location, are shown in a heatmap. **(B)** Relative heatmaps for three replicates showing normalized $\log_2$ fold change between control and auxin-treated U2OS$^{SPT6\text{-}AID\text{-}C1}$ cells from 4sU-seq reads in 7,562 expressed genes sorted by length and scaled to the same length. Orange indicates less reads and blue indicates more reads in control condition. **(C)** Metagene plots of the distribution of read density over the gene body from TSS to PAS, averaged over different bins of genes stratified by length. Shadows around curves indicate SEM values, and arrows indicate the crossover points between curves. Genes were sorted according to length and grouped into bins: Bin 1 contains the longest genes and bin 12 the shortest genes. **(D)** Browser tracks of one replicate from a 4sU-seq experiment for *PEAK1* and *FAM208B* in U2OS$^{SPT6\text{-}AID\text{-}C1}$ cells in the presence or absence of auxin (6 h). Primers for quantitative PCR are indicated below. **(E)** qPCR analysis of RNAPII processivity. Graphs show signals at *PEAK1* and *FAM208B* genes (primers shown in Fig S2D), relative to TSS proximal primers. Values are mean ± SD (n=3). Two-tailed unpaired *t* test assuming equal variance. **(F)** Completion scores for three biological replicates in U2OS$^{SPT6\text{-}AID\text{-}C1}$ and U2OS$^{SPT6\text{-}AID\text{-}C2}$ cells in presence (orange) or absence (blue) of auxin. Unpaired two-sided Wilcoxon test. **(G)** Strand-separated metagene plot showing the distribution of sense and antisense read densities over the gene body from TSS to PAS, averaged over 7,562 expressed genes for U2OS$^{SPT6\text{-}AID\text{-}C1}$ control cells compared to cells treated with auxin (6 h) for reads combined from three biological replicates. **(H)** Volcano plot comparing $\log_2$ fold change and the statistical significance of antisense reads in 1,052 annotated PROMPTs (Schlackow et al., 2017), calculated in control and auxin conditions. The color of individual data points indicates the corresponding PROMPT's adjusted p-value ($-\log_{10}$(qvalue)).

Figure S3

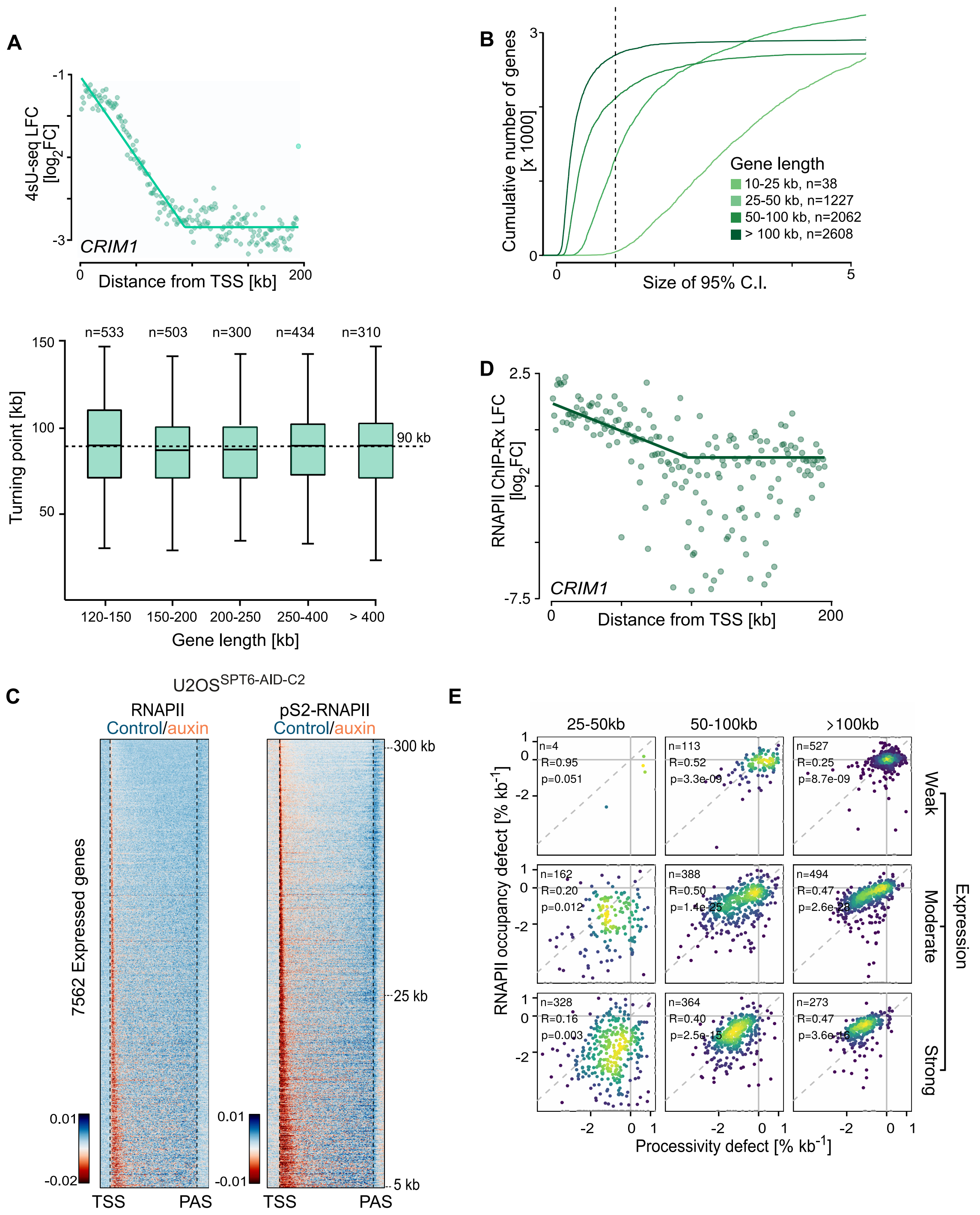

A
4sU-seq LFC [log2FC]
CRIM1
Distance from TSS [kb]
Turning point [kb]
n=533
n=503
n=300
n=434
n=310
90 kb
120-150
150-200
200-250
250-400
> 400
Gene length [kb]
B
Cumulative number of genes [x 1000]
Size of 95% C.I.
Gene length
10-25 kb, n=38
25-50 kb, n=1227
50-100 kb, n=2062
> 100 kb, n=2608
D
RNAPII ChIP-Rx LFC [log2FC]
CRIM1
Distance from TSS [kb]
C
U2OS SPT6-AID-C2
RNAPII
Control/auxin
pS2-RNAPII
Control/auxin
7562 Expressed genes
300 kb
25 kb
5 kb
TSS
PAS
E
25-50kb
50-100kb
>100kb
RNAPII occupancy defect [% kb-1]
Processivity defect [% kb-1]
Weak
Moderate
Strong
Expression

Differentiation between elongation rate defects and processivity defects. Related to Figure 3. (A) Top, local fold change (LFC) for example gene *CRIM1*. The plot shows log_2 fold changes in read counts in non-overlapping 1 kb windows for the pooled 4sU-seq replicates in U2OS$^{SPT6\text{-}AID\text{-}C1}$ cells in presence vs. absence of auxin (6 h). Bottom, distributions of turning point positions in LFC fits for 4sU signal, according to gene length. The number of genes per bin is indicated. **(B)** Cumulative distribution of size of the 95% confidence interval (CI) for the processivity defect parameter estimated with LFC regression, for four gene groups according to length. The number of genes in each length-based bin is shown. The size of the 95% CI for the rate of the exponential decline in 4sU signal along the gene body for auxin-treated samples compared to control samples (% kb^{-1}) is shown on the X-axis. **(C)** Relative heatmaps of ChIP-Rx experiments in U2OS$^{SPT6\text{-}AID\text{-}C2}$ cells with antibodies against pS2-RNAPII (right) or RNAPII (left) over 7,562 expressed genes scaled to the same length. Orange indicates less reads and blue indicates more reads in control cells. **(D)** LFC for example gene *CRIM1*. The plot shows log_2 fold change of read counts in non-overlapping 1 kb windows for a RNAPII ChIP-Rx experiment in U2OS$^{SPT6\text{-}AID\text{-}C1}$ cells in presence vs. absence of auxin (6 h). **(E)** Scatter plots correlating processivity defects with RNAPII occupancy for 2,393 genes with accurate LFC fits for the 4sU and ChIP data. Processivity defect is the rate of exponential decline in 4sU signal along the gene body for auxin-treated samples compared to control samples. Y-axis indicates the same parameter for RNAPII ChIP signal. The genes are stratified by expression level (weak, moderate, strong) and length.

Figure S4

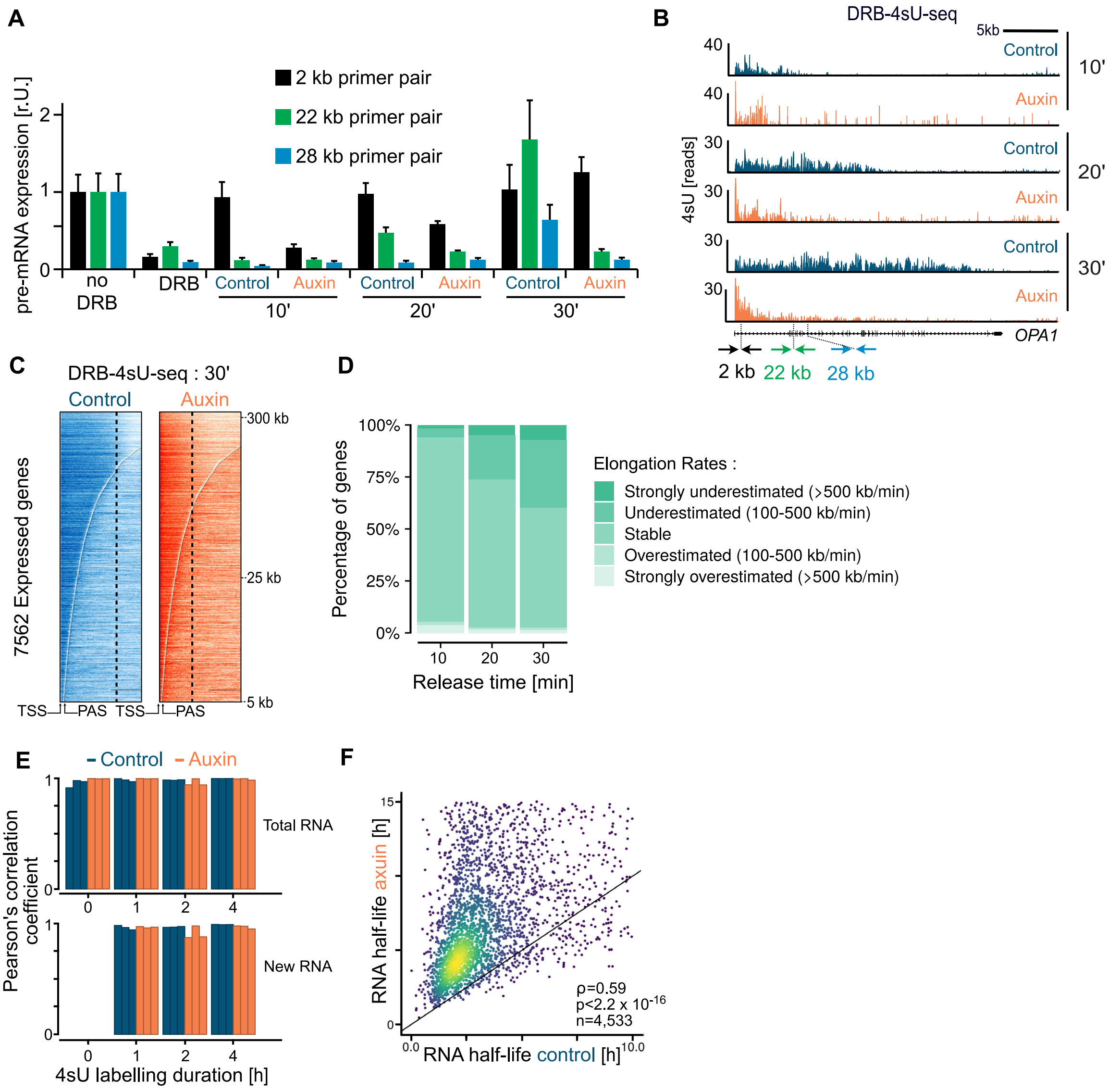

A
2 kb primer pair
22 kb primer pair
28 kb primer pair
pre-mRNA expression [r.U.]
no DRB
DRB
Control
Auxin
10'
20'
30'
B
DRB-4sU-seq
5kb
Control
Auxin
4sU [reads]
OPA1
2 kb
22 kb
28 kb
C
DRB-4sU-seq : 30'
Control
Auxin
7562 Expressed genes
300 kb
25 kb
5 kb
TSS
PAS
D
Percentage of genes
Release time [min]
Elongation Rates :
Strongly underestimated (>500 kb/min)
Underestimated (100-500 kb/min)
Stable
Overestimated (100-500 kb/min)
Strongly overestimated (>500 kb/min)
E
Control
Auxin
Pearson's correlation coefficient
Total RNA
New RNA
4sU labelling duration [h]
F
RNA half-life axuin [h]
RNA half-life control [h]
ρ=0.59
p<2.2 x 10-16
n=4,533

Acute SPT6 depletion leads to processivity loss. Related to Figure 4. (A) Quantification of 4sU-seq reads using RT-qPCR in example gene *OPA1* after DRB inhibition and release. Locations of primers are indicated as the distance from TSS, shown in panel (B). **(B)** Browser tracks showing a DRB-4sU-seq experiment at *OPA1* gene in U2OS$^{SPT6\text{-}AID\text{-}C1}$ cells in presence or absence of auxin (6 h), followed by DRB inhibition and release. Positions of qPCR primers are shown below. **(C)** Heatmaps sorted by gene length for DRB-4sU-seq experiments in U2OS$^{SPT6\text{-}AID\text{-}C1}$ cells in presence or absence of auxin (6 h), followed by DRB inhibition and release for 30 min over 7,562 expressed genes. Dotted lines indicate the visual wavefront. **(D)** Estimation of elongation rates by regression using linear vs. exponential functions in DRB-4sU-seq experiments for auxin-treated samples. The X-axis shows the three samples (10, 20 and 30 min DRB washout). The Y-axis shows the percentages of genes with accurate regression fit, according to how their elongation rates were estimated with the linear function. **(E)** All pairwise correlation coefficients for expression levels of total and newly transcribed RNA at different times among the three replicates of SLAM-seq experiments in U2OS$^{SPT6\text{-}AID\text{-}C1}$ cells incubated in presence or absence of auxin (6 h), followed by 4sU labeling. **(F)** Scatter plot comparing RNA half-lives for 4,533 genes, in control and auxin-treated U2OS$^{SPT6\text{-}AID\text{-}C1}$ cells, estimated by mathematical modeling using SLAM-seq data. Spearman's ρ and p values computed by asymptotic t approximation are indicated.

Figure S5

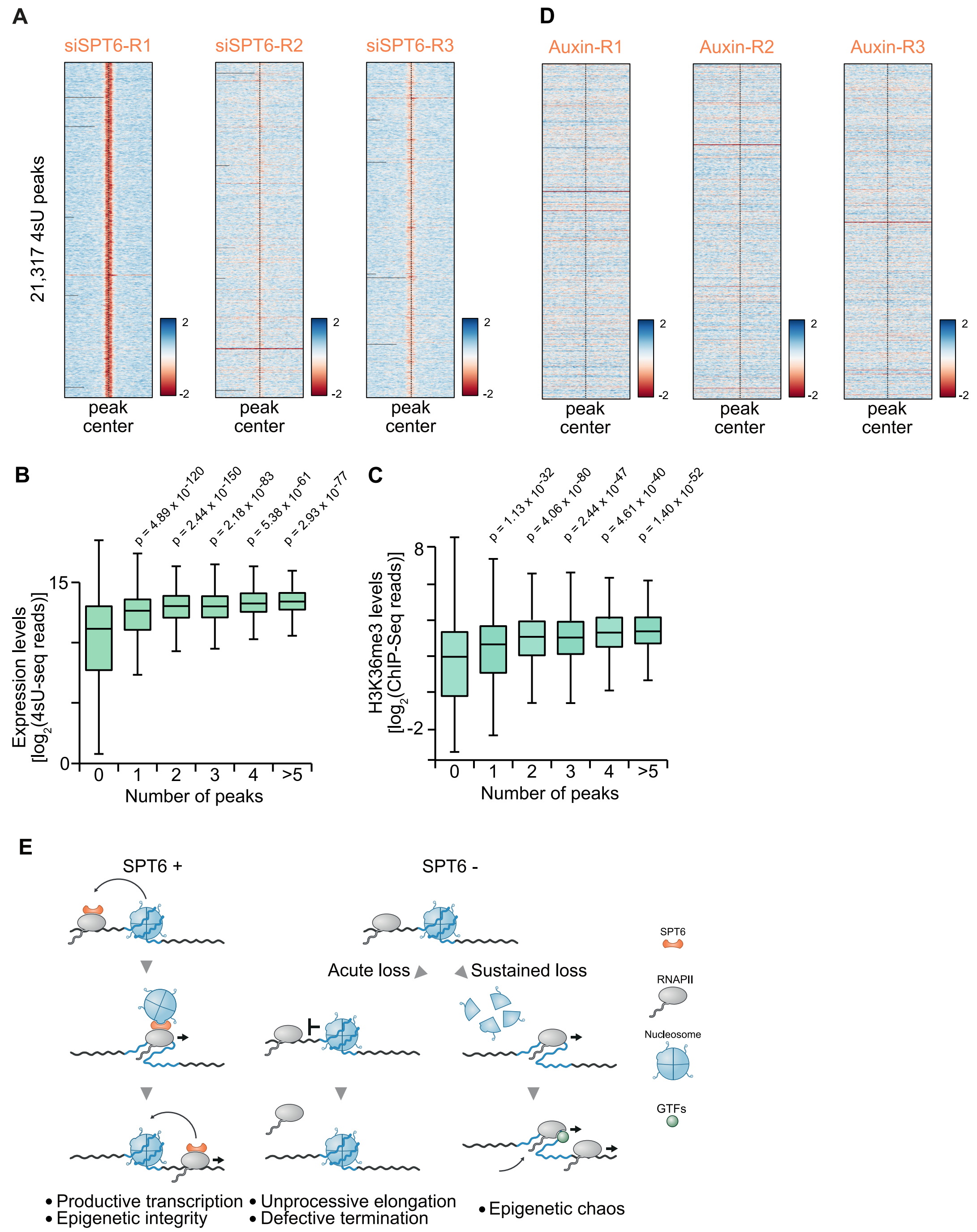
A
siSPT6-R1
siSPT6-R2
siSPT6-R3
21,317 4sU peaks
2
-2
peak center
D
Auxin-R1
Auxin-R2
Auxin-R3
B
p = 4.89 x 10-120
p = 2.44 x 10-150
p = 2.18 x 10-83
p = 5.38 x 10-61
p = 2.93 x 10-77
Expression levels [log2(4sU-seq reads)]
15
0
0
1
2
3
4
>5
Number of peaks
C
p = 1.13 x 10-32
p = 4.06 x 10-80
p = 2.44 x 10-47
p = 4.61 x 10-40
p = 1.40 x 10-52
H3K36me3 levels [log2(ChIP-Seq reads)]
8
-2
E
SPT6 +
SPT6 -
Acute loss
Sustained loss
SPT6
RNAPII
Nucleosome
GTFs
• Productive transcription
• Epigenetic integrity
• Unprocessive elongation
• Defective termination
• Epigenetic chaos

Cryptic initiation sites on sustained loss of SPT6. Related to Figure 5. (A) Relative heatmaps centered at 21,317 peaks (±2.5 kb) found in siSPT6 condition. Shown are z-scores comparing 4sU-seq reads from siSPT6-treated and siCTR-treated cells (48 h) for three replicates. **(B)** Transcription rates according to the number of 4sU peaks called in gene bodies in siSPT6-treated U2OS cells. Two-sided unpaired Wilcoxon test. **(C)** H3K36 trimethylation levels according to the number of 4sU peaks in gene bodies in siSPT6-treated U2OS cells. H3K36me3 was re-analyzed from a published dataset (Wen et al., 2014). Unpaired two-sided Wilcoxon test. **(D)** Relative heatmaps centered at 21,317 peaks (±2.5 kb) found in siSPT6 condition. Shown are z-scores comparing 4sU reads from auxin-treated (6 h) to control U2OS$^{SPT6\text{-}AID\text{-}C1}$ cells for three replicates. **(E)** Proposed model showing the state of nucleosomes and RNAPII upon acute and sustained SPT6 loss. GTFs, general transcription factors

Figure S6

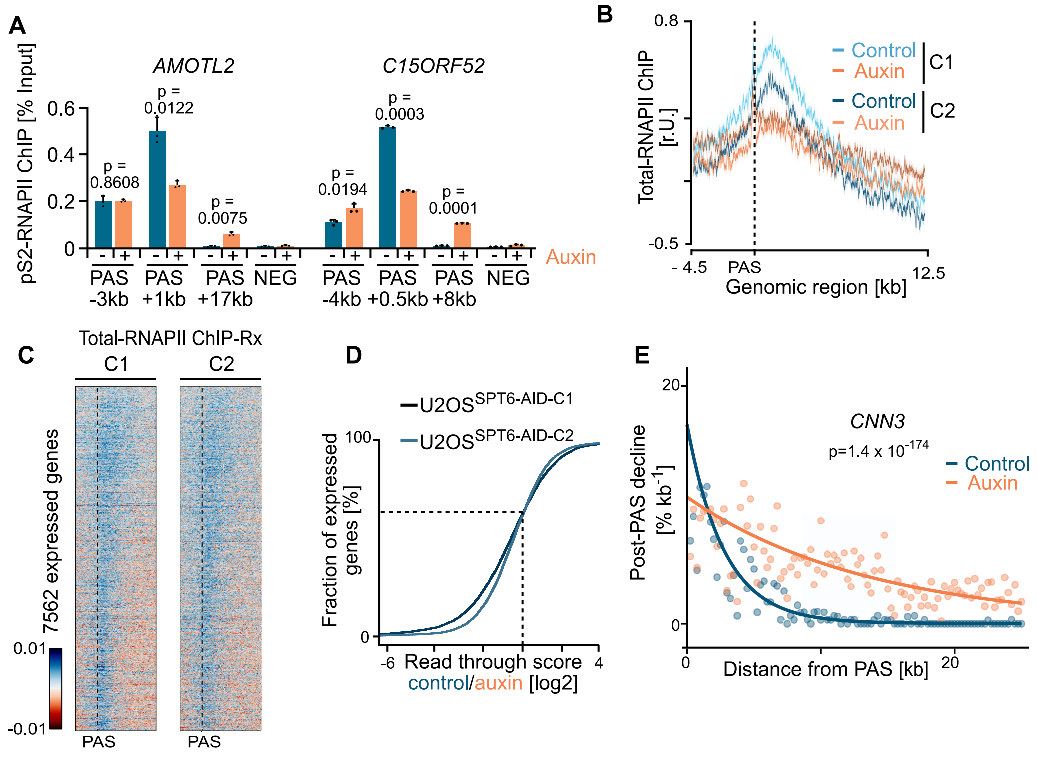

Acute SPT6 depletion causes readthrough at protein-coding genes. Related to Figure 6. **(A)** qPCR validation of pS2-RNAPII ChIP-Rx data. Graph shows occupancy at *AMOTL2* and *C15ORF52* genes (primer pairs indicated in Fig 6F), relative to PAS upstream primers. Values are mean ± SD (n=3). Two-tailed unpaired *t* test assuming equal variance. **(B)** Metagene plots showing distribution of read density from total RNAPII ChIP-Rx positioned around PAS, averaged for 4.5 kb upstream and 12.5 kb downstream in U2OS$^{SPT6\text{-}AID\text{-}C1}$ and U2OS$^{SPT6\text{-}AID\text{-}C2}$ cells in presence or absence of auxin (6 h). Shadows around curves indicate SEM values. **(C)** Relative heatmaps for read density from total RNAPII ChIP-Rx. Shown are z-scores calculated from $\log_2$ fold changes between spike-normalized reads from control and auxin-treated U2OS$^{SPT6\text{-}AID\text{-}C1}$ and U2OS$^{SPT6\text{-}AID\text{-}C2}$ cells for 7,562 expressed genes, sorted by length. Heatmaps are positioned around PAS, showing 4.5 kb upstream and 12.5 kb downstream regions. **(D)** Empirical cumulative distribution of the difference in $\log_2$ fold changes of readthrough scores based on pS2-RNAPII ChIP-Rx from control and auxin-treated cells (6 h) showing the fraction of 7,562 expressed genes. **(E)** Termination zone for example gene *CNN3.* The plots show read counts in non-overlapping 1 kb windows for the pooled 4sU-seq experiments in U2OS$^{SPT6\text{-}AID\text{-}C1}$ cells in presence or absence of auxin (6 h). Y-axis indicates read count values in the pooled 4sU-seq data. The fits of the exponential decline model are indicated as solid lines.

Figure S7

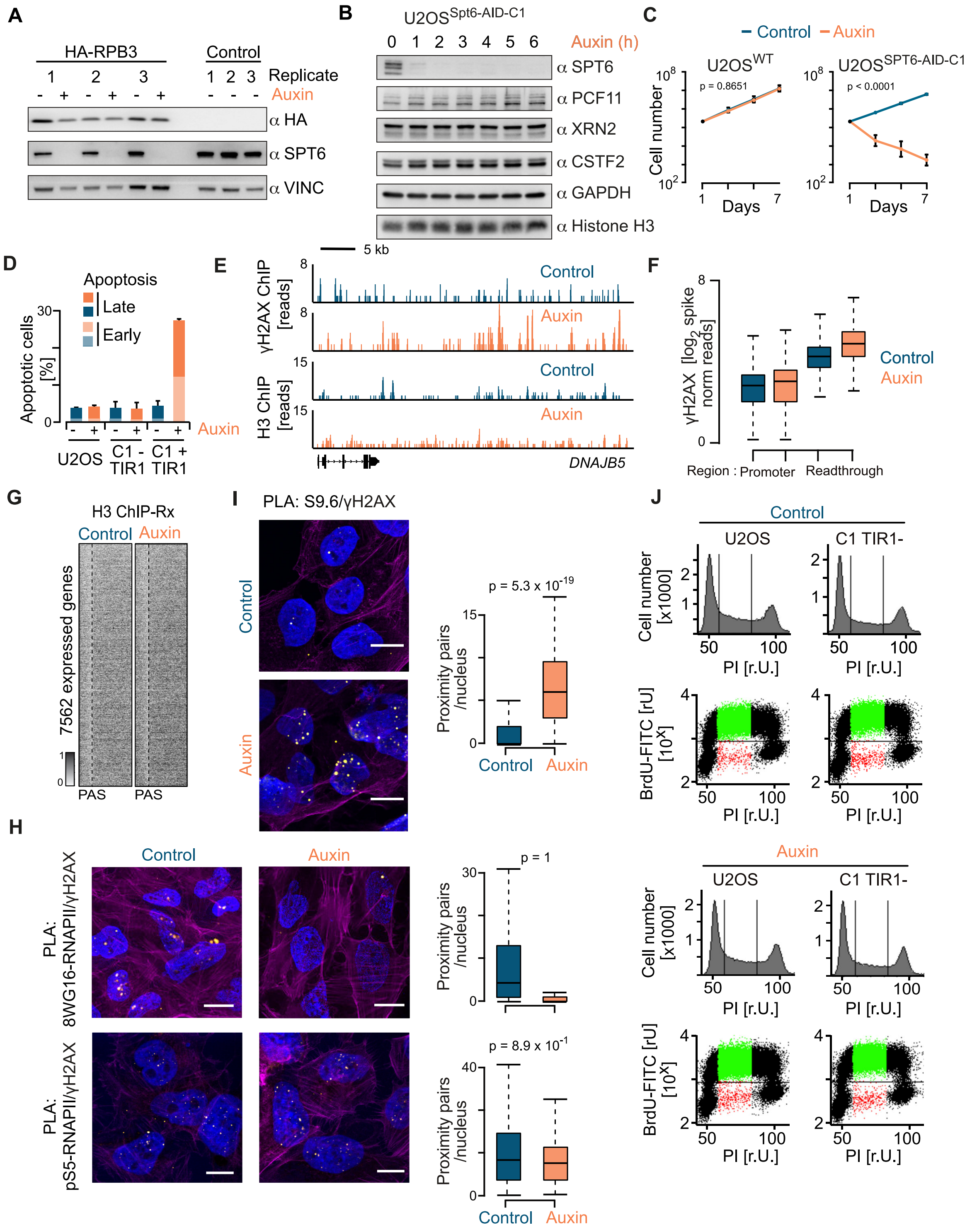

A
HA-RPB3
Control
Replicate
Auxin
α HA
α SPT6
α VINC
B
U2OS Spt6-AID-C1
Auxin (h)
α SPT6
α PCF11
α XRN2
α CSTF2
α GAPDH
α Histone H3
5 kb
C
Control
Auxin
U2OS WT
U2OS SPT6-AID-C1
p = 0.8651
p < 0.0001
Cell number
Days
D
Apoptosis
Late
Early
Apoptotic cells [%]
U2OS
C1 TIR1
Auxin
E
γH2AX ChIP [reads]
H3 ChIP [reads]
Control
Auxin
DNAJB5
F
γH2AX [log2 spike norm reads]
Region : Promoter
Readthrough
Control
Auxin
G
H3 ChIP-Rx
Control
Auxin
7562 expressed genes
PAS
PAS
I
PLA: S9.6/γH2AX
Control
Auxin
p = 5.3 x 10-19
Proximity pairs /nucleus
J
Control
U2OS
C1 TIR1-
Cell number [x1000]
PI [r.U.]
BrdU-FITC [rU] [10x]
Auxin
H
PLA: 8WG16-RNAPII/γH2AX
PLA: pS5-RNAPII/γH2AX
p = 1
p = 8.9 x 10-1
Proximity pairs /nucleus

SPT6 is essential for the recruitment of termination factors and prevents replication stress. Related to Figure 7. (A) Immunoblot of HA-RPB3-expressing and control U2OS$^{SPT6\text{-}AID\text{-}C1}$ cells. Cells were treated with auxin for 4 h and used for quantitative mass spectrometry. VINC, vinculin loading control. Membrane was cut just above the Vinculin bands. **(B)** Immunoblot of termination-associated proteins. U2OS$^{SPT6\text{-}AID\text{-}C1}$ cells expressing HA-RPB3 were treated with auxin for the indicated times. Histone H3 or GAPDH, loading control (separate membranes). **(C)** Growth curves of U2OS^{WT} and U2OS$^{SPT6\text{-}AID\text{-}C1}$ cells in presence or absence of auxin. Values are mean ± SD (n=3). Two-way ANOVA. **(D)** Annexin V-PI assay. Cells were treated or not with auxin (24 h), stained with annexin V, Pacific Blue conjugate and propidium iodide (PI), and counted by flow cytometry (50,000 sorted events). Early apoptosis, annexin+ PI; late apoptosis, annexin+ PI+. Values are mean ± SD, n=3. **(E)** Browser tracks showing γH2AX and histone-H3 ChIP-Rx experiments for *DNAJB5* gene in U2OS$^{SPT6\text{-}AID\text{-}C1}$ cells in presence or absence of auxin (24 h). **(F)** Box plot of γH2AX ChIP-Rx reads compared between auxin and control in the promoter and readthrough region, displayed across 7,562 expressed genes. **(G)** Heatmaps of histone-H3 ChIP-Rx experiments in U2OS$^{SPT6\text{-}AID\text{-}C1}$ cells in presence or absence of auxin (24 h), around PAS, averaged for 4.5 kb upstream and 12.5 kb downstream over 7,562 expressed genes, sorted by length. **(H)** Immunofluorescence images (left) of proximity ligation assays (PLAs) between unphosphorylated-RNAPII (top), and pS5-RNAPII (bottom) and γH2AX in U2OS$^{SPT6\text{-}AID\text{-}C1}$ cells treated with or without auxin. Quantification (right) of PLA foci per nucleus from the corresponding conditions. Unpaired one-sided Wilcoxon test for the likelihood of auxin being more than control is calculated. **(I)** Immunofluorescence images (left) of PLAs between S9.6 (RNA-DNA hybrid) and H2AX in U2OS$^{SPT6\text{-}AID\text{-}C1}$ cells treated or not with auxin (scale bar: 15 µm). Quantification (right) of PLA foci per nucleus. Unpaired two-sided Wilcoxon test. **(J)** Cell cycle distribution assay. Cells were treated or not with auxin (24 h), labeled with BrdU, stained with PI, and analyzed by flow cytometry. Shown are the amount of intercalating PI (top) and the correlation of BrdU to PI (bottom). Cells that are BrdU-positive in S-phase are marked in green, while those that are negative are red.
