# Supplementary material for: Targeted protein degradation reveals a direct role of SPT6 in RNAPII elongation and termination
Source: Mol Cell. 2021 Aug 5;81(15):3110–3127.e14. doi: 10.1016/j.molcel.2021.06.016 (PMC8354102; doi:10.1016/j.molcel.2021.06.016)
Supplement: Table S4. Sequences of oligonucleotides, Related to STAR Methods [file mmc5.docx]

| **OLIGONUCLEOTIDES** | **SEQUENCE** | **SOURCE** |
| --- | --- | --- |
| **Oligos for cloning, knock-in and genotyping** | | |
| cSPT6_5HA_AgeI_F | CGCACCGGTCTCTGAGGGCCCTGACCACA | This paper |
| cSPT6_5HA_EcoRI_R | CGGAATTCCCGATCCATCTCGTCCAGGA | This paper |
| cSPT6_3HA_BamHI_F | CGGGATCCGGGGCCTGCTCCTCGGACTC | This paper |
| cSPT6_3HA_SpeI_R | GGACTAGTAGGGTCACCCGGCTGGGAAG | This paper |
| cSPT6_2055_F | GATCTTTGGAACCCGCAAGC | This paper |
| cSPT6_2055_R | TGGGGCAGGAACACCTTACT | This paper |
| TIR1_AgeI_F | CGCACCGGTATGACGTACTTCCCG | This paper |
| TIR1_MluI_R | CGCGACGCGTTTAGCTAGTGGATCCGTT | This paper |
| cSPT6_sg1_T | CACCGTGGACGAGATGGATCGGTAG | This paper |
| cSPT6_sg1_B | AAACCTACCGATCCATCTCGTCCAC | This paper |
| cSPT6_sg2_T | CACCGCCTGGACGAGATGGATCGGT | This paper |
| cSPT6_sg2_B | AAACACCGATCCATCTCGTCCAGGC | This paper |
| **Oligos for qPCR assays** | | |
| OPA1+2kb_F | ACCATGGATGCCATTGAGTCA | Fuchs et al., 2014 |
| OPA1+2kb_R | TGTGCCATCACCAGGAGACAT | Fuchs et al., 2014 |
| OPA1+22kb_F | AGATGTGAAAGTCAGGTGGCAT | Fuchs et al., 2014 |
| OPA1+22kb_R | CAGACCTGCCAAGAAGTGCTT | Fuchs et al., 2014 |
| OPA1+28kb_F | GTGGCCAGGCTTTAGCTGAAT | Fuchs et al., 2014 |
| OPA1+28kb_F | CAGCCCTCAGACTGAACCAGTT | Fuchs et al., 2014 |
| RT-b2MGF | GTGCTCGCGCTACTCTCTC | Baluapuri et al., 2019 |
| RT-b2MGR | GTCAACTTCAATGTCGGAT | Baluapuri et al., 2019 |
| Primer qPCR NegReg_F | TTTTCTCACATTGCCCCTGT | Baluapuri et al., 2019 |
| Primer qPCR NegReg_R | TCAATGCTGTACCAGGCAAA | Baluapuri et al., 2019 |
| Primer qPCR ACTB_TSS_F | CGTTCCGAAAGTTGCCTTTT | This paper |
| Primer qPCR ACTB_TSS_R | GCCGCTGGGTTTTATAGGG | This paper |
| Primer qPCR ACTB_TES_F | CCTTGAGTGGGGGTGTAGTG | This paper |
| Primer qPCR ACTB_TES_R | CCACGATCCCATAGGTGAAG | This paper |
| Primer qPCR RPS9_F | GACGGTGGTCTTGTGTCCAT | This paper |
| Primer qPCR RPS9_R | GGAAGGAGACCACAGTGAGC | This paper |
| Primer qPCR NCL_F | TACTGGGCAGGCTCAGTCTT | Baluapuri et al., 2019 |
| Primer qPCR NCL_R | GAAGATCCCGGAGCACGTA | Baluapuri et al., 2019 |
| Primer qPCR NPM1_F | TTCACCGGGAAGCATGG | This paper |
| Primer qPCR NPM1_R | CACGCGAGGTAAGTCTACG | This paper |
| Primer_readthrough_qPCR_prePAS dnajb11_rt_p1_F | GCTTTGTGGGACATGTATGTGG | This paper |
| Primer_readthrough_qPCR_prePAS dnajb11_rt_p1_R | AGGTAACCCCACACATGCAC | This paper |
| Primer_readthrough_qPCR_postPAS dnajb11_rt_p2_F | TCTTCTGATtgccaggcact | This paper |
| Primer_readthrough_qPCR_postPAS dnajb11_rt_p2_R | tgtttgtcttgcttgctggc | This paper |
| Primer_readthrough_qPCR_prePAS prpf38b_rt_p1_F | AGAGGGCATAGCAAGAAGGC | This paper |
| Primer_readthrough_qPCR_prePAS prpf38b_rt_p1_R | TGCCATGAAGCAGTGAGGTT | This paper |
| Primer_readthrough_qPCR_postPAS prpf38b_rt_p2_F | ggtctggaactcttgggctc | This paper |
| Primer_readthrough_qPCR_postPAS prpf38b_rt_p2_R | ACTTCCTATTTGggcccagc | This paper |
| Primer_readthrough_qPCR_prePAS amotl2_rt_p1_F | AAGTGCCTCCCATCTGGTTG | This paper |
| Primer_readthrough_qPCR_prePAS amotl2_rt_p1_R | ACATGAGGGATGGCTGGTTG | This paper |
| Primer_readthrough_qPCR_postPAS amotl2_rt_p2_F | GTGAGAGGGAGGGCAACTTC | This paper |
| Primer_readthrough_qPCR_postPAS amotl2_rt_p2_R | ACCACCCTCACCTGCTATCT | This paper |
| Primer_processivity_qPCR_TSS peak1_prd_p1_f | TGGGCTGAGGCTATAACCCT | This paper |
| Primer_processivity_qPCR_TSS peak1_prd_p1_r | TGCCTGGGACTTCTCTTTGG | This paper |
| Primer_processivity_qPCR_PAS peak1_prd_p2_f | AGTTTTACTTTGCGAGCAGGA | This paper |
| Primer_processivity_qPCR_PAS peak1_prd_p2_r | ATGAGCCACCACCCAGATTG | This paper |
| Primer_processivity_qPCR_TSS fam208b_prd_p1_f | catgcgtgtgatgctagcac | This paper |
| Primer_processivity_qPCR_TSS fam208b_prd_p1_r | tcactatgttgcccaggctg | This paper |
| Primer_processivity_qPCR_PAS fam208b_prd_p2_f | AAAGCAGACCTGACAAGCCC | This paper |
| Primer_processivity_qPCR_PAS fam208b_prd_p2_r | agctcctggggatgaagtct | This paper |
| Primer_pS2_ChIP_qPCR amotl2_3pPaus_F | GCAGCTCACTCATCCCCTTT | This paper |
| Primer_pS2_ChIP_qPCR amotl2_3pPaus_R | CTTCTGGCTAGTGTCCCTGC | This paper |
| Primer_pS2_ChIP_qPCR amotl2_prePAS_F | AAGTGCCTCCCATCTGGTTG | This paper |
| Primer_pS2_ChIP_qPCR amotl2_prePAS_R | ACATGAGGGATGGCTGGTTG | This paper |
| Primer_pS2_ChIP_qPCR amotl2_RT_F | TTCCCTCTTCTCCACCTGGT | This paper |
| Primer_pS2_ChIP_qPCR amotl2_RT_R | GGCCTTTGAGTACCAGGTCC | This paper |
| Primer_pS2_ChIP_qPCR c15orf52_3pPaus_F | CCAGTTCCTGTCCCTTGACC | This paper |
| Primer_pS2_ChIP_qPCR c15orf52_3pPaus_R | CCCCAGAAACAAACCCCCTT | This paper |
| Primer_pS2_ChIP_qPCR c15orf52_prePAS_F | GTCTCTCTGGTGCTTTGGCT | This paper |
| Primer_pS2_ChIP_qPCR c15orf52_prePAS_R | GACAAGGAGGAGCTGGAAGG | This paper |
| Primer_pS2_ChIP_qPCR c15orf52_RT_F | agatggtctttgggctgacg | This paper |
| Primer_pS2_ChIP_qPCR c15orf52_RT_R | acagaatgggccccaaaagt | This paper |
