# Supplementary material for: Targeted protein degradation reveals a direct role of SPT6 in RNAPII elongation and termination
Source: Mol Cell. 2021 Aug 5;81(15):3110–3127.e14. doi: 10.1016/j.molcel.2021.06.016 (PMC8354102; doi:10.1016/j.molcel.2021.06.016)
Supplement: Document S2. Article plus supplemental information [file mmc6.pdf]

Molecular Cell

Article

Targeted protein degradation reveals a direct role of SPT6 in RNAPII elongation and termination

Graphical abstract

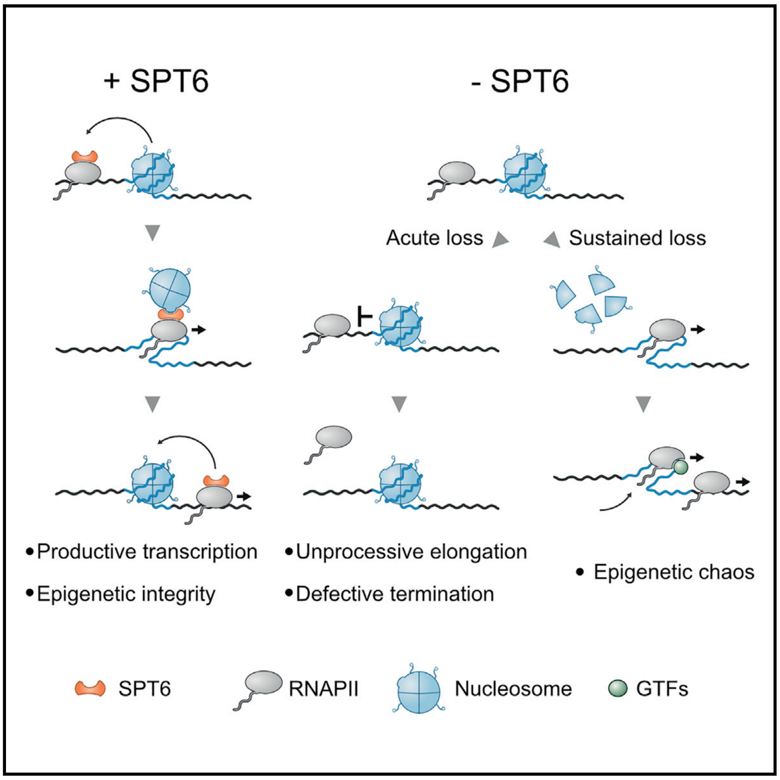

Authors

Ashwin Narain, Pranjali Bhandare, Bikash Adhikari, ..., Florian Erhard, Apoorva Baluapuri, Elmar Wolf

Correspondence

florian.erhard@uni-wuerzburg.de (F.E.), apoorva.baluapuri@biozentrum.uni-wuerzburg.de (A.B.), elmar.wolf@biozentrum.uni-wuerzburg.de (E.W.)

In brief

Histone chaperone SPT6 is essential for maintaining epigenetic integrity in the wake of transcription by RNAP II. By studying the effects of targeted acute depletion of SPT6 with multi-omics and mathematical modeling, Narain et al. uncovered direct roles of SPT6 in ensuring efficient transcription elongation and termination in human cells.

Highlights

- Auxin-inducible degradation discriminates direct roles of human SPT6 in transcription
- Acute loss of SPT6 globally impairs RNAPII processivity and speed
- SPT6 is required for efficient transcription termination on protein-coding genes
- Long-term loss of SPT6 ultimately results in cryptic intragenic transcription

Narain et al., 2021, Molecular Cell *81*, 3110–3127
August 5, 2021 © 2021 The Author(s). Published by Elsevier Inc.
https://doi.org/10.1016/j.molcel.2021.06.016

CellPress

CellPress
OPEN ACCESS

Molecular Cell

Article

Targeted protein degradation reveals a direct role of SPT6 in RNAPII elongation and termination

Ashwin Narain,[1,7] Pranjali Bhandare,[1,7] Bikash Adhikari,[1] Simone Backes,[2] Martin Eilers,[3] Lars Dölken,[2] Andreas Schlosser,[4] Florian Erhard,[5,*] Apoorva Baluapuri,[1,*] and Elmar Wolf[1,6,8,*]

[1]Cancer Systems Biology Group, Theodor Boveri Institute, University of Würzburg, Am Hubland, 97074 Würzburg, Germany
[2]Institute for Virology and Immunobiology, University of Würzburg, Versbacher Straße 7, 97078 Würzburg, Germany
[3]Department of Biochemistry and Molecular Biology, Theodor Boveri Institute, University of Würzburg, Am Hubland, 97074 Würzburg, Germany
[4]Rudolf Virchow Center, Center for Integrative and Translational Bioimaging, University of Würzburg, Josef-Schneider-Straße 2, 97080 Würzburg, Germany
[5]Computational Systems Virology and Bioinformatics, Institute for Virology and Immunobiology, University of Würzburg, Versbacher Straße 7, 97078 Würzburg, Germany
[6]Mildred Scheel Early Career Center, University of Würzburg, Beethovenstraße 1A, 97080 Würzburg, Germany
[7]These authors contributed equally
[8]Lead contact
*Correspondence: florian.erhard@uni-wuerzburg.de (F.E.), apoorva.baluapuri@biozentrum.uni-wuerzburg.de (A.B.), elmar.wolf@biozentrum.uni-wuerzburg.de (E.W.)
https://doi.org/10.1016/j.molcel.2021.06.016

SUMMARY

SPT6 is a histone chaperone that tightly binds RNA polymerase II (RNAPII) during transcription elongation. However, its primary role in transcription is uncertain. We used targeted protein degradation to rapidly deplete SPT6 in human cells and analyzed defects in RNAPII behavior by a multi-omics approach and mathematical modeling. Our data indicate that SPT6 is a crucial factor for RNAPII processivity and is therefore required for the productive transcription of protein-coding genes. Unexpectedly, SPT6 also has a vital role in RNAPII termination, as acute depletion induced readthrough transcription for thousands of genes. Long-term depletion of SPT6 induced cryptic intragenic transcription, as observed earlier in yeast. However, this phenotype was not observed upon acute SPT6 depletion and therefore can be attributed to accumulated epigenetic perturbations in the prolonged absence of SPT6. In conclusion, targeted degradation of SPT6 allowed the temporal discrimination of its function as an epigenetic safeguard and RNAPII elongation factor.

INTRODUCTION

Transcription of genes by RNA polymerase II (RNAPII) is controlled largely by chromatin organization. The distribution and composition of nucleosomes in the genome define transcriptional units and dictate how, when, and to what extent they are transcribed (Gates et al., 2017). Importantly, gene promoters are devoid of nucleosomes, and promoter-proximal nucleosomes have specific modifications that enable RNAPII initiation (Lorch and Kornberg, 2015). In contrast, nucleosomes in gene bodies contain histone modifications that ensure processive transcription elongation and inhibit undesirable transcription initiation (Neri et al., 2017). Although chromatin organization is crucial for transcription regulation, the process of transcription itself presents a great threat to it. As transcription requires the melting of the DNA double helix, every transcription cycle can result in nucleosome disassembly or incorporation of incorrectly modified histones and may ultimately result in transcription chaos (Venkatesh and Workman, 2015).

To maintain epigenetic information during transcription, histone chaperones travel with RNAPII and ensure the correct reassembly of nucleosomes with DNA during transcription elongation (Eitoku et al., 2008; Viktorovskaya et al., 2021). SPT6 is a RNAPII-associated histone chaperone that is conserved in eukaryotes (Andrulis et al., 2000; Eitoku et al., 2008; Kaplan et al., 2000; Ni et al., 2008). SPT6 was discovered in a pioneering genetic screen for mutations that affect Ty element-mediated expression of the *HIS4* gene in yeast (Winston et al., 1984). Soon after its discovery, it was observed that yeast Spt6 physically interacts with histone H3 (Bortvin and Winston, 1996; McCullough et al., 2015) and preserves nucleosome positions, histone variants, and histone modifications in gene bodies (DeGennaro et al., 2013; Hainer and Martens, 2016; Jeronimo et al., 2015, 2019; Kato et al., 2013; Nojima et al., 2018; Youdell et al., 2008).

Depletion of histone chaperones could have drastic consequences on transcription. Indeed, knockdown of SPT6 results in spurious intragenic transcription initiation (Doris et al., 2018;

 © 2021 The Author(s). Published by Elsevier Inc.
This is an open access article under the CC BY license (http://creativecommons.org/licenses/by/4.0/).

CellPress
OPEN ACCESS

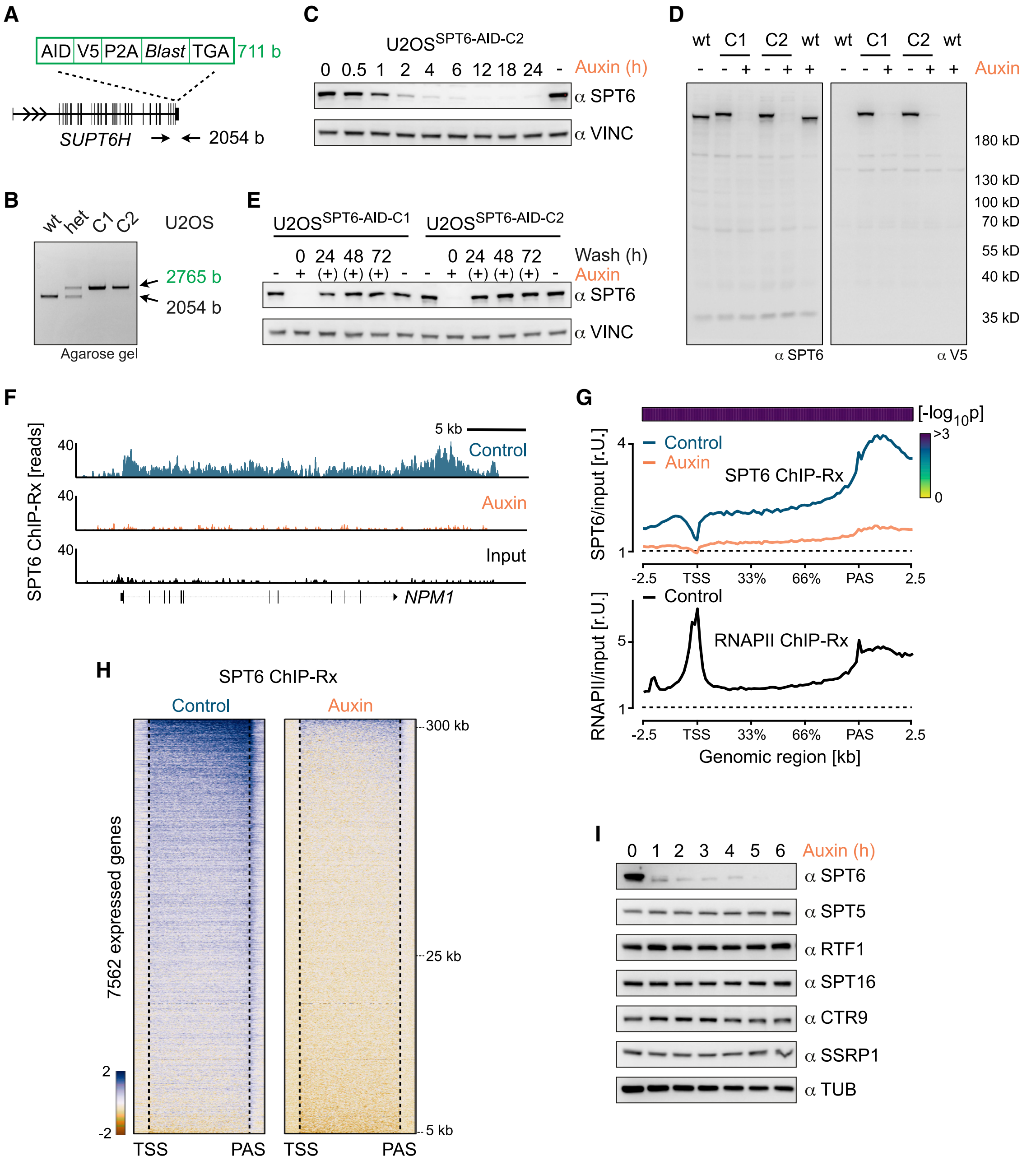

Figure 1. Auxin-mediated depletion of SPT6 is rapid and specific

(A) Schematic of the knockin strategy for AID-tagged SPT6. Shown are components of the knockin cassette. Positions of primers for genomic PCR are marked by arrows.

(B) Agarose gel of PCR from U2OS$^{SPT6-AID}$ knockin clones. wt, wild-type; het, heterozygous; C1 and C2, homozygous clones.

(C) Immunoblot of SPT6 in U2OS$^{SPT6-AID-C2}$ cells treated with auxin for the indicated times. Vinculin, loading control.

(D) Full-membrane immunoblots. U2OS$^{SPT6-AID-C1}$ and U2OS$^{SPT6-AID-C2}$ cells were treated with auxin, and the migration of SPT6 was compared with that in wild-type U2OS cells using antibodies for SPT6 and V5.

(E) Immunoblot of SPT6 in U2OS$^{SPT6-AID}$ cells treated with auxin and then incubated in fresh medium for the indicated times. Vinculin, loading control.

(F) Browser tracks of *NPM1* gene showing SPT6 ChIP-Rx reads from U2OS$^{SPT6-AID-C1}$.

(G) Metagene plots from SPT6 ChIP-Rx and total-RNAPII. Input-normalized reads averaged over all expressed genes are shown. p values (two-sided Wilcoxon test) for the difference (auxin/control), calculated from the density values of individual genes at each genomic location, are shown in a heatmap.

(legend continued on next page)

Gouot et al., 2018; Jeronimo et al., 2015; Kaplan et al., 2003; Uwimana et al., 2017), aberrant levels of antisense transcription (DeGennaro et al., 2013; Dronamraju et al., 2018), pre-mature termination (Pathak et al., 2018), and changes in RNAPII elongation rates (Ardehali et al., 2009; Hartzog et al., 1998; Perales et al., 2013).

SPT6 not only binds to histones but also interacts directly with the RNAPII core complex (Brázda et al., 2020; Farnung et al., 2018; Sdano et al., 2017; Vos et al., 2018; Yoh et al., 2007). The tight interaction between SPT6 and RNAPII suggests that SPT6 also has direct effects on transcription. This hypothesis is supported by crystal structures of SPT6 that demonstrate remarkable similarity to the bacterial protein Tex, which functions in transcriptional processes in nucleosome-free environments (Close et al., 2011; Johnson et al., 2008). Moreover, human SPT6 stimulates RNAPII transcription elongation *in vitro* on non-chromatinized templates (Endoh et al., 2004). However, the primary function of SPT6 in transcription is uncertain, as previous studies used hypomorphic mutants or long-term depletion of SPT6, which are not ideal for assessing direct effects on transcription.

To elucidate the primary functions of SPT6 in transcription, we established a system for its acute depletion in human U2OS cells. In contrast to long-term knockdown of SPT6, acute depletion did not result in cryptic intragenic transcription initiation. Instead, we observed a drastic loss of processive RNAPII elongation and, unexpectedly, a strong increase in readthrough transcription downstream of the polyadenylation site (PAS). Mathematical modeling revealed that all observed defects together result in drastically reduced RNA synthesis rates for coding genes in the absence of SPT6.

RESULTS

Auxin-mediated depletion of SPT6 is rapid and specific

To acutely deplete SPT6 in cells, we used the auxin-inducible degron (AID) system in which AID-tagged proteins are directed to proteasomal degradation by the E3 ligase TIR1 in the presence of the plant hormone auxin (Nishimura et al., 2009). For this purpose, we integrated the AID sequence at the SPT6 locus (*SUPT6H*) in the U2OS human osteosarcoma cell line by CRISPR-Cas9-mediated genome editing (Figures 1A and 1B). We confirmed correct homozygous integration using Sanger sequencing (Figure S1A) and immunoblotting (Figure S1B) and selected two clones (U2OS$^{\text{SPT6-AID-C1}}$ and U2OS$^{\text{SPT6-AID-C2}}$). We stably expressed TIR1 in both clones and induced SPT6 degradation by treating cells with auxin. Immunoblotting demonstrated decreased protein levels already 1 h after auxin addition and almost undetectable levels at later times (Figures 1C, 1D, and S1C). The depletion was reversible, as SPT6 levels in auxin-treated cells recovered 24 h after auxin washout (Figure 1E). Replicate immunoblots revealed that 4–6 h after auxin addition, cellular SPT6 levels were depleted by 94% in U2OS$^{\text{SPT6-AID-C1}}$ and 91% in U2OS$^{\text{SPT6-AID-C2}}$ cells (Figure S1D). Further experiments were analyzed in this time frame.

To assess depletion of chromatin-bound SPT6, we performed spiked chromatin immunoprecipitation (ChIP) sequencing (ChIP with a reference exogenous genome [ChIP-Rx]) of SPT6 in U2OS$^{\text{SPT6-AID-C1}}$ cells. Inspection of individual genes (Figure 1F) and metagene and heatmap analyses (Figures 1G and 1H) revealed that in control cells, SPT6 binding started after the transcriptional start site (TSS) and extended throughout the entire transcription unit. Notably, the SPT6 signal began to increase before PAS and peaked just after it, indicating that SPT6 remained associated with RNAPII during transcript cleavage and termination. In auxin-treated cells, ChIP-Rx revealed the highly efficient, uniform depletion of chromatin-bound SPT6 (Figures 1F–1H). Auxin treatment did not reduce levels of other SPT6-associated proteins (Figure 1I). We concluded that auxin-mediated depletion of cellular SPT6 is fast and complete, without the indiscriminate depletion of SPT6-associated proteins in U2OS$^{\text{SPT6-AID}}$ cells.

Acute depletion of SPT6 interferes with transcription elongation

To assess the acute effects of SPT6 depletion on transcription, we labeled newly synthesized transcripts by incubating U2OS$^{\text{SPT6-AID-C1}}$ cells in medium containing 4-thiouridine (4sU) for 15 min and sequenced 4sU-labeled RNA of three biological replicates (Figure 2A). Metagene analysis of all expressed genes revealed that the distribution of reads over the gene body differed substantially between SPT6-depleted (auxin-treated) and control cells. In auxin-treated cells, we observed a drastic reduction of the 4sU signal toward PAS, suggesting an essential direct function of SPT6 in elongation (Figure 2A). The read distribution of untreated U2OS$^{\text{SPT6-AID-C1}}$ cells was indistinguishable from that of auxin-treated wild-type U2OS cells (Figure S2A), indicating that neither auxin nor SPT6 tagging caused the observed effects on transcription.

Intriguingly, inspection of individual genes revealed that the drop in 4sU sequencing (4sU-seq) read density differs according to gene length (Figure 2B): For long genes such as *PEAK1*, we observed a drastic reduction of 4sU-seq reads relatively close to TSS upon SPT6 depletion, with almost no reads detectable in distal gene regions. For medium-sized genes such as *ABHD17B*, read densities declined progressively over the whole gene body. For short genes such as *SNRPB*, read coverage did not noticeably decline in gene bodies but increased substantially downstream of PAS (a result we analyze in detail later). Heatmap and metagene analyses confirmed that SPT6-depleted cells had a global elongation defect, which was most pronounced at long genes (Figures 2C, S2B, and S2C).

To quantify the elongation defects at the gene level, we calculated a transcription completion score as the ratio of the 4sU-seq read density in the last 15% of the gene body to that in the first 15% (Figures 2D and 2E; Table S1). Overall, 1,579 of all 7,505

(H) Heatmaps showing *Z* scores calculated from input-normalized SPT6 ChIP-Rx reads in the presence (right) or absence (left) of auxin for 7,562 expressed genes (>5 kb), sorted by length.

(I) Immunoblot of SPT6-interacting proteins. U2OS$^{\text{SPT6-AID-C1}}$ cells were treated with auxin, and SPT6-associated proteins were analyzed on separate membranes. Tubulin, loading control.

See also Figure S1.

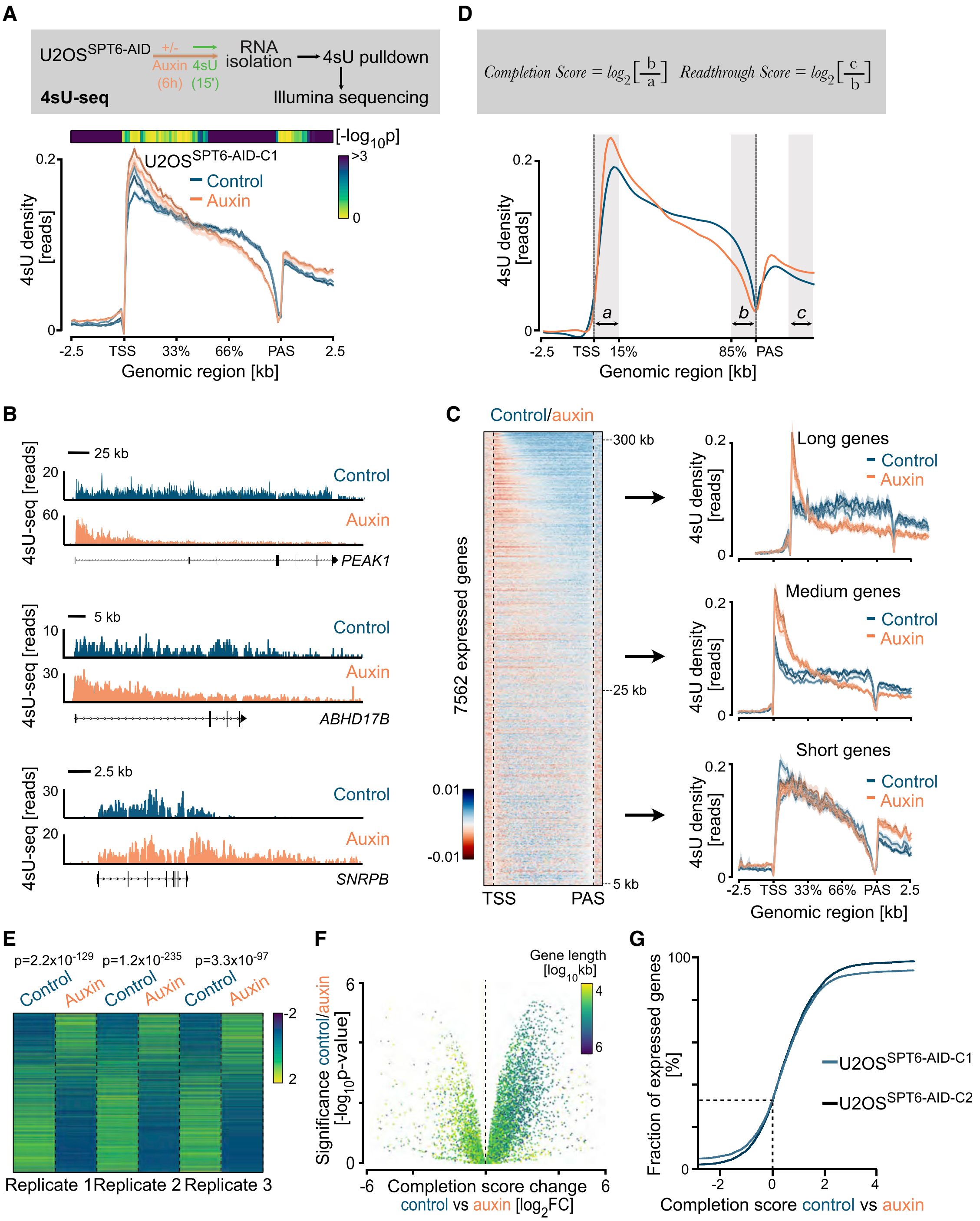

Figure 2. Acute SPT6 depletion interferes with transcription elongation

(A) Experimental scheme and metagene analysis of 4sU-seq experiments. Metagene plot showing the distribution of read density over the gene body averaged over 7,562 expressed genes in U2OS$^{SPT6-AID-C1}$ cells, for three biological replicates with or without auxin. Shadows around curves indicate SEM. p values (two-sided Wilcoxon test) for the difference (auxin/control), calculated from the density values of individual genes at each genomic location, are shown in a heatmap.

(B) Browser tracks from a 4sU-seq experiment in U2OS$^{SPT6-AID-C1}$ cells. Shown are examples of long (*PEAK1*), medium (*ABHD17B*), and short (*SNRPB*) genes. The 4sU tracks contain gaps at exon locations, as only intronic 4sU reads were considered.

(C) Heatmap and metagene analyses of 4sU-seq. Relative and scaled heatmap (left) showing *Z* scores between control and auxin-treated U2OS$^{SPT6-AID-C1}$ cells from 4sU-seq reads over gene bodies sorted by length. Orange indicates less and blue indicates more reads in control cells. Metagene plots (right) showing the distribution of read density for sets of long, medium, and short genes. Shadows around curves indicate SEM. More metagene plots are in Figure S2C.

(legend continued on next page)

expressed genes had significantly decreased completion scores ($q < 0.05$, Bayes moderated t test), indicating defective transcription completion (Figure 2F). In contrast, only 356 of all expressed genes showed increased transcription completion in the absence of SPT6. We confirmed the decrease in transcription completion by quantitative PCR (qPCR) on total RNA with a proximal and distal intronic primer pair for two genes (Figures S2D and S2E). Finally, we performed 4sU-seq in the other knockin clone ($U2OS^{SPT6\text{-}AID\text{-}C2}$) to exclude that the observed defect in transcription completion was limited to cells of a certain clonal origin (Figures 2G and S2F). In addition to the marked drop in transcription completion in the sense direction, we also observed reduced antisense transcription at bi-directional promoters (Figures S2G and S2H; Table S2). We concluded that acute SPT6 depletion hinders completion of transcription at most protein-coding genes.

Defective transcription completion results from processivity loss due to SPT6 depletion

Transcriptional activity along gene bodies is determined by the interplay of the successful initiation, elongation rate, and processivity (Gressel et al., 2019; Wissink et al., 2019). We defined the successful initiation rate as the number of initiating RNAPII molecules that progress to elongation per minute. The elongation rate is the number of nucleotides transcribed per minute, and processivity is the number of nucleotides transcribed per RNAPII molecule. We used an ordinary differential equation (ODE) model to simulate RNAPII transcription coverage for the 4sU-seq experiments. Simulations suggested that both slow elongation and reduced processivity, but not defective initiation, can result in a decline of 4sU read density over gene bodies (Figure 3A).

To differentiate between these two potential causes of the observed 4sU read density decline in SPT6-depleted cells, we further examined the 4sU-seq data. To quantify the effect along gene bodies, we considered read densities in non-overlapping 1 kb windows and computed a local fold change (LFC) of auxin versus control cells in each window. Analyzing LFCs circumvents confounders such as length bias, labeling time, and differential splicing kinetics (Erhard and Zimmer, 2015). Interestingly, inspection of individual genes revealed a linear decline of LFCs along the gene body (Figure 3B). This decline is compatible with a defect in processivity at a constant rate throughout the gene body; that is, in the absence of SPT6, for each kilobase transcribed, transcription is aborted with a small, constant probability. Of note, for long genes we consistently observed a marked turning point around 90 kb after the TSS, downstream of which fold changes (FCs) stayed constant, likely corresponding to a reduction of transcription to background levels in SPT6-depleted cells (Figure S3A).

To quantify the processivity defect among all expressed genes, we did a regression analysis on the basis of LFC statistics (Erhard and Zimmer, 2015). For 5,897 genes longer than 25 kb, we obtained an accurate fit with a 95% confidence interval (CI) < 1%/kb (Figure S3B), demonstrating a global linear decline in LFCs along genes. Taking the slope of the regression as the processivity defect for each gene, we calculated a median defect of −0.97%/kb. This continuous decline was largely independent of expression levels and gene length (Figure 3C). However, because this processivity defect accumulates over gene length, this finding confirms our earlier observation that the total decline in 4sU reads is strongest for long genes. Thus, LFC regression provided direct evidence that acute SPT6 depletion results in reduced RNAPII processivity for all expressed genes, by favoring the stochastic abortion of transcription.

The ODE model suggested that comparing 4sU-seq with RNAPII ChIP-Rx can discriminate between effects on RNAPII elongation rates and processivity (Figure 3A). We therefore did ChIP-Rx experiments using a total-RNAPII antibody and observed a typical profile of RNAPII occupancy, that is, a sharp peak at the TSS proximal pause site, low density in the gene body, and accumulation after PAS (Figure 3D). In SPT6-depleted cells, the read density also peaked around the pause site, but in the gene body it declined to a greater degree and crossed under the control cell signal (Figure 3D). Although this observation confirms the processivity defect in SPT6-depleted cells, the high enrichment of paused RNAPII hinders interpretations on RNAPII occupancy (Chen et al., 2018) in gene bodies. Thus, we repeated RNAPII ChIP-Rx experiments with an antibody that recognizes the elongating form of RNAPII (pS2). Metagene analyses showed a pS2-RNAPII profile in control cells that gradually increased over the gene body and strongly accumulated after PAS (Figure 3E). In SPT6-depleted cells, the pS2-RNAPII ChIP-Rx signal dropped drastically toward PAS (Figure 3E). Heatmaps demonstrated this effect for all expressed genes (Figure 3F). This drop in RNAPII density upon SPT6 depletion was confirmed in RNAPII and pS2-RNAPII ChIP-Rx experiments in the second cell clone as well (Figure S3C).

To quantitatively explore the RNAPII ChIP-Rx data, we again performed LFC analysis and observed a behavior reminiscent of the 4sU-seq data (Figures 3G and S3D). Strikingly, the slopes of the LFC regression from pS2-RNAPII ChIP-Rx and 4sU-seq were not only strongly correlated ($R = 0.62$, $p = 2.1 \times 10^{-252}$), but also of the same magnitude. The correlation was low for only a subset of weakly expressed or short genes, most likely because of imprecise estimations from few reads (Figures 3H and S3E). This finding provides strong evidence that the decline in 4sU-seq signal is attributable to a RNAPII processivity defect and rules out the possibility that changes in RNA processing caused the decline in 4sU-seq signal.

(D) Spline-smoothed metagene plot from 4sU-seq experiments combined from all replicates in the presence of auxin. The genomic regions indicated were used to calculate completion and readthrough scores.

(E) Heatmap of *Z* scores calculated from completion scores for three replicates overexpressed genes. Negative values (blue) imply less completion of transcription. Two-sided Wilcoxon test.

(F) Volcano plot comparing $\log_2$ fold changes and the statistical significance of completion scores calculated in control and auxin conditions for expressed genes. Bayesian test with robust correction.

(G) Cumulative distribution of the difference in $\log_2$ fold changes of completion scores on the basis of 4sU-seq experiments.

See also Figure S2 and Table S1.

CellPress
OPEN ACCESS

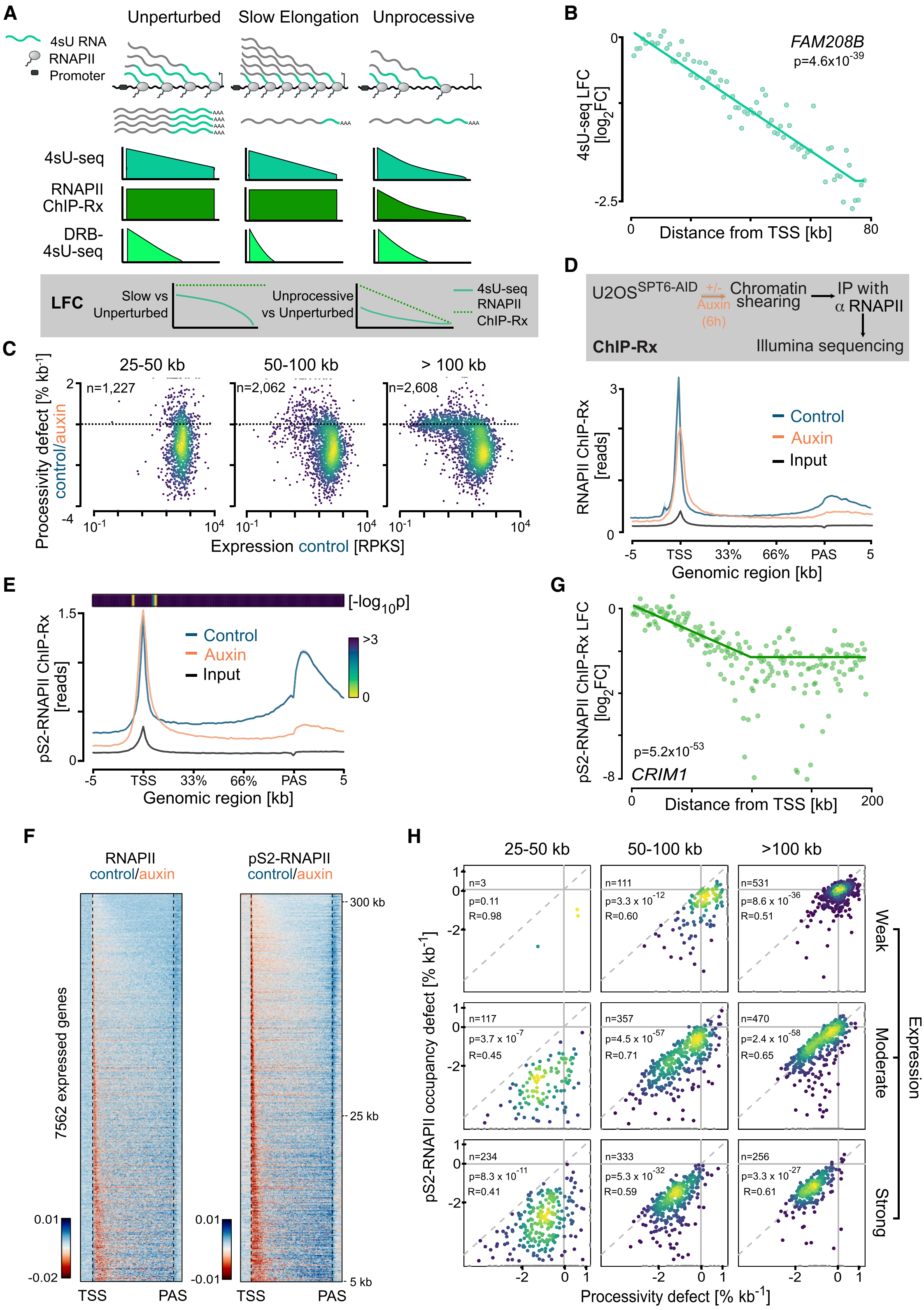

(legend on next page)

Acute SPT6 depletion slows transcription elongation

Our analyses so far do not rule out that the observed drop in 4sU read density along gene bodies was, in part, a consequence of reduced RNAPII elongation rates. Thus, we did DRB-4sU-seq experiments to directly measure RNAPII elongation rates in SPT6-depleted and control cells. In these experiments, RNAPII molecules were reversibly blocked at the pause-release step by adding 5,6-dichloro-1-beta-D-ribofuranosylbenzimidazole (DRB) for 2.5 h and, after DRB washout, allowed to restart transcription in the presence of 4sU (Figure 4A). We sequenced 4sU-labeled transcripts in U2OS$^{SPT6\text{-}AID\text{-}C1}$ cells after 10, 20, or 30 min of washout. In control cells, the 4sU coverage "wavefront" progressed with typical elongation rates of ~3 kb/min (Baluapuri et al., 2019), both in individual genes (Figures 4B, S4A, and S4B) and overall, as shown by metagene and heatmap analyses (Figures 4C and S4C). Furthermore, the 4sU read coverage in control cells had a typical triangular shape (Fuchs et al., 2014). Strikingly, however, for SPT6-depleted cells the read coverage did not drop linearly but instead exhibited a curved shape (Figures 4B and 4C). These observations indicate that the DRB-4sU-seq data are affected by the same phenomenon as the 4sU-seq data, providing additional evidence for a processivity defect upon SPT6 depletion.

Computational tools for estimating wavefront positions (e.g., Zhang et al., 2016) use *ad hoc* approaches that, we noticed, were biased by the apparent processivity defect. This prompted us to develop a statistically principled approach to infer elongation rates using negative binomial regression to model the processivity defect. An exponentially declining function was better suited than a linear function to fit data from auxin-treated cells (3,242 genes; 95% CI, ±500 nt/min; Figures 4D and S4D). All three DRB chase experiments demonstrated that elongation rates were on average reduced by 25% upon acute SPT6 depletion (Figure 4E). In conclusion, DRB-4sU-seq data indicate that processivity defects in SPT6-depleted cells are accompanied by decreased elongation rates.

Elongation defects caused by SPT6 depletion reduce productive transcription

Productive gene transcription is determined by the successful initiation rate of RNAPII (Gressel et al., 2019) but also depends on successful completion of elongation and 3′ end processing. Thus, we analyzed whether the processivity defects in SPT6-depleted cells change the expression of protein-coding genes, using SLAM-seq (Herzog et al., 2017), which quantifies recently synthesized mRNA within the total RNA pool. U2OS$^{SPT6\text{-}AID\text{-}C1}$ cells were labeled with 4sU for 0, 1, 2, and 4 h (Figure 4F), total RNA was alkylated to convert 4sU into cytosine, and completely synthesized RNAs were analyzed using 3′ end sequencing (Figure S4E). The T-to-C mismatches in reads increased over 4 h of labeling (Figures 4G and 4H), indicating the accumulation of new mature mRNA. However, this increase was lower in auxin-treated cells, indicating decreased synthesis of mature RNA (Figure 4H).

We next used the GRAND-SLAM algorithm (Jürges et al., 2018) to obtain absolute estimates of newly synthesized and pre-existing RNA levels and developed a kinetic model to estimate the net synthesis rates and RNA half-lives in control and auxin-treated cells (Figure 4I). Modeling revealed that net synthesis rates in SPT6-depleted cells were reduced 8-fold on average (Figures 4J and S4F). To test whether a stronger processivity defect results in a stronger reduction in net synthesis rate, we defined equivalent sets of genes with no, weak, moderate, or strong processivity defects. We observed a modest but significant difference in net synthesis rates over the four gene sets ($p = 6.3 \times 10^{-5}$, Kruskal-Wallis test; Figure 4K) and concluded that the RNAPII processivity defect is at least in part responsible for reduced synthesis rates in SPT6-depleted cells.

Sustained depletion of SPT6 induces cryptic transcription

Expression of hypomorphic Spt6 in yeast re-distributed nucleosomes and provoked intragenic RNAPII initiation (Doris et al.,

Figure 3. Transcription elongation defects result from processivity loss due to SPT6 depletion

(A) RNAPII activity simulation. Schematic (top) of RNAPII transcribing in an unperturbed, slowly, or unprocessive manner. Regions of transcripts labeled with 4sU during the experimental pulse are marked in green. Simulated RNAPII activity on an 80 kb gene, as observed with 4sU-seq, RNAPII ChIP-Rx, and DRB-4sU-seq methods. Local fold change (LFC) profiles from simulated 4sU-seq and ChIP-Rx in slow versus unperturbed conditions (bottom, left) and unprocessive versus unperturbed conditions (bottom, right).

(B) LFC plot for example gene *FAM208B*. The plot shows $\log_2$ fold changes in read counts in non-overlapping 1 kb windows for the pooled 4sU-seq replicates in U2OS$^{SPT6\text{-}AID\text{-}C1}$ cells in the presence versus absence of auxin.

(C) Scatterplots comparing expression levels in the absence of auxin with processivity defects for expressed genes with an accurate LFC fit, according to gene length. Processivity defect is the rate of the exponential decline in 4sU signal along the gene body for auxin-treated samples compared with control samples. RPKS, reads/kilobase after spike normalization.

(D) Schematic and metagene plot of total-RNAPII ChIP-Rx experiments. Metagene plot shows the read distribution over the gene body averaged overexpressed genes in U2OS$^{SPT6\text{-}AID\text{-}C1}$ cells. Shadows around curves indicate SEM.

(E) Metagene plot of pS2-RNAPII ChIP-Rx experiments. The plot shows the distribution of read density over the gene body overexpressed genes in U2OS$^{SPT6\text{-}AID\text{-}C1}$ cells. p values (two-sided Wilcoxon test) for the difference (auxin/control), calculated from the density values of individual genes at each genomic location, are shown in a heatmap. Shadows around curves indicate SEM.

(F) Relative and scaled heatmaps of pS2- or total-RNAPII ChIP-Rx experiments in U2OS$^{SPT6\text{-}AID\text{-}C1}$ cells. Shown are Z scores calculated from normalized $\log_2$ fold changes between reads from control and auxin-treated conditions. Orange indicates fewer reads and blue indicates more reads in control condition.

(G) LFC plot for example gene *CRIM1.* The plot shows $\log_2$ fold changes in read counts in non-overlapping 1 kb windows for pS2-RNAPII ChIP-Rx experiment in U2OS$^{SPT6\text{-}AID\text{-}C1}$ cells with or without auxin. p value (likelihood ratio test) calculated from the values of the individual bins.

(H) Scatterplots correlating processivity defects with pS2-RNAPII occupancy for genes with accurate LFC fits for the 4sU and ChIP-Rx data. Processivity defect is the rate of exponential decline in 4sU signal along the gene body for auxin-treated cells compared with control cells. The genes are stratified by expression level and length. Pearson's correlation, t test.

See also Figure S3.

CellPress
OPEN ACCESS

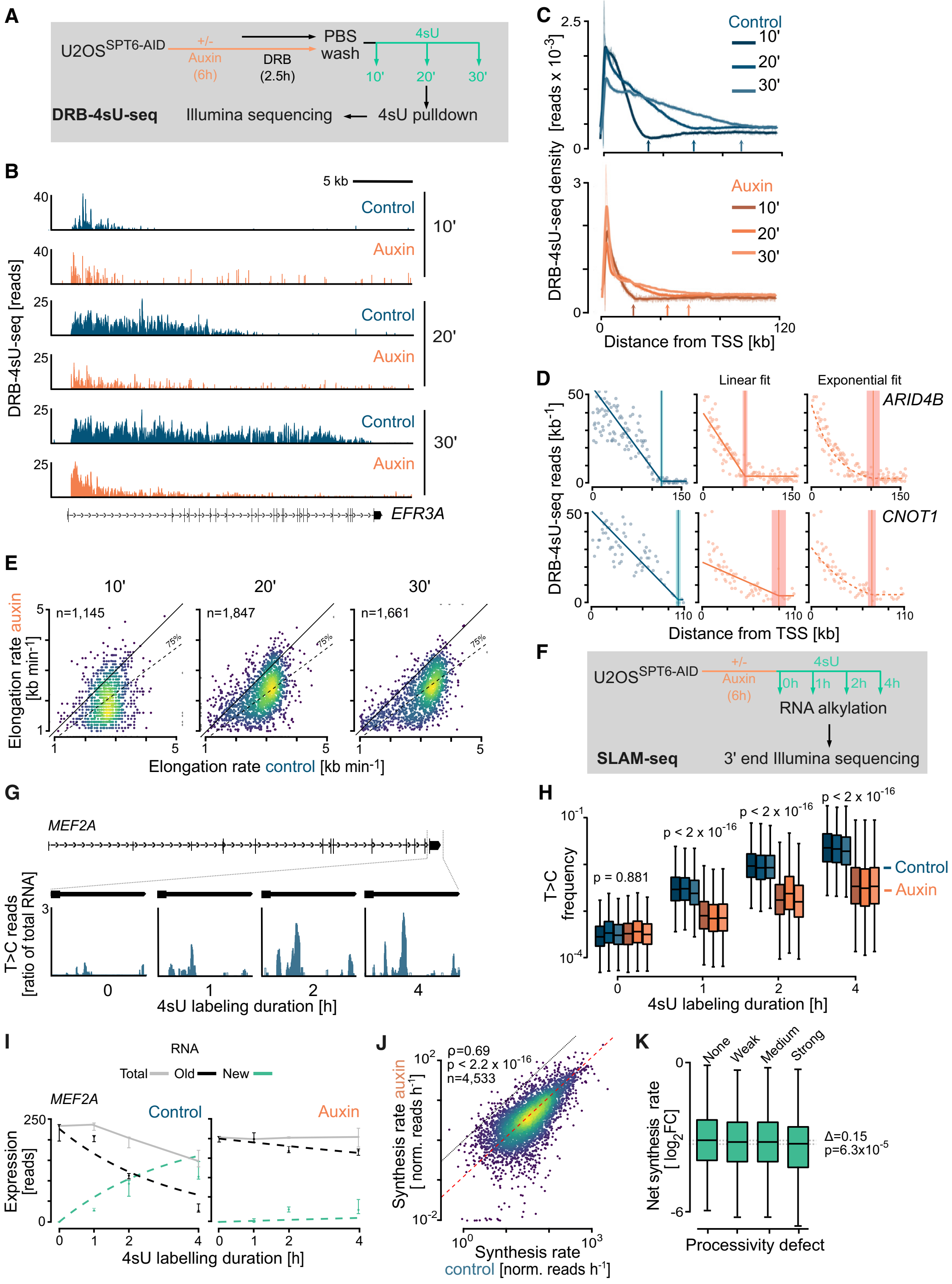

Figure 4. Acute SPT6 depletion slows transcription elongation

(A) Schematic of DRB-4sU-seq experiments.

(B) Browser tracks of the DRB-4sU-seq experiment at *EFR3A* gene in U2OS$^{SPT6\text{-}AID\text{-}C1}$ cells in the presence or absence of auxin, followed by DRB inhibition and release for 10, 20, and 30 min.

(legend continued on next page)

2018). However, when we inspect 4sU-seq profiles from cells with acute SPT6-depletion, we did not see intragenic peaks indicative of cryptic transcription initiation (Figure 5A). To determine if long-term SPT6 depletion is required for intragenic transcription initiation, we silenced SPT6 expression by incubating U2OS cells with a small interfering RNA (siRNA) for 48 h (Figure 5B). 4sU-seq data from this experiment revealed that sustained SPT6 depletion induced a massive redistribution of reads (Figure 5C): instead of an even distribution, the reads clustered in peaks, each a few hundred base pairs wide. Using peak-calling algorithms, we identified 21,317 peaks ($p < 10^{-4}$) in SPT6-depleted cells but only 3,280 peaks in siCTR-treated cells (Figure 5D; Figure S5A). As noted earlier in yeast (Doris et al., 2018), we found various consensus motifs of regulatory transcription factors, such as E-box elements and motifs indicative of core promoter regions (Figure 5E). Overall, 71.3% of the peaks identified in siSPT6 condition contained promoter-associated motifs, compared with only 39.4% in random genomic regions (Figure 5F); a majority of peaks were located in RNAPII-transcribed genes (Figure 5G). Moreover, their appearance correlated with the expression level of the respective genes (Figure S5B). Long-term SPT6 depletion induces the removal of chromatin marks (e.g., H3K36me3), which mark gene bodies (DeGennaro et al., 2013; Nojima et al., 2018). We therefore analyzed chromatin marks in gene bodies that had 4sU peaks upon SPT6 depletion and found a strong association with H3K36me3 (Figure S5C). We concluded that cryptic transcription resulting from sustained SPT6 depletion takes place at specific sites within highly transcribed, H3K36me3-positive genes.

In yeast, this phenomenon has been unequivocally characterized as spurious intragenic initiation (Doris et al., 2018). We therefore used ChIP-Rx of TATA-box binding protein (TBP) to map sites of transcription initiation. Inspection of individual genes (Figure 5H) and heatmap analysis of all expressed genes (Figure 5I) revealed a sharp enrichment of TBP at the annotated TSS in control cells. In contrast, many locations showed increased intragenic TBP signals upon SPT6 sustained depletion (Figures 5H–5J). We concluded that aberrant transcription initiation takes place at cryptic intragenic locations in cells upon long-term SPT6 depletion. To determine whether the observed aberrant transcription also happens upon acute SPT6 depletion, we compared the respective 4sU-seq datasets. Heatmaps centered around all 21,317 peaks appearing upon siRNA-mediated SPT6 depletion showed no sign of enrichment in 4sU reads upon auxin-mediated SPT6 depletion (Figures 5K and S5D). Therefore, in sharp contrast to long-term depletion, acute SPT6 depletion does not induce aberrant intragenic transcription in U2OS cells (Figure S5E).

SPT6 is essential for RNAPII termination and prevents readthrough transcription

Metagene analysis of 4sU-seq data indicated that acute SPT6 depletion resulted not only in a strong elongation defect but also in an increased read level downstream of PAS (Figure 2A). Inspection of individual genes showed a substantial increase in readthrough transcription, with the 4sU signal extending several kilobases after PAS upon SPT6 depletion (Figure 6A). To investigate which genes were affected by this phenomenon, we analyzed read densities in density plots and heatmaps centered on PAS. Strikingly, except for a subset of long genes, we observed a strong global increase in read density downstream of PAS upon SPT6 depletion (Figures 6B and 6C). To quantify readthrough at the gene level, we calculated a readthrough score as the ratio of the 4sU-seq read density after and before PAS (Figure 2D; Table S1) between control and auxin-treated conditions. Overall, 2,419 genes had increased readthrough ($q < 0.05$), while only 273 had decreased readthrough in auxin-treated cells (Figure 6D). qPCR on total RNA confirmed a significant increase in the signal downstream of PAS in SPT6-depleted cells (Figure 6E).

Upon recognition of the poly(A) signal, RNAPII decelerates and continues transcribing beyond PAS until the 5′-3′ exonuclease XRN2 degrades the cleaved RNAPII-tethered nascent transcript and catches up to RNAPII, evicting it from DNA (Cortazar et al., 2019; Parua et al., 2018, 2020). The observed increase in 4sU-labeled reads downstream of PAS suggests that either transcription termination or degradation of the post-PAS transcript is impaired upon acute SPT6 depletion. To clarify this, we analyzed RNAPII ChIP-Rx data around PAS. Strikingly, in SPT6-depleted cells, the usual accumulation of pS2-RNAPII after PAS was

(C) Spline-smoothed averaged metagene plots of DRB-4sU-seq showing the distribution of read density over the gene body from TSS to 120 kb downstream in U2OS$^{SPT6-AID-C1}$ cells. Shadows around curves indicate SEM. Arrows indicate visual wavefront positions, where signal hits background levels.

(D) Estimation of transcription elongation rates using negative binomial regression on DRB-4sU-seq data for *ARID4B* and *CNOT1* genes. Left: linear fit (blue lines) for reads counts in non-overlapping 1 kb windows (blue dots) in the 30 min DRB-4sU-seq experiment in control cells. Middle: linear fit (orange lines) for the same experiment in the presence of auxin (6 h), showing underestimation (*ARID4B*) or overestimation (*CNOT1*). Right: exponential decline fit (orange curve) for the same data. The estimated wavefront (vertical lines) and 95% CI (shaded areas) are indicated.

(E) Scatterplots comparing elongation rates between control and auxin-treated U2OS$^{SPT6-AID-C1}$ cells. Elongation rates were calculated by DRB-4sU-seq followed by DRB inhibition and release after 10, 20, or 30 min.

(F) Schematic of SLAM-seq experiments.

(G) Browser track of SLAM-seq experiments showing the ratio of reads with T-to-C conversion to total RNA over time at *MEF2A* gene in control cells.

(H) T-to-C conversion frequency over time in SLAM-seq experiments in U2OS$^{SPT6-AID-C1}$ cells with or without auxin, followed by 4sU labeling. p values (Wald test) calculated from the values of the individual genes, across the triplicates.

(I) Expression (size-normalized read counts) of example gene *MEF2A* in a SLAM-seq experiment in U2OS$^{SPT6-AID-C1}$ cells. Dots are medians of triplicate samples; error bars represent ranges. Dashed lines show estimated kinetics over time by mathematical modeling.

(J) Scatterplot comparing net transcription rates estimated from SLAM-seq experiments in U2OS$^{SPT6-AID-C1}$ cells treated or not with auxin. Spearman's correlation, asymptotic t test.

(K) Log_2 fold changes in net synthesis rate estimated from SLAM-seq experiments between control and auxin-treated U2OS$^{SPT6-AID-C1}$ cells, according to category of processivity defect for genes divided into equal-sized bins. Kruskal-Wallis test.

See also Figure S4.

CellPress
OPEN ACCESS

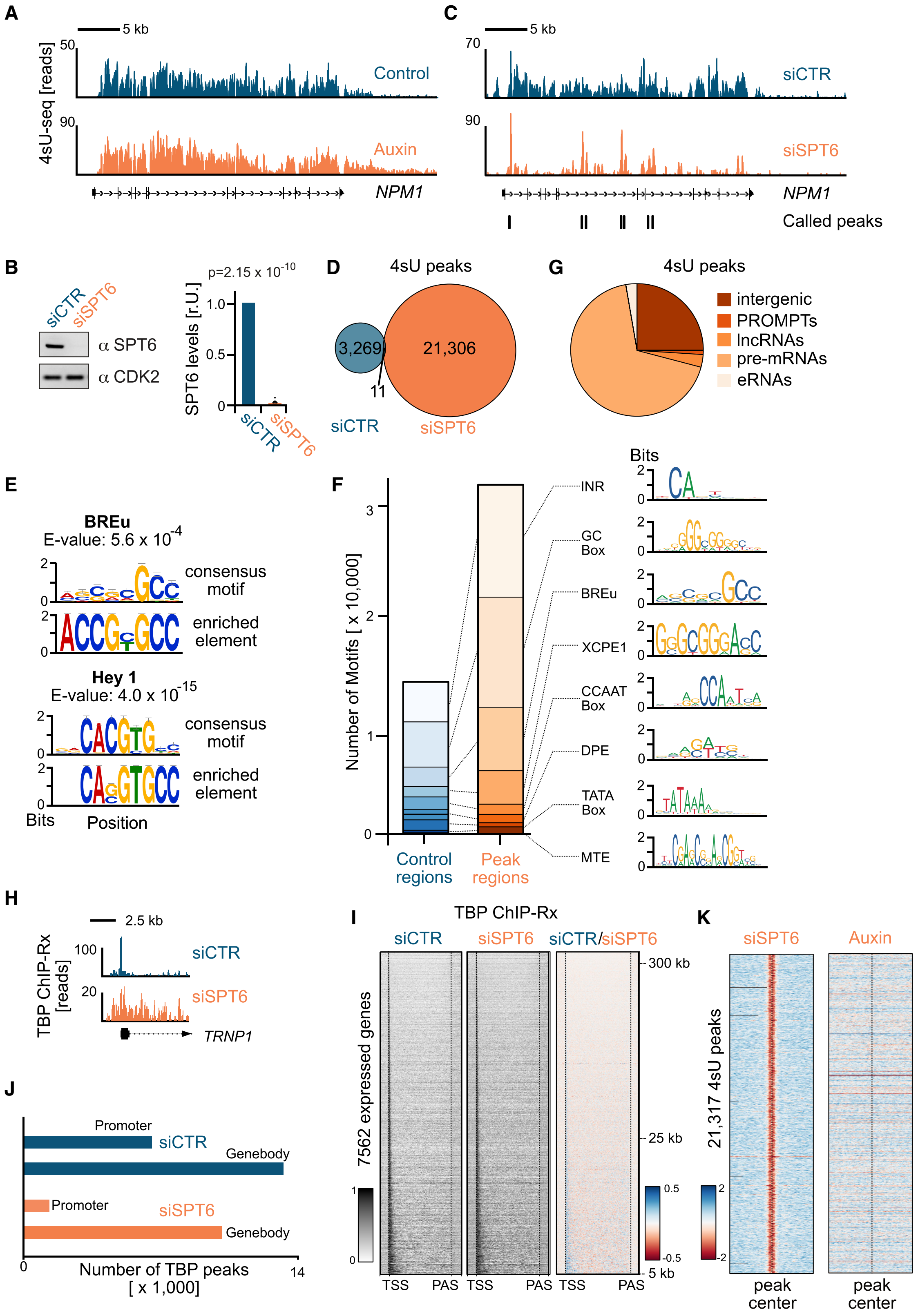

(legend on next page)

almost completely abolished (Figures 6F–6H). Instead, the ChIP-Rx signal remained constant in the termination zone, such that ~5 kb after PAS, it was substantially higher than that seen in control cells, supporting the model of increased readthrough transcription without SPT6. In fact, a readthrough score calculated from pS2-RNAPII ChIP-Rx data showed increased readthrough at 69% of all genes (<25 kb) in SPT6-depleted cells (Figure 6I). We confirmed this observation in SPT6-depleted cells by analyzing (1) pS2-ChIP-Rx data from $U2OS^{SPT6\text{-}AID\text{-}C2}$ (Figures 6G–6I), (2) occupancy of pS2-RNAPII before and after PAS by ChIP-qPCR (Figure S6A), and (3) ChIP-Rx data of total-RNAPII in both clones (Figures S6B–S6D). Thus, we concluded that the increase in 4sU-labeled RNA after PAS in SPT6-depleted cells is due to impaired transcription termination.

We next modeled the kinetic behavior of RNAPII downstream of PAS. We analyzed 4sU read counts in non-overlapping 1 kb windows downstream of PAS and observed a continuous exponential decline in both control and SPT6-depleted cells (Figure S6E). An exponential decline in 4sU signal indicates that transcription downstream of PAS is terminated stochastically at a constant, gene-specific rate. To assess the overall termination defect in SPT6-depleted cells on all genes, we estimated this post-PAS decline rate by negative binomial regression. In control cells, the median rate was 20.0%/kb. Upon SPT6 depletion, the rates were much less (median decline 10.1%/kb). Interestingly, the rates in SPT6-depleted cells correlated strongly with those in control cells (Spearman's $\rho = 0.8$, $p < 2.2 \times 10^{-16}$; Figure 6J). Thus, the number of nucleotides transcribed by RNAPII after mRNA cleavage and before termination roughly doubles for all genes upon acute SPT6 depletion. This result provides evidence that SPT6 affects one of several independent parameters that contribute to the gene-specific rate of transcription termination.

SPT6 is essential for recruiting termination factors and preventing replication stress

As a role for SPT6 in transcription termination of protein-coding genes has not yet been described, we investigated the underlying mechanism by determining if SPT6 depletion affects interactions between RNAPII and other proteins such as termination factors. To this end, we stably expressed HA-tagged RPB3 in $U2OS^{SPT6\text{-}AID\text{-}C1}$ (Figure S7A), isolated chromatin-associated RNAPII complexes, and identified RNAPII-associated proteins using quantitative mass spectrometry (Figure 7A). Analyses of biological triplicates revealed that acute SPT6 depletion reduced the association with RNAPII for 16 RNAPII-interacting proteins ($\log_2 FC < -0.5$, $p < 0.05$; Table S3; Figure 7B). Notably, all identified proteins of the cleavage stimulation factor (CSTF1, CSTF2, CSTF3) and other termination factors such as symplekin and the exonuclease XRN2 lost their association to RNAPII upon acute SPT6 depletion, while their cellular levels were unchanged (Figure S7B). Intriguingly, ChIP-Rx experiment for CSTF2 showed a strong enrichment at PAS in control cells, which largely disappeared upon acute SPT6 depletion (Figures 7C and 7D). We concluded that SPT6 augments the association of a specific set of termination factors with RNAPII and chromatin.

The drastic changes in RNAPII activity induced by acute SPT6 depletion could have immediate cellular consequences. To investigate this possibility, we first analyzed cell growth and noticed that auxin treatment caused a massive drop in viable $U2OS^{SPT6\text{-}AID\text{-}C1}$ cell count (Figure S7C). Moreover, annexin V-PI experiments demonstrated a large increase of apoptotic cells 24 h after auxin addition in $U2OS^{SPT6\text{-}AID\text{-}C1}$ but not in control cells (Figure S7D). We concluded that apoptosis is responsible for the cell viability decline after SPT6 depletion.

siRNA-mediated depletion of SPT6 is accompanied by increased levels of R-loops (Nojima et al., 2018), which are complexes of DNA-RNA hybrids and single-stranded DNA that can cause replication stress. As termination defects are believed to cause R-loops (Skourti-Stathaki and Proudfoot, 2014), we analyzed if acute SPT6 depletion also resulted in replication stress. To this end, we measured levels of phospho-RPA2 and γH2AX, two proteins phosphorylated upon replication stress and DNA damage. Strikingly, immunoblots demonstrated enhanced levels already 12 h after auxin addition (Figure 7E). ChIP-Rx experiments for γH2AX and H3 in U2OS cells showed an enhanced signal of γH2AX after PAS in SPT6-depleted cells (Figures 7F and S7E–S7G). We concluded that acute SPT6

Figure 5. Sustained but not acute SPT6 depletion induces cryptic transcription

(A) Browser tracks showing a 4sU-seq experiment at *NPM1* gene in control and auxin-treated $U2OS^{SPT6\text{-}AID\text{-}C1}$ cells.

(B) Immunoblot and quantification of SPT6. $U2OS^{WT}$ cells were treated with SPT6-specific siRNA (siSPT6) or non-targeting siCTR (48 h). CDK2, loading control. Quantification of SPT6 immunoblots from two biological replicates loaded three times each. Values are mean ± SD. p values (two-tailed unpaired t test) calculated from the values of the individual normalized intensities.

(C) Browser tracks showing a 4sU-seq experiment at *NPM1* gene in $U2OS^{WT}$ cells treated with siSPT6 or siCTR (48 h). Peaks called by MACS2 are shown below.

(D) Venn diagram of peaks called by MACS2 in a 4sU-seq experiment on $U2OS^{WT}$ cells (replicate 1) after treatment with siSPT6 or siCTR (48 h).

(E) Sequence logos of elements enriched in 4sU peaks. Selected examples of motifs found to be enriched by MEME-ChIP in the peaks called in siSPT6-treated cells. The consensus motif in the database (top) is compared with the enriched sequence element (bottom).

(F) Sequence logos and stacked bar plots indicating the occurrences of motif elements in the JASPAR POLII database for peaks originating from siSPT6 condition and random genomic locations of the same size.

(G) Genomic locations of the peaks originating from siSPT6 conditions. eRNA, enhancer RNA; lncRNA, long intergenic non-coding RNA; PROMPT, promoter upstream transcripts.

(H) Browser tracks showing a TATA binding protein (TBP) ChIP-Rx experiment at *TRNP1* gene in $U2OS^{WT}$ cells treated with siSPT6 or siCTR (48 h).

(I) Heatmaps showing *Z* scores calculated from input-normalized TBP ChIP-Rx reads in $U2OS^{WT}$ cells treated with siCTR (left) or siSPT6 (middle), and relative heatmap (right) for expressed genes (>5 kb), sorted by length.

(J) TBP ChIP-Rx peaks originating from U2OS cells treated with siCTR or siSPT6 conditions between promoter and gene body regions.

(K) Relative heatmaps centered at peaks (±2.5 kb) found in siSPT6 condition. The left heatmap shows *Z* scores comparing 4sU-seq reads from siSPT6-treated and siCTR-treated cells (48 h). The right heatmap shows *Z* scores comparing 4sU reads from auxin-treated (6 h) and control $U2OS^{SPT6\text{-}AID\text{-}C1}$ cells.

See also Figure S5.

CellPress
OPEN ACCESS

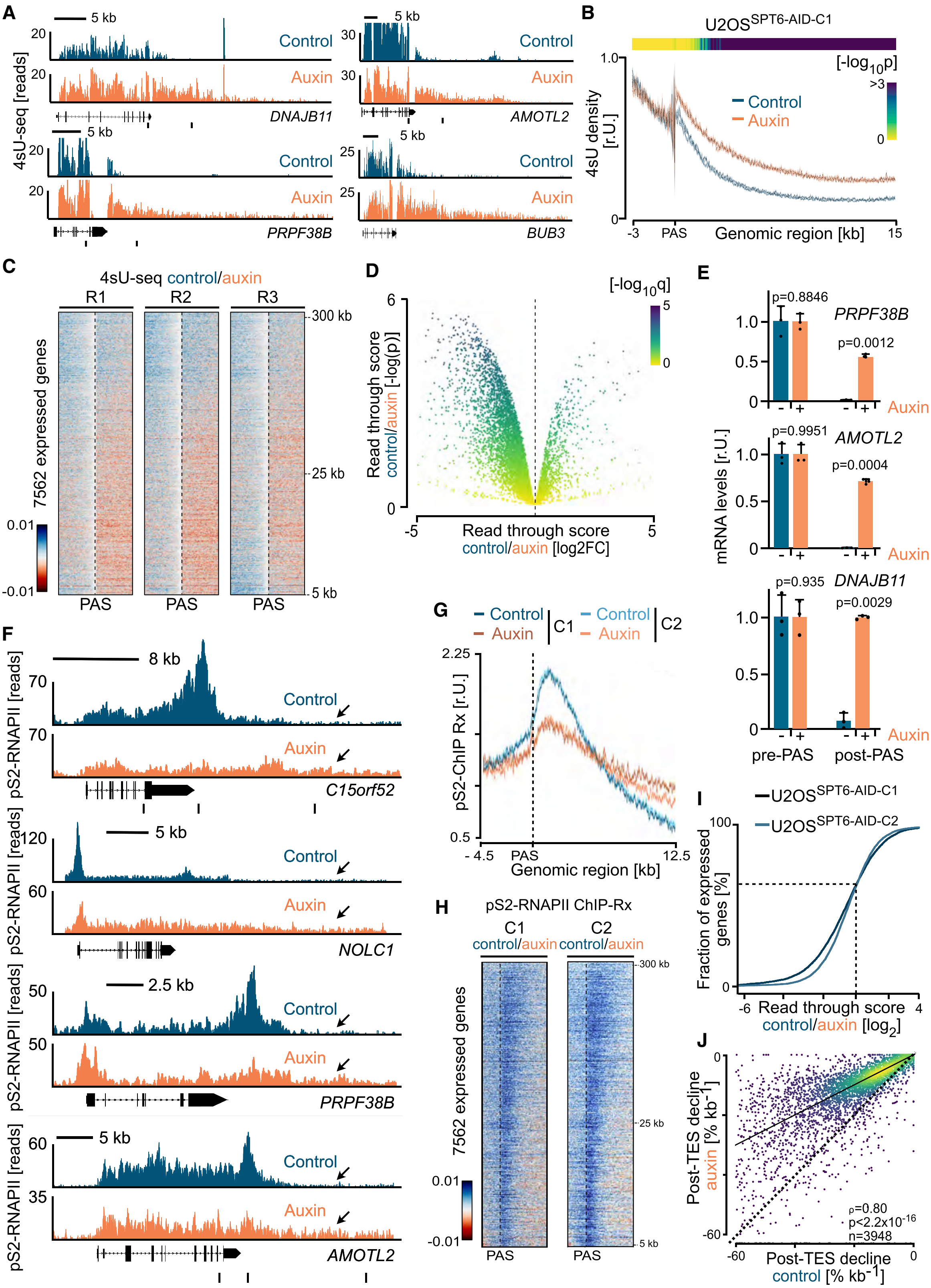

(legend on next page)

depletion rapidly activates stress response pathways (Hamperl et al., 2017) and hypothesized that this activation originates from conflicts of the replication machinery with transcriptionally engaged RNAPII around PAS.

To test this hypothesis, we did proximity ligation assays to probe for co-occurrences of RNAPII with different phosphorylation marks and γH2AX. Upon SPT6 depletion, we observed an increase in proximity pairs per nucleus for γH2AX and pS2-, pY1-, and pT4-RNAPII (Figures 7G and 7H) but not for pS5- or unphosphorylated RNAPII (Figure S7H). Furthermore, we found a 5.1-fold increase in proximity pairs with antibodies recognizing γH2AX and R-loops in SPT6-depleted cells (Figure S7I). Strikingly, addition of auxin completely blocked BrdU incorporation in U2OS$^{SPT6\text{-}AID\text{-}C1}$ cells (Figures 7I and S7J), indicating impaired DNA replication and S-phase arrest in SPT6-depleted cells. Together, these experiments indicate a higher incidence of conflicts between the replication machinery and non-terminating RNAPII upon SPT6 depletion.

DISCUSSION

With our system for the rapid, inducible degradation of SPT6, we estimated the direct effects of SPT6 depletion on the kinetic activity of RNAPII by combining four genomic approaches (4sU-seq, DRB-4sU-seq, RNAPII ChIP-Rx, and SLAM-seq) with mathematic modeling. Overall, we observed a drastic loss of RNAPII processivity accompanied by reduced RNAPII elongation rates. Importantly, this elongation defect was prevalent at all protein-coding genes and not restricted to certain regions of the gene body. This successive loss of active RNAPII was amplified over the gene length and therefore was most pronounced at long genes, where virtually no RNAPII molecules reached PAS without SPT6 (Figure 7J). Our findings define SPT6 as an essential transcription elongation factor having a pivotal function in gene expression in mammalian cells. In addition, we made two other discoveries.

First, we observed that sustained depletion of SPT6 induced transcription defects distinct from those of acute depletion. Sustained SPT6 depletion caused the onset of peak-like densities of 4sU-labeled transcripts inside genes. This phenotype of discontinuous intragenic transcription was first observed in yeast expressing the *Spt6-1004* mutant (Kaplan et al., 2003). Further characterization unequivocally demonstrated that yeast expressing this hypomorphic Spt6 mutant exhibit spurious intragenic initiation of RNAPII (Doris et al., 2018). Correspondingly, we observed a global shift of TBP binding from canonical protomers toward intragenic locations upon long-term SPT6 depletion. Interestingly, we did not observe signs of intragenic initiation upon acute SPT6 depletion.

From these observations, we propose that SPT6 has a dual effect on RNAPII transcription (Figure S5E): On one hand, SPT6 primarily promotes RNAPII elongation. It is plausible that SPT6 aids the passage of RNAPII through chromatinized DNA via its ability to bind histones (Jeronimo and Robert, 2016) and that loss of SPT6 reduces the likelihood that RNAPII successfully breaks the nucleosomal barrier (Teves et al., 2014), thus reducing RNAPII processivity. It is also possible that SPT6 promotes elongation via histone-independent, direct allosteric effects on RNAPII (Vos et al., 2018). On the other hand, SPT6 prevents spurious intragenic initiation of RNAPII (Figure S5E), which occurs only during sustained depletion of SPT6. We therefore hypothesize that this phenotype is a result of the eventual loss of chromatin organization, upon repeated passage of RNAPII machinery without the histone chaperone function of SPT6. Consequently, nucleosomes in intragenic regions are decorated with incorrect histone modifications or depleted from chromatin, which in turn induces spurious intragenic initiation of RNAPII. This model is supported by the observation that these intragenic peaks occur most prominently in highly expressed genes and agrees with evidence that long-term SPT6 depletion induces the loss and improper exchange of nucleosomes in bodies of highly expressed genes (Ivanovska et al., 2011; Perales et al., 2013).

The second unexpected finding of our work is that acute SPT6 depletion resulted in termination defects and drastically extended readthrough transcription at thousands of genes. This termination defect was most pronounced at short genes,

Figure 6. Acute SPT6 depletion causes readthrough at protein-coding genes

(A) Browser tracks showing 4sU-seq experiments for four genes in U2OS$^{SPT6\text{-}AID\text{-}C1}$ cells. Primers for qPCR are indicated below.

(B) Metagene plots showing distribution of read density positioned around PAS, averaged and normalized for reads 3 kb upstream and displaying the reads 15 kb downstream in U2OS$^{SPT6\text{-}AID\text{-}C1}$ cells from 4sU-seq experiments. Shadows around curves indicate SEM. p values (one-sided Wilcoxon test) are calculated from the density values of the individual genes across individual bins and shown as a heatmap.

(C) Relative heatmaps for three replicates showing *Z* scores calculated from $\log_2$ fold changes between normalized reads from U2OS$^{SPT6\text{-}AID\text{-}C1}$ cells from 4sU-seq for expressed genes, sorted by length. Heatmaps are centered at PAS (±5 kb). Orange indicates less reads and blue indicates more reads in control cells.

(D) Volcano plot comparing $\log_2$ fold changes and the statistical significance of readthrough scores calculated in control and auxin conditions for expressed genes. The color of individual data points indicates the corresponding adjusted p value.

(E) qPCR analysis of readthrough transcription for three genes (primer pairs indicated in A), relative to PAS upstream primers. Values are mean ± SD (n = 3). Two-tailed unpaired t test assuming equal variance.

(F) Browser tracks showing pS2-RNAPII ChIP-Rx experiments for selected genes in U2OS$^{SPT6\text{-}AID\text{-}C1}$ cells. Primers for qPCR are indicated below.

(G) Metagene plots showing distribution of read density from pS2-RNAPII ChIP-Rx positioned around PAS, averaged for 4.5 kb upstream and 12.5 kb downstream in U2OS$^{SPT6\text{-}AID\text{-}C1}$ and U2OS$^{SPT6\text{-}AID\text{-}C2}$ cells. Shadows around curves indicate SEM.

(H) Relative heatmaps for read density from pS2-RNAPII ChIP-Rx. Shown are *Z* scores calculated from $\log_2$ fold changes between spike-normalized reads from control and auxin-treated U2OS$^{SPT6\text{-}AID\text{-}C1}$ and U2OS$^{SPT6\text{-}AID\text{-}C2}$ cells for expressed genes, sorted by length. Heatmaps are positioned around PAS, showing 4.5 kb upstream and 12.5 kb downstream regions.

(I) Cumulative distribution of the difference in $\log_2$ fold changes of readthrough scores on the basis of pS2-RNAPII ChIP-Rx from control and auxin-treated cells.

(J) Post-PAS decline rates for transcription termination for genes in the pooled 4sU-seq experiment in U2OS$^{SPT6\text{-}AID\text{-}C1}$ cells in the presence or absence of auxin. Spearman's correlation, asymptotic t test.

See also Figure S6 and Table S1.

CellPress
OPEN ACCESS

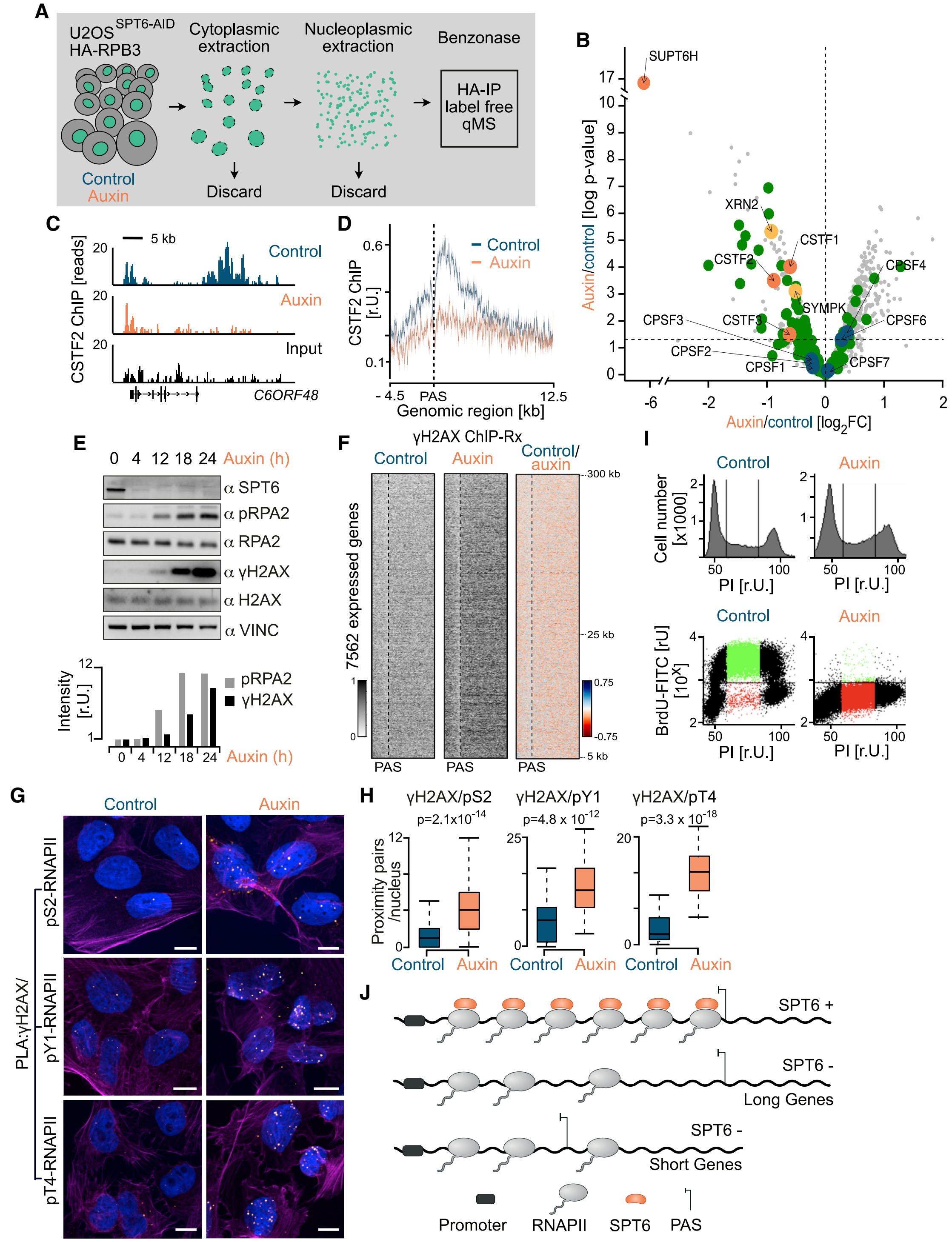

Figure 7. SPT6 is essential for the recruitment of termination factors and prevents replication stress

(A) Schematic of quantitative mass spectrometry for identifying RNAPII-associated proteins.

(B) Volcano plot showing proteins whose association with RNAPII changed in response to SPT6 depletion in U2OS$^{SPT6\text{-}AID\text{-}C1}$ cells treated with auxin. Negative $\log_2$ fold change values indicate the protein requires SPT6 to associate with RNAPII. RNAPII-interacting proteins are shown as green. Selected termination factors are labeled.

(C) Browser tracks showing CSTF2 ChIP-Rx experiments for *C6ORF48* gene in U2OS$^{SPT6\text{-}AID\text{-}C1}$ cells with or without auxin.

(legend continued on next page)

as it was masked by the processivity defect at longer genes (Figure 7J). A role for SPT6 on RNAPII termination at protein-coding genes was so far unknown, although siRNA-mediated depletion of SPT6 had been shown to induce termination defects at non-coding RNAs (Nojima et al., 2018). Our analysis of RNAPII-interacting proteins indicates that SPT6 supports the association of the transcription machinery with termination factors such as XRN2, symplekin, and the CSTF complex. Intriguingly, depletion of components of the CSTF complex resulted in transcription readthrough, similar to our observation in SPT6-deficient cells (Nojima et al., 2015). There is accumulating evidence that termination is a highly regulated step in the transcription cycle. For example, readthrough transcription can be induced by environmental stimuli such as viral infections (Rutkowski et al., 2015) and osmotic exposure (Vilborg et al., 2015).

A large fraction of cells became apoptotic shortly after acute SPT6 depletion, indicating that this cellular response is a direct consequence of immediate transcription defects. This could relate to previous observations that siRNA-mediated depletion of SPT6 induces R-loops (Nojima et al., 2018). Readthrough transcription increases R-loop levels (Castillo-Guzman et al., 2020) and transcription-replication conflicts, resulting in rapid dell death (Morales et al., 2016). In agreement with these results, we found signs of replication stress in cells acutely depleted of SPT6, with increased co-occurrences of R-loop and γH2AX after PAS.

Limitations of the study

This study is based on auxin- and siRNA-mediated depletion of SPT6 in a human osteosarcoma cell line. It is possible that SPT6 functions differently in other cells or organisms. However, the effects of long-term SPT6 depletion in our study agree with observations in yeast (Doris et al., 2018) and human HeLa cells (Nojima et al., 2018), suggesting that SPT6 functions are conserved in eukaryotes.

One important conclusion of our study is that SPT6 prevents readthrough transcription at protein-coding genes transcribed by RNAPII, as we detected increased RNAPII ChIP-Rx signals and more 4sU-labeled RNAs after the normal termination zones upon depletion of SPT6. Although the first effect indicates a termination defect, increased post-PAS RNA levels could also originate from changes in stability of these RNA species. In fact, we observed that the association between RNAPII and the exonuclease XRN2, which is responsible for post-PAS transcript degradation, depends on SPT6. We therefore cannot rule out that SPT6 also regulates post-PAS transcript stability in addition to transcription termination. However, these processes are highly connected, as XRN2 activity is also essential for termination (Fisher, 2017; Fong et al., 2015; West et al., 2004).

We labeled newly synthesized RNA by a 15 min pulse with 4sU. During this time, RNAPII molecules transcribe about 45 kb. As such, not all 4sU-labeled RNAs can be considered nascent. Thus, analysis of 4sU-seq data alone does not allow deducing the position of RNAPII during cell harvest. Therefore, we combined the analysis of 4sU-seq data with analyses of DRB-4sU-seq, RNAPII ChIP-Rx, and SLAM-seq data and furthermore included mathematical modeling, which all together enabled us to study how SPT6 affects the kinetic behavior of RNAPII during elongation and termination. Because of the limited resolution of RNAPII ChIP-Rx experiments, we did not investigate the role of SPT6 during and immediately after pausing.

Although our data demonstrate that SPT6 suppresses chromatin marks indicative of DNA damage and replication stress in and after the termination zone, they do not prove that the role of SPT6 in transcription elongation and termination is the sole source of the phenotypes observed (S-phase arrest, apoptosis). Other pathways, such as the upregulation of *BRCA1* by SPT6 in glioblastoma cells (Obara et al., 2020), could contribute to the suppression of replication stress. The acute cellular depletion system for SPT6 developed here is a versatile tool for further exploring the role of SPT6 in the suppression of replication stress at a mechanistic level.

STAR★METHODS

Detailed methods are provided in the online version of this paper and include the following:

- KEY RESOURCES TABLE
- RESOURCE AVAILABILITY
  - Lead contact
  - Materials availability
  - Data and code availability
- EXPERIMENTAL MODEL AND SUBJECT DETAILS
  - Cell culture and auxin treatment
  - Endogenous knock-in of AID tag and cell line transfection

(D) Metagene plots showing distribution of read density from CSTF2 ChIP-Rx positioned around PAS, averaged for 4.5 kb upstream and 12.5 kb downstream in U2OS$^{SPT6\text{-}AID\text{-}C1}$ cells. Shadows around curves indicate SEM.
(E) Top: immunoblots of U2OS$^{SPT6\text{-}AID\text{-}C1}$ cells treated for the indicated times. Vinculin, loading control. Bottom: quantification of pRPA2 and γH2AX relative to RPA2 and H2AX, respectively, from two replicates.
(F) Absolute and relative heatmaps of reads from γH2AX ChIP-Rx in U2OS$^{SPT6\text{-}AID\text{-}C1}$ cells in the presence or absence of auxin (24 h). Shown are *Z* scores calculated from spike-normalized reads (left) or $\log_2$ fold changes between spike-normalized reads (right) from control and auxin conditions. Heatmaps are positioned around PAS, showing 4.5 kb upstream and 12.5 kb downstream regions.
(G) Immunofluorescence images of proximity ligation assays (PLAs) between RNAPII and γH2AX in U2OS$^{SPT6\text{-}AID\text{-}C1}$ cells. Scale bar: 10 μm.
(H) Quantification of PLA foci per nucleus from the corresponding conditions in (G). p values (one-sided Wilcoxon test) for the likelihood of auxin being more than control.
(I) Cell cycle distribution assay. Cells were treated with auxin (24 h), labeled with BrdU, stained with PI, and analyzed using flow cytometry. The amount of intercalating PI (top) and the correlation of BrdU to PI (bottom) are shown. Cells that are BrdU positive in S phase are marked green, while those that are negative are red.
(J) Model of SPT6-induced RNAPII transcription changes.
See also Figure S7.

CellPress
OPEN ACCESS

- METHOD DETAILS
  - General cloning and plasmid constructs
  - Genomic PCR
  - Immunoblotting
  - Chromatin IP with reference exogenous genome spike-in and deep sequencing (ChIP-Rx)
  - Quantitative PCR
  - 4sU sequencing
  - DRB-4sU sequencing
  - SLAM-seq
  - Cellular growth and apoptosis assays
  - Proximity ligation assay
  - Quantitative mass spectrometry
- QUANTIFICATION AND STATISTICAL ANALYSIS
  - Transcription readthrough score calculation
  - Transcription completion score calculation
  - γH2AX read density comparison
  - Antisense and PROMPT expression analyses
  - Image processing
  - Peak calling and visualization
  - Motif search and enrichment analysis
  - Metagene plots
  - Heatmaps
  - Volcano plots
  - LogFC calculations between auxin-treated and control samples for MS data
  - ODE model of transcription
  - Definition of transcription units
  - LFC regression in gene bodies
  - Negative binomial regression in the termination zone
  - Negative binomial regression for estimating the wave-front in DRB-4sU-seq data
  - Analysis of SLAM-seq data
  - Kinetic modeling of SLAM-seq data

SUPPLEMENTAL INFORMATION

Supplemental information can be found online at https://doi.org/10.1016/j.molcel.2021.06.016.

ACKNOWLEDGMENTS

We thank André Kutschke and Margarete Göbel from the Core Unit Systemmedizin for excellent technical assistance and Fred Winston and the Fragile Nucleosome community for helpful advice. Valerie Matarese provided scientific editing. This work was supported by grants from Joachim Hertz Stiftung (to A.B.), the German Research Foundation (DFG; WO 2108/1-1 and GRK 2243 to P.B. and E.W., ER 927/2-1 to F.E.), German Cancer Aid (MSNZ funding to E.W.), and the European Research Council (Auromyc to M.E. and TarMYC to E.W.).

AUTHOR CONTRIBUTIONS

A.N., P.B., and A.B. conducted and interpreted all experiments. A.B., F.E., and P.B. analyzed and interpreted original and published genomic data. B.A. generated the AID cell line. A.N. characterized all cell clones. A.S. performed mass spectrometry and analyzed the data. S.B. and L.D. provided support for the SLAM-seq experiment. M.E. provided critical reagents, instruments, and advice. F.E. carried out mathematical modeling of genomics data. A.B., F.E., and E.W. conceptualized the project and designed and supervised the experiments. E.W., F.E., P.B., A.N., and A.B. wrote the manuscript with input from all authors.

DECLARATION OF INTERESTS

The authors declare no competing interests.

Received: November 29, 2020
Revised: April 24, 2021
Accepted: June 11, 2021
Published: July 6, 2021

REFERENCES

Amemiya, H.M., Kundaje, A., and Boyle, A.P. (2019). The ENCODE blacklist: identification of problematic regions of the genome. Sci. Rep. *9*, 9354.

Andersson, R., Gebhard, C., Miguel-Escalada, I., Hoof, I., Bornholdt, J., Boyd, M., Chen, Y., Zhao, X., Schmidl, C., Suzuki, T., et al. (2014). An atlas of active enhancers across human cell types and tissues. Nature *507*, 455–461.

Andrulis, E.D., Guzmán, E., Döring, P., Werner, J., and Lis, J.T. (2000). High-resolution localization of Drosophila Spt5 and Spt6 at heat shock genes in vivo: roles in promoter proximal pausing and transcription elongation. Genes Dev. *14*, 2635–2649.

Ardehali, M.B., Yao, J., Adelman, K., Fuda, N.J., Petesch, S.J., Webb, W.W., and Lis, J.T. (2009). Spt6 enhances the elongation rate of RNA polymerase II in vivo. EMBO J. *28*, 1067–1077.

Aygün, O., Svejstrup, J., and Liu, Y. (2008). A RECQ5-RNA polymerase II association identified by targeted proteomic analysis of human chromatin. Proc. Natl. Acad. Sci. U S A *105*, 8580–8584.

Bailey, T.L., Boden, M., Buske, F.A., Frith, M., Grant, C.E., Clementi, L., Ren, J., Li, W.W., and Noble, W.S. (2009). MEME SUITE: tools for motif discovery and searching. Nucleic Acids Res. *37*, W202–W208.

Baluapuri, A., Hofstetter, J., Dudvarski Stankovic, N., Endres, T., Bhandare, P., Vos, S.M., Adhikari, B., Schwarz, J.D., Narain, A., Vogt, M., et al. (2019). MYC recruits SPT5 to RNA polymerase II to promote processive transcription elongation. Mol. Cell *74*, 674–687.e11.

Bortvin, A., and Winston, F. (1996). Evidence that Spt6p controls chromatin structure by a direct interaction with histones. Science *272*, 1473–1476.

Brázda, P., Krejčíková, M., Kasiliauskaite, A., Šmiřáková, E., Klumpler, T., Vácha, R., Kubíček, K., and Štefl, R. (2020). Yeast Spt6 reads multiple phosphorylation patterns of RNA polymerase II C-terminal domain in vitro. J. Mol. Biol. *432*, 4092–4107.

Castillo-Guzman, D., Hartono, S.R., Sanz, L.A., and Chédin, F. (2020). SF3B1-targeted splicing inhibition triggers global alterations in transcriptional dynamics and R-loop metabolism. bioRxiv. https://doi.org/10.1101/2020.06.08.130583.

Chen, F.X., Smith, E.R., and Shilatifard, A. (2018). Born to run: control of transcription elongation by RNA polymerase II. Nat. Rev. Mol. Cell Biol. *19*, 464–478.

Close, D., Johnson, S.J., Sdano, M.A., McDonald, S.M., Robinson, H., Formosa, T., and Hill, C.P. (2011). Crystal structures of the S. cerevisiae Spt6 core and C-terminal tandem SH2 domain. J. Mol. Biol. *408*, 697–713.

Cortazar, M.A., Sheridan, R.M., Erickson, B., Fong, N., Glover-Cutter, K., Brannan, K., and Bentley, D.L. (2019). Control of RNA Pol II speed by PNUTS-PP1 and Spt5 dephosphorylation facilitates termination by a "sitting duck torpedo" mechanism. Mol. Cell *76*, 896–908.e4.

Cox, J., and Mann, M. (2008). MaxQuant enables high peptide identification rates, individualized p.p.b.-range mass accuracies and proteome-wide protein quantification. Nat. Biotechnol. *26*, 1367–1372.

DeGennaro, C.M., Alver, B.H., Marguerat, S., Stepanova, E., Davis, C.P., Bähler, J., Park, P.J., and Winston, F. (2013). Spt6 regulates intragenic and antisense transcription, nucleosome positioning, and histone modifications genome-wide in fission yeast. Mol. Cell. Biol. *33*, 4779–4792.

Dobin, A., Davis, C.A., Schlesinger, F., Drenkow, J., Zaleski, C., Jha, S., Batut, P., Chaisson, M., and Gingeras, T.R. (2013). STAR: ultrafast universal RNA-seq aligner. Bioinformatics *29*, 15–21.

Doris, S.M., Chuang, J., Viktorovskaya, O., Murawska, M., Spatt, D., Churchman, L.S., and Winston, F. (2018). Spt6 is required for the fidelity of promoter selection. Mol. Cell *72*, 687–699.e6.

Dronamraju, R., Kerschner, J.L., Peck, S.A., Hepperla, A.J., Adams, A.T., Hughes, K.D., Aslam, S., Yoblinski, A.R., Davis, I.J., Mosley, A.L., and Strahl, B.D. (2018). Casein kinase II phosphorylation of Spt6 enforces transcriptional fidelity by maintaining Spn1-Spt6 interaction. Cell Rep. *25*, 3476–3489.e5.

Eitoku, M., Sato, L., Senda, T., and Horikoshi, M. (2008). Histone chaperones: 30 years from isolation to elucidation of the mechanisms of nucleosome assembly and disassembly. Cell. Mol. Life Sci. *65*, 414–444.

Endoh, M., Zhu, W., Hasegawa, J., Watanabe, H., Kim, D.K., Aida, M., Inukai, N., Narita, T., Yamada, T., Furuya, A., et al. (2004). Human Spt6 stimulates transcription elongation by RNA polymerase II in vitro. Mol. Cell. Biol. *24*, 3324–3336.

Erhard, F. (2018). Estimating pseudocounts and fold changes for digital expression measurements. Bioinformatics *34*, 4054–4063.

Erhard, F., and Zimmer, R. (2015). Count ratio model reveals bias affecting NGS fold changes. Nucleic Acids Res. *43*, e136.

Farnung, L., Vos, S.M., and Cramer, P. (2018). Structure of transcribing RNA polymerase II-nucleosome complex. Nat. Commun. *9*, 5432.

Fisher, R.P. (2017). CDK regulation of transcription by RNAP II: not over 'til it's over? Transcription *8*, 81–90.

Fong, N., Brannan, K., Erickson, B., Kim, H., Cortazar, M.A., Sheridan, R.M., Nguyen, T., Karp, S., and Bentley, D.L. (2015). Effects of transcription elongation rate and Xrn2 exonuclease activity on RNA polymerase II termination suggest widespread kinetic competition. Mol. Cell *60*, 256–267.

Freese, N.H., Norris, D.C., and Loraine, A.E. (2016). Integrated genome browser: visual analytics platform for genomics. Bioinformatics *32*, 2089–2095.

Fuchs, G., Voichek, Y., Benjamin, S., Gilad, S., Amit, I., and Oren, M. (2014). 4sUDRB-seq: measuring genomewide transcriptional elongation rates and initiation frequencies within cells. Genome Biol. *15*, R69.

Gates, L.A., Foulds, C.E., and O'Malley, B.W. (2017). Histone marks in the 'driver's seat': functional roles in steering the transcription cycle. Trends Biochem. Sci. *42*, 977–989.

Gouot, E., Bhat, W., Rufiange, A., Fournier, E., Paquet, E., and Nourani, A. (2018). Casein kinase 2 mediated phosphorylation of Spt6 modulates histone dynamics and regulates spurious transcription. Nucleic Acids Res. *46*, 7612–7630.

Gressel, S., Schwalb, B., and Cramer, P. (2019). The pause-initiation limit restricts transcription activation in human cells. Nat. Commun. *10*, 3603.

Hainer, S.J., and Martens, J.A. (2016). Regulation of chaperone binding and nucleosome dynamics by key residues within the globular domain of histone H3. Epigenetics Chromatin *9*, 17.

Hamperl, S., Bocek, M.J., Saldivar, J.C., Swigut, T., and Cimprich, K.A. (2017). Transcription-replication conflict orientation modulates R-loop levels and activates distinct DNa damage responses. Cell *170*, 774–786.e19.

Hartzog, G.A., Wada, T., Handa, H., and Winston, F. (1998). Evidence that Spt4, Spt5, and Spt6 control transcription elongation by RNA polymerase II in Saccharomyces cerevisiae. Genes Dev. *12*, 357–369.

Herzog, V.A., Reichholf, B., Neumann, T., Rescheneder, P., Bhat, P., Burkard, T.R., Wlotzka, W., von Haeseler, A., Zuber, J., and Ameres, S.L. (2017). Thiol-linked alkylation of RNA to assess expression dynamics. Nat. Methods *14*, 1198–1204.

Hon, C.C., Ramilowski, J.A., Harshbarger, J., Bertin, N., Rackham, O.J., Gough, J., Denisenko, E., Schmeier, S., Poulsen, T.M., Severin, J., et al. (2017). An atlas of human long non-coding RNAs with accurate 5′ ends. Nature *543*, 199–204.

Ivanovska, I., Jacques, P.E., Rando, O.J., Robert, F., and Winston, F. (2011). Control of chromatin structure by spt6: different consequences in coding and regulatory regions. Mol. Cell. Biol. *31*, 531–541.

Jeronimo, C., and Robert, F. (2016). Histone chaperones FACT and Spt6 prevent histone variants from turning into histone deviants. BioEssays *38*, 420–426.

Jeronimo, C., Watanabe, S., Kaplan, C.D., Peterson, C.L., and Robert, F. (2015). The histone chaperones FACT and Spt6 restrict H2A.Z from intragenic locations. Mol. Cell *58*, 1113–1123.

Jeronimo, C., Poitras, C., and Robert, F. (2019). Histone recycling by FACT and Spt6 during transcription prevents the scrambling of histone modifications. Cell Rep. *28*, 1206–1218.e8.

Johnson, S.J., Close, D., Robinson, H., Vallet-Gely, I., Dove, S.L., and Hill, C.P. (2008). Crystal structure and RNA binding of the Tex protein from Pseudomonas aeruginosa. J. Mol. Biol. *377*, 1460–1473.

Jürges, C., Dölken, L., and Erhard, F. (2018). Dissecting newly transcribed and old RNA using GRAND-SLAM. Bioinformatics *34*, i218–i226.

Kaplan, C.D., Morris, J.R., Wu, C., and Winston, F. (2000). Spt5 and spt6 are associated with active transcription and have characteristics of general elongation factors in D. melanogaster. Genes Dev. *14*, 2623–2634.

Kaplan, C.D., Laprade, L., and Winston, F. (2003). Transcription elongation factors repress transcription initiation from cryptic sites. Science *301*, 1096–1099.

Kato, H., Okazaki, K., Iida, T., Nakayama, J., Murakami, Y., and Urano, T. (2013). Spt6 prevents transcription-coupled loss of posttranslationally modified histone H3. Sci. Rep. *3*, 2186.

Langmead, B., and Salzberg, S.L. (2012). Fast gapped-read alignment with Bowtie 2. Nat. Methods *9*, 357–359.

Lorch, Y., and Kornberg, R.D. (2015). Chromatin-remodeling and the initiation of transcription. Q. Rev. Biophys. *48*, 465–470.

McCullough, L., Connell, Z., Petersen, C., and Formosa, T. (2015). The abundant histone chaperones Spt6 and FACT collaborate to assemble, inspect, and maintain chromatin structure in Saccharomyces cerevisiae. Genetics *201*, 1031–1045.

Morales, J.C., Richard, P., Patidar, P.L., Motea, E.A., Dang, T.T., Manley, J.L., and Boothman, D.A. (2016). XRN2 links transcription termination to DNA damage and replication stress. PLoS Genet. *12*, e1006107.

Muhar, M., Ebert, A., Neumann, T., Umkehrer, C., Jude, J., Wieshofer, C., Rescheneder, P., Lipp, J.J., Herzog, V.A., Reichholf, B., et al. (2018). SLAM-seq defines direct gene-regulatory functions of the BRD4-MYC axis. Science *360*, 800–805.

Natsume, T., Kiyomitsu, T., Saga, Y., and Kanemaki, M.T. (2016). Rapid protein depletion in human cells by auxin-inducible degron tagging with short homology donors. Cell Rep. *15*, 210–218.

Neri, F., Rapelli, S., Krepelova, A., Incarnato, D., Parlato, C., Basile, G., Maldotti, M., Anselmi, F., and Oliviero, S. (2017). Intragenic DNA methylation prevents spurious transcription initiation. Nature *543*, 72–77.

Ni, Z., Saunders, A., Fuda, N.J., Yao, J., Suarez, J.-R., Webb, W.W., and Lis, J.T. (2008). P-TEFb is critical for the maturation of RNA polymerase II into productive elongation in vivo. Mol. Cell. Biol. *28*, 1161–1170.

Nishimura, K., Fukagawa, T., Takisawa, H., Kakimoto, T., and Kanemaki, M. (2009). An auxin-based degron system for the rapid depletion of proteins in nonplant cells. Nat. Methods *6*, 917–922.

Nojima, T., Gomes, T., Grosso, A.R.F., Kimura, H., Dye, M.J., Dhir, S., Carmo-Fonseca, M., and Proudfoot, N.J. (2015). Mammalian NET-seq reveals genome-wide nascent transcription coupled to RNA processing. Cell *161*, 526–540.

Nojima, T., Tellier, M., Foxwell, J., Ribeiro de Almeida, C., Tan-Wong, S.M., Dhir, S., Dujardin, G., Dhir, A., Murphy, S., and Proudfoot, N.J. (2018). Deregulated expression of mammalian lncRNA through loss of SPT6 induces R-loop formation, replication stress, and cellular senescence. Mol. Cell *72*, 970–984.e7.

O'Leary, N.A., Wright, M.W., Brister, J.R., Ciufo, S., Haddad, D., McVeigh, R., Rajput, B., Robbertse, B., Smith-White, B., Ako-Adjei, D., et al. (2016). Reference sequence (RefSeq) database at NCBI: current status, taxonomic expansion, and functional annotation. Nucleic Acids Res. *44* (D1), D733–D745.

Obara, E.A.A., Aguilar-Morante, D., Rasmussen, R.D., Frias, A., Vitting-Serup, K., Lim, Y.C., Elbæk, K.J., Pedersen, H., Vardouli, L., Jensen, K.E., et al. (2020). SPT6-driven error-free DNA repair safeguards genomic stability of glioblastoma cancer stem-like cells. Nat. Commun. *11*, 4709.

Orlando, D.A., Chen, M.W., Brown, V.E., Solanki, S., Choi, Y.J., Olson, E.R., Fritz, C.C., Bradner, J.E., and Guenther, M.G. (2014). Quantitative ChIP-seq normalization reveals global modulation of the epigenome. Cell Rep. *9*, 1163–1170.

Parua, P.K., Booth, G.T., Sansó, M., Benjamin, B., Tanny, J.C., Lis, J.T., and Fisher, R.P. (2018). A Cdk9-PP1 switch regulates the elongation-termination transition of RNA polymerase II. Nature *558*, 460–464.

Parua, P.K., Kalan, S., Benjamin, B., Sansó, M., and Fisher, R.P. (2020). Distinct Cdk9-phosphatase switches act at the beginning and end of elongation by RNA polymerase II. Nat. Commun. *11*, 4338.

Pathak, R., Singh, P., Ananthakrishnan, S., Adamczyk, S., Schimmel, O., and Govind, C.K. (2018). Acetylation-dependent recruitment of the FACT complex and its role in regulating Pol II occupancy genome-wide in *Saccharomyces cerevisiae*. Genetics *209*, 743–756.

Perales, R., Erickson, B., Zhang, L., Kim, H., Valiquett, E., and Bentley, D. (2013). Gene promoters dictate histone occupancy within genes. EMBO J. *32*, 2645–2656.

Quinlan, A.R. (2014). BEDTools: the Swiss-Army tool for genome feature analysis. Curr. Protoc. Bioinform. *47*, 11.12.11–11.12.34.

Ramírez, F., Ryan, D.P., Grüning, B., Bhardwaj, V., Kilpert, F., Richter, A.S., Heyne, S., Dündar, F., and Manke, T. (2016). deepTools2: a next generation web server for deep-sequencing data analysis. Nucleic Acids Res. *44* (W1), W160–W165.

Ran, F.A., Hsu, P.D., Wright, J., Agarwala, V., Scott, D.A., and Zhang, F. (2013). Genome engineering using the CRISPR-Cas9 system. Nat. Protoc. *8*, 2281–2308.

Rappsilber, J., Ishihama, Y., and Mann, M. (2003). Stop and go extraction tips for matrix-assisted laser desorption/ionization, nanoelectrospray, and LC/MS sample pretreatment in proteomics. Anal. Chem. *75*, 663–670.

Rutkowski, A.J., Erhard, F., L'Hernault, A., Bonfert, T., Schilhabel, M., Crump, C., Rosenstiel, P., Efstathiou, S., Zimmer, R., Friedel, C.C., and Dölken, L. (2015). Widespread disruption of host transcription termination in HSV-1 infection. Nat. Commun. *6*, 7126.

Schlackow, M., Nojima, T., Gomes, T., Dhir, A., Carmo-Fonseca, M., and Proudfoot, N.J. (2017). Distinctive patterns of transcription and RNA processing for human lincRNAs. Mol. Cell *65*, 25–38.

Schneider, C.A., Rasband, W.S., and Eliceiri, K.W. (2012). NIH Image to ImageJ: 25 years of image analysis. Nat. Methods *9*, 671–675.

Sdano, M.A., Fulcher, J.M., Palani, S., Chandrasekharan, M.B., Parnell, T.J., Whitby, F.G., Formosa, T., and Hill, C.P. (2017). A novel SH2 recognition mechanism recruits Spt6 to the doubly phosphorylated RNA polymerase II linker at sites of transcription. eLife *6*, e28723.

Shen, L., Shao, N., Liu, X., and Nestler, E. (2014). ngs.plot: Quick mining and visualization of next-generation sequencing data by integrating genomic databases. BMC Genomics *15*, 284.

Skourti-Stathaki, K., and Proudfoot, N.J. (2014). A double-edged sword: R loops as threats to genome integrity and powerful regulators of gene expression. Genes Dev. *28*, 1384–1396.

Teves, S.S., Weber, C.M., and Henikoff, S. (2014). Transcribing through the nucleosome. Trends Biochem. Sci. *39*, 577–586.

Uwimana, N., Collin, P., Jeronimo, C., Haibe-Kains, B., and Robert, F. (2017). Bidirectional terminators in Saccharomyces cerevisiae prevent cryptic transcription from invading neighboring genes. Nucleic Acids Res. *45*, 6417–6426.

Venkatesh, S., and Workman, J.L. (2015). Histone exchange, chromatin structure and the regulation of transcription. Nat. Rev. Mol. Cell Biol. *16*, 178–189.

Viktorovskaya, O., Chuang, J., Jain, D., Reim, N.I., López-Rivera, F., Murawska, M., Spatt, D., Churchman, L.S., Park, P.J., and Winston, F. (2021). Essential histone chaperones collaborate to regulate transcription and chromatin integrity. Genes Dev. *35*, 698–712.

Vilborg, A., Passarelli, M.C., Yario, T.A., Tycowski, K.T., and Steitz, J.A. (2015). Widespread inducible transcription downstream of human genes. Mol. Cell *59*, 449–461.

Vos, S.M., Farnung, L., Boehning, M., Wigge, C., Linden, A., Urlaub, H., and Cramer, P. (2018). Structure of activated transcription complex Pol II-DSIF-PAF-SPT6. Nature *560*, 607–612.

Walz, S., Lorenzin, F., Morton, J., Wiese, K.E., von Eyss, B., Herold, S., Rycak, L., Dumay-Odelot, H., Karim, S., Bartkuhn, M., et al. (2014). Activation and repression by oncogenic MYC shape tumour-specific gene expression profiles. Nature *511*, 483–487.

West, S., Gromak, N., and Proudfoot, N.J. (2004). Human 5′–> 3′ exonuclease Xrn2 promotes transcription termination at co-transcriptional cleavage sites. Nature *432*, 522–525.

Winston, F., Chaleff, D.T., Valent, B., and Fink, G.R. (1984). Mutations affecting Ty-mediated expression of the HIS4 gene of Saccharomyces cerevisiae. Genetics *107*, 179–197.

Wissink, E.M., Vihervaara, A., Tippens, N.D., and Lis, J.T. (2019). Nascent RNA analyses: tracking transcription and its regulation. Nat. Rev. Genet. *20*, 705–723.

Yoh, S.M., Cho, H., Pickle, L., Evans, R.M., and Jones, K.A. (2007). The Spt6 SH2 domain binds Ser2-P RNAPII to direct Iws1-dependent mRNA splicing and export. Genes Dev. *21*, 160–174.

Youdell, M.L., Kizer, K.O., Kisseleva-Romanova, E., Fuchs, S.M., Duro, E., Strahl, B.D., and Mellor, J. (2008). Roles for Ctk1 and Spt6 in regulating the different methylation states of histone H3 lysine 36. Mol. Cell. Biol. *28*, 4915–4926.

Zhang, Y., Liu, T., Meyer, C.A., Eeckhoute, J., Johnson, D.S., Bernstein, B.E., Nusbaum, C., Myers, R.M., Brown, M., Li, W., and Liu, X.S. (2008). Model-based analysis of ChIP-Seq (MACS). Genome Biol. *9*, R137.

Zhang, Y., Xue, W., Li, X., Zhang, J., Chen, S., Zhang, J.L., Yang, L., and Chen, L.L. (2016). The biogenesis of nascent circular RNAs. Cell Rep. *15*, 611–624.

STAR★METHODS

KEY RESOURCES TABLE

REAGENT or RESOURCE	SOURCE	IDENTIFIER
Antibodies		
Rabbit polyclonal anti-SPT6	Novus Biologicals	Cat# NB100-2582; RRID: AB_609125; Lot: A1
Mouse monoclonal anti-vinculin (clone*hv)	Sigma-Aldrich	Cat# V9131; RRID: AB_477629; Lot: 034M4809V
Rabbit monoclonal anti-V5-Tag (D3H8Q)	Cell Signaling Technology	Cat# 13202S; RRID: AB_2687461; Lot: 6
Rabbit monoclonal anti-CDK2 (78B2)	Cell Signaling Technology	Cat# 2546S; RRID: AB_2276129; Lot: 6
Mouse monoclonal anti-Pol II (A-10)	Santa Cruz Biotechnology	Cat# sc-17798; RRID: AB_677355; Lot: L0418
Rabbit polyclonal anti-RNA polymerase II CTD (phospho S2) antibody	Abcam	Cat# ab5095; RRID: AB_304749; Lot: GR3231908-7
Rabbit monoclonal anti-SUPT5H [EPR5145(2)]	Abcam	Cat# ab126592; RRID: AB_11128976; Lot: GR79342-8
Rabbit polyclonal anti-RTF1 antibody	Bethyl Laboratories	Cat# A300-179A; RRID: AB_2185963; Lot: 1
Rabbit monoclonal anti-SPT16 (D7I2K) antibody	Cell Signaling Technology	Cat# 12191S; RRID: AB_2732025; Lot: 1
Mouse polyclonal anti-CTR9 antibody	Bethyl Laboratories	Cat# A301-395A; RRID: AB_960973; Lot: 4
Mouse monoclonal anti-SSRP1 (clone 10D1)	BioLegend	Cat# 609702; RRID: AB_315731; Lot: B201389
Rabbit polyclonal anti - α-tubulin antibody (E-19)	Santa Cruz Biotechnology	Cat# sc-12462-R; RRID: AB_2241125; Lot: D2506
Rabbit polyclonal Anti-TFIIF antibody	Abcam	Cat# ab28179; RRID: AB_2114552; Lot: GR3200189-8
Mouse monoclona Anti-TATA binding protein TBP antibody [1TBP18]	Abcam	Cat# ab818; RRID: AB_306337; Lot: GR3261953-6
Rabbit polyclonal anti-HA-probe (Y-11)	Santa Cruz Biotechnology	Cat# sc-805X; RRID: AB_631618; Lot: H1215
Rabbit polyclonal anti-CSTF64 antibody	Bethyl Laboratories	Cat# A301-092A; RRID: AB_873014; Lot: 2
Rabbit polyclonal gamma H2A.X (phospho S139) - used for ChIP	Abcam	Cat# ab2893; RRID: AB_303388; Lot: GR3270858-1
Rabbit Phospho-Histone H2A.X (Ser139) Antibody	Cell Signaling Technology	Cat# 2577L; RRID: AB_2118010; Lot: 12
Rabbit Anti-H2A.X Polyclonal Antibody, Unconjugated	Abcam	Cat# ab11175; RRID: AB_297814; Lot: GR269626-10
Rabbit polyclonal anti-Histone H3	Abcam	Cat# ab1791; RRID: AB_302613; Lot: GR3297885-1
Mouse RPA 32 kDa subunit (MA34) antibody	Santa Cruz Biotechnology	Cat# sc-53496; RRID: AB_670974; Lot: H2906
Rabbit Phospho RPA32 (S33) Antibody	Bethyl Laboratories	Cat# A300-246A; RRID: AB_2180847; Lot: 8
Rabbit polyclonal Anti-RNA pol II CTD phospho Thr4	Active Motif	Cat# 61308; RRID: AB_2793588; Lot: 13912001
Mouse recombinant AbFlex® RNA Pol II CTD phospho Tyr1 antibody	Active Motif	Cat# 91220; RRID: AB_2793809; Lot: 00418001
Rabbit monoclonal Anti-GAPDH Antibody	Cell Signaling Technology	Cat# 2118; RRID: AB_561053; Lot: 10
Mouse monoclonal Pol II (8WG16) antibody	Santa Cruz Biotechnology	Cat# sc-56767; RRID: AB_785522; Lot: A0821
Mouse monoclonal XRN2 (H-3) antibody	Santa Cruz Biotechnology	Cat# sc-365258; RRID: AB_10846079; Lot: L0211

(*Continued on next page*)

CellPress
OPEN ACCESS

Continued

REAGENT or RESOURCE	SOURCE	IDENTIFIER
Rabbit polyclonal Anti-PCF11 Antibody	Bethyl Laboratories	Cat# A303-706A; RRID: AB_11204946; Lot: 706A-1
Mouse monoclonal Anti-Histone H2A.X, phospho (Ser139) antibody, Unconjugated, (jbw301)	Millipore	Cat# 05-636; RRID: AB_309864; Lot: 3524749
Mouse monoclonal Pol II (CTD4H8) antibody	Santa Cruz Biotechnology	Cat# sc-47701; RRID: AB_677353; Lot: #C1014
Mouse Anti-DNA-RNA Hybrid [S9.6] Antibody	Kerafast	Cat# ENH001; RRID: AB_2687463; Lot: 071718_4
ECL-Anti-rabbit IgG Horseradish Peroxidase	GE Healthcare	Cat# NA934V; RRID: AB_772206; Lot: 1079-0198
ECL-Anti-mouse IgG Horseradish Peroxidase	GE Healthcare	Cat# NA931V; RRID: AB_772210; Lot: 1708-9105
FITC anti-BrdU, Mouse IgG1, kappa (clone 3D4)	BioLegend	Cat# 364104; RRID: AB_2564481; Lot: B264755
Bacterial and virus strains		
pRRL-hygro (empty vector)	Eilers Lab	N/A
pRRL-hygro-TIR1	This paper	N/A
pRRL-puro (empty vector)	Walz et al., 2014	N/A
pRRL-puro-RPB3-HA	Baluapuri et al., 2019	N/A
Chemicals, peptides, and recombinant proteins		
DMEM, high glucose, pyruvate	Thermo Fisher Scientific	Cat#41966052
Fetal Bovine Serum Advanced	Capricorn Scientific GmbH	Cat#FBS-11A
Penicillin-Streptomycin	Sigma-Aldrich	Cat#P4333
Hygromycin	InvivoGen	Cat#ant-hg-5
Blasticidin	InvivoGen	Cat#ant-bl
Puromycin	InvivoGen	Cat#ant-pr-1
Auxin (Indole-3-acetic acid sodium salt)	Sigma-Aldrich	Cat#i5148-2G
SpeI-HF	New England BioLabs	Cat#R3133L
MluI-HF	New England BioLabs	Cat#R3198L
AgeI-HF	New England BioLabs	Cat#R3552L
BbsI-HF	New England BioLabs	Cat#R3539L
EcoRI-HF	New England BioLabs	Cat#R3101L
BamHI-HF	New England BioLabs	Cat#R3136L
Polybrene	Sigma-Aldrich	Cat#H9268
Lipofectamine® RNAiMAX Transfection Reagent	Thermo Fisher Scientific	Cat#13778-150
Protease Inhibitor Cocktail	Sigma-Aldrich	Cat#P8340
Phosphatase Inhibitor Cocktail 2	Sigma-Aldrich	Cat#P5726
Phosphatase Inhibitor Cocktail 3	Sigma-Aldrich	Cat#P0044
Immobilon-FL, PVDF Membran	Merck Millipore	Cat#IPFL00010
Benzonase nuclease purity > 99% 25U/µl	Merck Millipore	Cat#70664-3
4-thiouridine (4sU)	Sigma-Aldrich	Cat#T4509
GlycoBlue Coprecipitant (15 mg/mL)	Thermo Fisher Scientific	Cat#AM9516
DRB (5,6-Dichlorobenzimidazole 1-β-D-ribofuranoside)	Sigma-Aldrich	Cat#D1916
Dynabead Protein A for Immunoprecipitation	Thermo Fisher Scientific	Cat#10002D
Dynabead Protein G for Immunoprecipitation	Thermo Fisher Scientific	Cat#10004D

(*Continued on next page*)

Continued

REAGENT or RESOURCE	SOURCE	IDENTIFIER
Dynabeads® MyOne Streptavidin T1	Thermo Fisher Scientific	Cat#65601
Pierce Anti-HA Magnetic Beads	Thermo Fisher Scientific	Cat#88836
NuPAGE LDS Sample Buffer (4X)	Thermo Fisher Scientific	Cat#NP0007
Proteinase K	Carl Roth	Cat#7528.2
M-MLV Reverse Transcriptase	Promega	Cat#M1701
ERCC RNA Spike-In Mix	Thermo Fisher Scientific	Cat#4456740
Pierce Iodoacetamide No-Weigh Format	Thermo Fisher Scientific	Cat#A39271
Thermo Scientific Pierce DTT (Dithiothreitol), No-Weigh Format	Thermo Fisher Scientific	Cat#A39255
Hoechst 33342 Ready Flow Reagent	Thermo Fisher Scientific	Cat#R37165
Alexa Fluor 488 Phalloidin	Thermo Fisher Scientific	Cat#A12379
Fluoromount Aqueous Mounting Medium	Sigma-Aldrich	Cat#F4680-25ML
Critical commercial assays		
Phusion High-Fidelity DNA Polymerase (2 U/µL)	Thermo Fisher Scientific	Cat#F530L
CloneJET PCR Cloning Kit	Thermo Fisher Scientific	Cat#K1231
ON-TARGETplus Non-targeting Pool	Horizon Discovery	Cat#D-001810-10-50
ON-TARGETplus Human SPT6 SMARTpool	Horizon Discovery	Cat#L-010540-00-0020
Immobilon Western Chemiluminescent HRP Substrate	Merck Millipore	Cat#WBKLS0500
Quant-iT PicoGreen dsDNA assay	Thermo Fisher Scientific	Cat#P7589
Quant-iT RiboGreen RNA Assay Kit	Thermo Fisher Scientific	Cat#R11490
PowerUP SYBR Green Master Mix	Thermo Fisher Scientific	Cat#A25778
NEBNext Ultra II DNA Library Prep Kit for Illumina	New England BioLabs	Cat#E7645S
NEBNext Ultra II Directional RNA Library Prep with Beads	New England BioLabs	Cat#E7765L
NEBNext rRNA Depletion Kit (Human/ Mouse/Rat)	New England BioLabs	Cat#E6310
RNeasy MinElute Cleanup Kit	QIAGEN	Cat#74204
miRNeasy MiniKit	QIAGEN	Cat# 217004
QuantSeq 3′ mRNA-Seq Library Prep Kit (FWD) for Illumina	Lexogen	Cat#015.24
UMI Second Strand Synthesis Module for QuantSeq FWD (Illumina, Read 1)	Lexogen	Cat#081.96
PCR Add-on Kit for Illumina	Lexogen	Cat#020.96
NextSeq 500/550 High Output Kit v2 (75 cycles)	Illumina	Cat#FC-404-2005
NGS Fragment High Sensitivity Analysis Kit, 1-6,000 bp	Agilent Technologies	Cat #DNF-474-0500
Duolink® *In Situ* Detection Reagents Red	Sigma-Aldrich	Cat#DUO92008
Duolink® *In Situ* PLA® Probe Anti-Mouse MINUS	Sigma-Aldrich	Cat#DUO92004
Duolink® *In Situ* PLA® Probe Anti-Rabbit PLUS	Sigma-Aldrich	Cat#DUO92002
Deposited data		
RNAPII interactome data	This paper	PRIDE data: PXD025243
Raw and analyzed data	This paper	GEO: GSE162264
Unprocessed image files	This paper	Mendeley data: https://doi.org/10.17632/rxcmx677cx.1

(*Continued on next page*)

CellPress
OPEN ACCESS

Continued

REAGENT or RESOURCE	SOURCE	IDENTIFIER
Experimental models: Cell lines		
NIH 3T3	ATCC	CVCL_0594
U2OS	ATCC	N/A
HEK293TN	ATCC	CRT-11268
U2OS$^{SPT6-CAID}$	This paper	N/A
Oligonucleotides		
All oligonucleotides used in this study are listed in Table S4	This paper	N/A
Recombinant DNA		
pJET-CAID-Blast-entry-vector	This paper	N/A
pJET-SPT6_HDR	This paper	N/A
pSpCas9(BB)-2A-GFP (PX458)	Zhang Lab	Addgene #48138
PX458_SPT6_sgR1	This paper	N/A
PX458_SPT6_sgR2	This paper	N/A
pBABE TIR1-9myc	Cleveland Lab	Addgene #64945
psPAX2	Trono Lab	Addgene #12260
pMD2.G	Trono Lab	Addgene #12259
Software and algorithms		
ImageJ v1.51	Schneider et al., 2012	RRID:SCR_003070; https://imagej.net/software/imagej
Image Studio Lite v5.2.5	LI-COR Biosciences – GmbH	RRID:SCR_013715; https://www.licor.com/bio/image-studio-lite/
Bowtie2 v2.2.7	Langmead and Salzberg, 2012	RRID:SCR_005476; http://bowtie-bio.sourceforge.net/index.shtml
Bowtie2 v2.3.4.1	Langmead and Salzberg, 2012	RRID:SCR_005476; http://bowtie-bio.sourceforge.net/index.shtml
SAMtools v1.3.1	N/A	RRID:SCR_002105; http://samtools.sourceforge.net
SAMtools v1.7	N/A	RRID:SCR_002105; http://samtools.sourceforge.net
Bedtools v2.26.0	Quinlan, 2014	RRID:SCR_006646; https://github.com/arq5x/bedtools2/releases
Integrated Genome Browser v9.1.4	Freese et al., 2016	RRID:SCR_011792; https://bioviz.org/index.html
BD FACSDIVA Software v6.1.2	BD Biosciences	RRID:SCR_001456; https://www.bdbiosciences.com/en-eu/instruments/research-instruments/research-software/flow-cytometry-acquisition/facsdiva-software
FlowJo v8.8.6	BD Biosciences	RRID:SCR_008520; https://www.flowjo.com/
StepOne software v2.3	Thermo Fisher Scientific	RRID:SCR_014281; https://www.thermofisher.com/us/en/home/technical-resources/software-downloads/StepOne-and-StepOnePlus-Real-Time-PCR-System.html
Deeptools v3.3.0	Ramírez et al., 2016	RRID:SCR_016366; https://deeptools.readthedocs.io/en/develop/index.html
ngsplot v2.41.3	Shen et al., 2014	RRID:SCR_011795; https://github.com/shenlab-sinai/ngsplot/
R version 3.6.1	NA	RRID:SCR_001905; https://www.r-project.org/

(*Continued on next page*)

Continued		
REAGENT or RESOURCE	SOURCE	IDENTIFIER
MACS2	Zhang et al., 2008	RRID:SCR_013291; https://github.com/macs3-project/MACS/wiki
MEME Suite	Bailey et al., 2009	RRID:SCR_001783; https://meme-suite.org/index.html
STAR v2.5.3a	Dobin et al., 2013	RRID:SCR_015899; https://github.com/alexdobin/STAR/releases
GRAND-SLAM v2.0.5g	Jürges et al., 2018	https://github.com/erhard-lab/gedi/wiki/GRAND-SLAM
GraphPad Prism v9.1.0	N/A	RRID:SCR_002798; https://www.graphpad.com/scientific-software/prism/
SonoLab Software	N/A	RRID:SCR_016302; https://www.covaris.com/products-services/instruments/sonolab-software
MaxQuant v1.6.2.2 performed with Andromeda	Cox and Mann, 2008	RRID:SCR_014485; https://www.maxquant.org/
Code from this paper: Zenodo depository	This paper	https://doi.org/10.5281/zenodo.4275956

RESOURCE AVAILABILITY

Lead contact

Further information and requests for resources and reagents should be directed to and will be fulfilled by the Lead Contact, Elmar Wolf (elmar.wolf@biozentrum.uni-wuerzburg.de).

Materials availability

Plasmids and cell lines generated in this study are available on request from the Lead Contact.

Data and code availability

Primary sequencing data and bedGraphs are deposited at the GEO depository: GSE162264. Modeling results are accessible at https://erhard-lab.de/web-platforms. Code is available at Zenodo depository: https://doi.org/10.5281/zenodo.4275956. Unprocessed image files are available at Mendeley Data: https://doi.org/10.17632/rxcmx677cx.1. Quantitative MS proteomics data have been deposited at the ProteomeXchange Consortium via the PRIDE partner repository: PXD025243.

EXPERIMENTAL MODEL AND SUBJECT DETAILS

Cell culture and auxin treatment

Human U2OS (female), human HEK293 (female) and murine NIH 3T3 (male) cells were cultured in DMEM (Thermo Fisher Scientific) supplemented with 10% FBS (Capricorn Scientific), 100 U/ml penicillin and 100 μg/ml streptomycin (Sigma-Aldrich), at 37°C, 5% CO_2. PCR screening for mycoplasma contamination was done routinely; the cells always tested negative. For auxin-induced degradation, an aqueous stock solution of 500 mM auxin (indole-3-acetic acid sodium salt, Sigma-Aldrich) was prepared. U2OS cells were incubated with or without 500 μM auxin in culture medium for different times.

Endogenous knock-in of AID tag and cell line transfection

To obtain stable cell lines expressing endogenous SPT6 with an AID tag at the C-terminal, U2OS cells were grown in 6-well dishes and transfected with pJET-SPT6_HDR and PX458_SPT6_sgR1 or PX458_SPT6_sgR2 plasmids using polyethylenimine (PEI). After 48 h, cells were selected by adding 7.5 μg/ml blasticidin (InvivoGen). After 6 days, cells were split into 15 cm dishes and cultured in blasticidin-containing medium. After 12 days, colonies were picked and transferred to 24-well plates. Individual clones were evaluated using genomic PCR (see below). For TIR1 expression, plasmid pRRL-hygro-TIR1 and the lentiviral packaging plasmids psPAX2 (Addgene #12260) and pMD2.G (Addgene #12259) were transfected into HEK293 cells using PEI. The virus-containing medium was filtered (0.45 μm) and used to infect $U2OS^{WT}$ and $U2OS^{SPT6\text{-}AID}$ cells. Cells were selected with 150 μg/ml hygromycin (InvivoGen) starting 48 h after infection. For RNA interference, an siRNA pool against SPT6 and a negative control (Horizon Discovery) were used for transfection using RNAiMAX transfection reagent (Thermo Fisher Scientific). Cells were harvested for protein (for immunoblotting) or RNA (for 4sU-seq) 48 h after transfection. For HA-RPB3 expression, pRRL-puro-RPB3-HA was transduced and selection was done using 2 μg/mL puromycin (InvivoGen).

METHOD DETAILS

General cloning and plasmid constructs

The AID sequence was designed as published earlier (Muhar et al., 2018). For homozygous tagging of SPT6, pJET-CAID-Blast-entry-vector was constructed using the AID sequence. Following the strategy for knock-in Natsume et al. (2016), to obtain the homology-directed repair (HDR) template, homology arms (HA) were amplified by PCR (sequences of oligonucleotides are listed in Table S4) using U2OS genomic DNA as template (5′HA, 400 bp; 3′HA, 800 bp). PCR fragments were digested with AgeI/EcoRI (5′HA) or BamHI/SpeI (3′HA) and cloned into the entry vector to obtain pJET-SPT6_HDR (5′HA-AID-V5-P2A-Blast-TGA-3′HA). To construct CRISPR/Cas9 vectors, two sgRNA were cloned into PX458 (Addgene #48138) as described (Ran et al., 2013) to obtain PX458_SPT6_sgR1 and PX458_SPT6_sgR2. 9x-myc-tagged TIR1 was PCR amplified using the template vector pBABE TIR1-9myc (Addgene #64945) and inserted into pRRL-hygro using AgeI/MluI digestion to obtain pRRL-hygro-TIR1.

Genomic PCR

To genotype clones, genomic DNA was isolated. Briefly, cells were lysed in lysis buffer (10 mM Tris HCl, pH 8.0, 100 mM NaCl, 10 mM EDTA, 0.5% (w/v) SDS, 0.08 mg/ml proteinase K (Carl Roth)) at 37°C for 2 h. Then, saturated saline solution was added and samples were incubated on ice, followed by centrifugation (5,000 g, 10 min, 4°C). The supernatant was collected and precipitated with 0.75 volume of isopropanol. After centrifugation (17,000 g, 10 min, 4°C), the pellet was washed with 70% ethanol and dissolved in water to a final DNA concentration of 100 ng/μl. Genomic PCR was carried out using primers and Phusion Polymerase (Thermo Fisher Scientific) with the following protocol for 25 cycles: 98°C / 10 s → 63°C / 10 s → 72°C / 90 s.

Immunoblotting

Cells were lysed in RIPA lysis buffer (50 mM HEPES pH 7.9, 140 mM NaCl, 1 mM EDTA, 1% Triton X-100, 0.1% SDS, 0.1% sodium deoxycholate) with phosphatase and protease inhibitor cocktails (Sigma-Aldrich) at 4°C head-over-tail for 20 min. After centrifugation, the supernatant was collected. Protein was quantified using the BCA assay, and equal amounts of protein were separated using Bis-Tris-PAGE. The separated proteins were transferred to PVDF membranes (Merck Millipore) and incubated with 5% (w/v) non-fat dry milk in TBS-T (20 mM Tris-HCl, pH 7.5, 150 mM NaCl, 0.1% (v/v) Tween-20) for 1 h at room temperature for blocking. The membranes were washed and incubated with primary antibodies overnight at 4°C. To visualize the bands, horseradish peroxidase (HRP)-labeled secondary antibodies were used and detected using chemiluminescent HRP substrate (Merck Millipore) in LAS4000 Mini (Fuji). The signal was quantified using Image Studio Lite (LI-COR Biosciences, v5.2.5).

Chromatin IP with reference exogenous genome spike-in and deep sequencing (ChIP-Rx)

Chromatin preparation and immunoprecipitation: For each immunoprecipitation condition, 50 million cells were crosslinked with formaldehyde (final concentration, 1%) for 5 min at room temperature, as described (Walz et al., 2014). Fixation was stopped by adding glycine (final concentration, 125 mM) to the medium and incubating at room temperature for 5 min. Cells were washed twice with ice-cold PBS and harvested in PBS freshly supplemented with protease and phosphatase inhibitors (Sigma-Aldrich). The buffers used in all further steps were freshly supplemented with protease and phosphatase inhibitors.

For the spike-in of exogenous DNA, murine NIH 3T3 cells were added in a 1:10 cell ratio to the samples. Then, samples were lysed in lysis buffer I (5 mM PIPES pH 8.0, 85 mM KCl, 0.5% NP-40) at 4°C for 20 min. Nuclei were collected by centrifugation (1,500 rpm for 20 min at 4°C) and the pellets were dissolved in lysis buffer II (10 mM Tris pH 7.5, 150 mM NaCl, 1 mM EDTA, 1% NP-40, 1% sodium deoxycholate, 0.1% SDS). To fragment the crosslinked chromatin, samples were sonicated using a Covaris Focused Ultrasonicator M220 for 50 min/ml lysate. A fragment size distribution of 150-300 bp was verified by agarose gel electrophoresis. Sheared chromatin was centrifuged (20 min at 14,000 rpm at 4°C) and the supernatant was taken as the input for immunoprecipitation.

For immunoprecipitation, 100 μL Dynabeads Protein A and Protein G (Thermo Fisher Scientific) were pre-incubated, overnight with rotation, with 5 g/l BSA and 15 μg of an antibody against SPT6 (Novus Biologicals, #NB100-2582), pS2-RNAPII (Abcam, #ab5095), total RNAPII (Santa Cruz Biotechnology A-10, #sc-17798), TBP (Abcam, #ab818), CSTF2 (Bethyl Laboratories, #A301-092A), gamma H2AX (Abcam, #ab2893), or histone-H3 (Abcam, #ab1791). After washing the antibody-coupled beads thrice with 5 g/l BSA, sheared chromatin was added and incubated for 6 h at 4°C with rotation. Then, the beads were washed thrice with washing buffer I (20 mM Tris pH 8.1, 150 mM NaCl, 2 mM EDTA, 1% Triton X-100, 0.1% SDS), washing buffer II (20 mM Tris pH 8.1, 500 mM NaCl, 2 mM EDTA, 1% Triton X-100, 0.1% SDS), washing buffer III (10 mM Tris pH 8.1, 250 mM LiCl, 1 mM EDTA, 1% NP-40, 1% sodium deoxycholate; including a 5 min incubation step with rotation), and TE buffer (Thermo Fisher Scientific). Chromatin–protein complexes were eluted twice from the beads by incubating with 150 μL freshly prepared elution buffer (100 mM $NaHCO_3$, 1% SDS) for 15 min at room temperature, with rotation. De-crosslinking of the eluted and input samples was done overnight, followed by protein digestion with proteinase K (Carl Roth) and RNA digestion with RNase A. The DNA was purified by phenol-chloroform extraction and precipitated with ethanol. The resulting ChIP DNA pellets were dissolved in water.

ChIP qPCR: To assess the efficiency of immunoprecipitation, ChIP DNA pellets were analyzed by qPCR on a StepOnePlus Real-Time PCR System (Thermo Fisher Scientific) using the SYBR Green Master Mix (Thermo Fisher Scientific). Equal amounts of ChIP

DNA and SYBR Green Master Mix were added along with 0.5 μM primers. qPCR assays were done in technical triplicates. For observing pS2-RNAPII occupancy downstream of PAS, all values were normalized to the values corresponding to primers located upstream of PAS.

Library preparation and sequencing: For ChIP-Rx sequencing, qPCR-verified ChIP DNA was quantified using Quant-iT PicoGreen dsDNA assay (Thermo Fisher Scientific). Library preparation was done using the NEBNext Ultra II DNA Library Prep Kit for Illumina. The libraries were amplified using 12-17 PCR cycles depending on the input. The concentration and size distribution of the library were evaluated on the Fragment Analyzer (Agilent Technologies) using the NGS Fragment High Sensitivity Analysis Kit (1-6,000 bp; Agilent Technologies). The libraries were sequenced on a NextSeq500 Illumina platform for 75 cycles. Base calling was performed using Illumina's BaseSpace platform.

ChIP-Rx BAM file conversion and visualization: From the obtained FASTQ files, first the sequencing quality was checked using FastQC script. To map human reads, Bowtie2 v2.2.7 (Langmead and Salzberg, 2012) (-N 1) was used with hg19 as reference genome. For mouse reads, mm10 was used as the reference genome. Mouse reads were used for spike normalization based on a scaling factor calculated for each ChIP-Rx dataset as described (Orlando et al., 2014). The BAM files obtained after spike normalization were sorted according to the chromosome using SAMtools v1.3.1 and converted to bedGraphs using Bedtools v2.26.0 (Quinlan, 2014). bedGraphs for input were prepared by using BAM files obtained by combining input from this experiment and published dataset (Baluapuri et al., 2019). To visualize the bedGraphs, Integrated Genome Browser v9.1.4 was used (Freese et al., 2016).

Quantitative PCR

Total RNA was extracted with the miRNeasy kit (QIAGEN) with on-column DNase digestion. Total RNA was converted to cDNA using random primers and M-MLV reverse transcriptase (Promega). Equal amounts of cDNA and SYBR Green Master Mix (Thermo Fisher Scientific) were added along with 0.5 μM intronic primers and analyzed by qPCR on a StepOnePlus Real-Time PCR System (Thermo Fisher Scientific). Assays were done in technical triplicates. To observe processivity defects, values were normalized to primers located proximal to TSS. To observe readthrough defects, values were normalized to primers upstream of PAS.

4sU sequencing

Cells were seeded, and after 18 h treated with auxin or siRNA as described above, and harvested. RNA was labeled with 2 mM 4-thiouridine (4sU) (Sigma-Aldrich) in the last 15 min before harvest. Cells were lysed with QIAzol reagent (QIAGEN). Cell lysates were spiked with of 4sU-treated mouse T-cell lysates. Total RNA was extracted with the miRNeasy kit (QIAGEN) with on-column DNase digestion. RNA was quantified on a Nanodrop spectrophotometer.

RNA (50-70 μg) was biotinylated on thiouridine residues using 0.2 mg/ml EZ-Link Biotin-HPDP (Thermo Fisher Scientific) dissolved in dimethylformamide and biotin-labeling buffer (10 mM Tris pH 7.4, 1 mM EDTA) by incubating for 2 h at 25°C with rotation. RNA was purified using chloroform-isoamyl alcohol (24:1) extraction in MaXtract High Density tubes (QIAGEN). The aqueous phase was collected and diluted with 1:10 volume of 5 M NaCl, 1:1 volume of isopropanol and 1 μL GlycoBlue coprecipitant (Thermo Fisher Scientific). Samples were incubated for 5 min and centrifuged for 20 min (20,000 g at 4°C). The RNA pellets were washed twice with 75% ethanol and allowed to dry to remove residual ethanol. Pellets were dissolved in 100 μL RNase-free water.

To isolate biotinylated RNA, Dynabeads MyOne Streptavidin T1 beads (Thermo Fisher Scientific) were washed as per the vendor's instructions and resuspended in an equal volume of wash buffer (2 M NaCl with 10 mM Tris pH 7.5, 1 mM EDTA, 0.1% Tween 20); 100 μL was added per sample. After incubation at 25°C for 15 min with rotation and magnetic separation, the beads were washed multiple times. 4sU-RNA was eluted with 100 μL of freshly prepared 100 mM DTT (Thermo Fisher Scientific) in nuclease-free water, and purified using RNeasy MinElute Cleanup kit (QIAGEN). 4sU-RNA was quantified with the Quant-iT RiboGreen Assay (Thermo Fisher Scientific).

4sU-RNA was used for library preparation with NEBNext rRNA Depletion Kit (Human/Mouse/Rat) and NEBNext Ultra II Directional RNA Library Prep kit (both from New England Biolabs). The libraries were amplified with 10-14 PCR cycles depending on the input RNA. The concentration and size distribution of the libraries were determined on a Fragment Analyzer using the NGS Fragment High Sensitivity Analysis Kit (1-6,000 bp; Agilent Technologies). The libraries were sequenced on the NextSeq500 Illumina platform for 75 cycles. Base calling was performed using Illumina's BaseSpace platform.

Sequencing quality of FASTQ files was checked using FastQC. Reads were mapped to the human reference genome (hg19) with Bowtie2 v2.3.4.1 (Langmead and Salzberg, 2012). Reads mapping to rRNA gene clusters, exons and regions in the ENCODE Blacklist (Amemiya et al., 2019) were removed from BAM files using Bedtools v2.26.0 (Quinlan, 2014). The BAM files were normalized to read counts and sorted according to the chromosome using SAMtools v1.7. To visualize read alignments on the Integrated Genome Browser v9.1.4, stranded BAM files were generated using SAMtools v1.7 and converted to bedGraphs using Bedtools v2.26.0. For LFC regression analysis, FASTQ files were mapped against a combined index of the human (hg19, Ensembl 86) and mouse (mm10, Ensembl 90) genomes using STAR (2.5.3a) with default parameters (Dobin et al., 2013). Size factors for the normalization were computed by dividing the total number of mouse mapped reads by their median across all samples. Mouse reads and all reads overlapping an exon of the TUs were then discarded.

DRB-4sU sequencing

Cells were treated with auxin for 6 h. Then, 100 μM of 5,6-dichloro-1-beta-D-ribofuranosylbenzimidazole (DRB) (Sigma-Aldrich) was added to reversibly block transcription. After 2.5 h, cells were washed with PBS, and RNA was labeled by adding culture medium containing 2 mM 4sU, with or without auxin, for different times. Cells were harvested directly on the plate with QIAzol reagent (-QIAGEN). 4sU-RNA pulldown was done as described for 4sU-sequencing. The final libraries were sequenced on a NextSeq500 Illumina platform for 75 cycles. Base calling was performed using Illumina's BaseSpace platform.

To assess the efficiency of DRB treatment and release, total RNA was converted to cDNA using random primers and M-MLV reverse transcriptase (Promega). Equal amounts of cDNA and SYBR Green Master Mix (Thermo Fisher Scientific) were added along with 0.5 μM intronic primers at *OPA1* gene and analyzed by qPCR on a StepOnePlus Real-Time PCR System (Thermo Fisher Scientific). Assays were done in technical triplicates.

SLAM-seq

Cell were treated for 6 h with auxin and then with 800 μM 4sU (Sigma-Aldrich) for 0, 1, 2 or 4 h. Then, cells were harvested in RLT buffer and RNA was extracted using RNEasy kit (QIAGEN) with on-column DNAase digestion (QIAGEN). ERCC RNA Spike-in Mix 1 (Thermo Fisher Scientific) was added to each RNA sample in equal amounts, alkylation was carried out using 10 mM iodoacetamide (Thermo Fisher Scientific), and the reaction was quenched with 0.1 M DTT (Thermo Fisher Scientific). Alkylated RNA was purified on MinElute columns (QIAGEN). RNA integrity was verified using standard RNA kit on Fragment Analyzer (Agilent Technologies). For samples passing quality checks, the RNA was subjected to library preparation using QuantSeq kit (Lexogen) for 13 cycles and sequenced for 75 cycles on Illumina NextSeq500.

Cellular growth and apoptosis assays

To measure growth, cells were seeded in 15 cm dishes and treated or not with auxin for 12 h. These cells were then reseeded in a 6-well plate (200,000 cells/well) in triplicate (Day 1). Cells were counted using a CASY cell counter every 48 h, and 200,000 viable cells or all cells from a well were re-seeded into a new well with or without auxin-containing medium.

For the annexin V-PI assay of apoptosis, cells were seeded and, after 24 h, treated or not with auxin for 24 h. Then, the culture medium was collected and trypsinized cells were resuspended in this medium. After spinning down, the cells were washed twice with sterile ice-cold PBS, and resuspended in 100 μl annexin V binding buffer (10 mM HEPES pH 7.4, 140 mM NaCl, 2.5 mM $CaCl_2$) containing 2 μl annexin V, Pacific Blue conjugate. This suspension was incubated in the dark at room temperature for 15 min, after which 400 μl annexin V binding buffer containing 18.5 μM PI was added. Cells were analyzed by flow cytometry on a BD FACSCanto II flow cytometer, and data were analyzed using BD FACSDIVA (v6.1.2) and FlowJo (v8.8.6) software.

For the BrdU-PI cell cycle assay, cells were seeded and, after 24 h, treated or not with auxin for 24 h. Then, cells were labeled for 1 h with 10 μM BrdU (Sigma-Aldrich) in the same medium. The culture medium was collected and trypsinized cells were resuspended in this medium. After spinning down, the cells were washed twice with sterile ice-cold PBS, and fixed overnight in 80% ethanol at −20°C. Fixed cells were washed with ice-cold PBS and resuspended in 2 M HCl, 0.5% Triton X-100 for 30 min at room temperature. For neutralizing, 0.1 M $Na_2B_4O_7$ (pH 8.5) was added and the pellets were resuspended in 100 μl PBS-T (0.5% Tween 20 in PBS) containing 1% BSA and 1 μg FITC-labeled anti-BrdU antibody (BioLegend). Samples were incubated for 30 min at room temperature in the dark. After washing with 1% BSA in PBS-T, cells were resuspended in PBS with RNase A (24 μg/ml) and PI (54 μM). After incubation for 30 min at 37°C, cells were analyzed by flow cytometry as described above.

Proximity ligation assay

Cells were seeded to achieve a density of 20,000−35,000 per 10 mm^2 glass coverslip. They were treated with auxin for 24 h and then fixed with 4% paraformaldehyde. Subsequently, they were permeabilized with 0.3% Triton X-100, treated with blocking solution for 45 min, and then incubated in blocking solution with a primary antibody to γH2AX and another primary antibody to RNAPII with different phosphorylation marks. *In situ* proximity ligation assays (PLA) were done using Duolink kits (Sigma-Aldrich). Briefly, cells were incubated for 1 h at 37°C with PLA probe-labeled anti-rabbit and anti-mouse IgG. Then, ligase solution was added for 30 min at 37°C. Finally, *in situ* PCR amplification was done with Alexa 568-conjugated oligonucleotides for 2.5 h at 37°C followed by counterstaining with Hoechst 33342 (Thermo Fisher Scientific) and Alexa 488-conjugated phalloidin (Thermo Fisher Scientific). Coverslips were mounted on microscope slides using Fluoromount (Sigma-Aldrich), and imaged under a confocal microscope (Leica SP8) with a 60 × objective under glycerol immersion. Images were converted to maximum intensity projections and displayed with brightness and contrast settings for the quantification of individual foci from 50-320 cells per condition across multiple biological replicates. BZ-X800 Keyence All in one microscope was used with 40 × plan achromat objective (air immersion). Quantification of PLA dots was carried out with the analysis module of the Keyence BZ-H4XD software.

Quantitative mass spectrometry

Extraction of chromatin fraction

To enrich for RNAPII-associated proteins, the chromatin-associated fraction was isolated from U2OS cells as described (Aygün et al., 2008). In brief, HA-tagged RPB3 expressing U2OS$^{SPT6\text{-}AID\text{-}C1}$ were seeded 24 h before auxin treatment. SPT6 degradation was induced by adding auxin (500 μM) to the medium 4 h before harvesting. For each condition, 200 million cells were washed twice

with sterile ice-cold PBS and harvested in sterile PBS with phosphatase and protease inhibitor cocktails (Sigma) followed by centrifugation (300 g, 15 min, 4°C). To obtain the cytoplasmic fraction, pellets were dissolved in Extraction Buffer I (10 mM HEPES pH 7.9, 0.34 M sucrose, 3 mM $CaCl_2$, 2 mM magnesium acetate, 0.1 mM EDTA, 0.5% NP-40) with freshly added phosphatase and protease inhibitor cocktails (Sigma) and incubated on a rotating wheel for 20 min at 4°C. Nuclei were isolated by centrifugation (3900 g, 20 min, 4°C) and supernatants were collected as the cytoplasmic fraction. Collected nuclei were washed once with Extraction Buffer I without NP-40. Nuclear extraction was done in Extraction Buffer II (20 mM HEPES pH 7.9, 3 mM EDTA, 10% glycerol, 150 mM potassium acetate, 1.5 mM $MgCl_2$), followed by homogenizing (10 bounces) and a 20 min incubation at 4°C with rotation. After centrifugation (13,000 rpm, 4°C), supernatants were collected as the nucleoplasmic fraction and chromatin was pelleted. Chromatin pellets were treated with nuclease incubation buffer (150 mM HEPES pH 7.9, 1.5 mM $MgCl_2$, 150 mM potassium acetate), homogenized in a homogenizer (30 bounces), and sheared by sonication (4 times, 10 s pulses, 45 s pauses, 20% output). Benzonase (100 units/ml; Novagen) was added and samples were incubated for 40 min at 16°C in a thermoshaker (1400 rpm). Samples were centrifuged (18,000 rpm, 30 min, 4°C). Supernatants were collected as the chromatin fraction and used as input for IP. The unsolubilized chromatin pellet was dissolved in 1x Lämmeli buffer (15 mM Tris pH 6.8, 3% SDS, 0.015% bromophenol blue, 10% glycerol, 1.5 mM 1,4-dithiothreitol) and used to check the efficiency of fractionation.

Immunoprecipitation

IP for the soluble chromatin fraction was carried out by incubating with 80 μL HA-coupled magnetic beads (Thermo Fisher Scientific) with HA-RPB3 as a bait and 200 units benzonase at 4°C for 3 h with rotation. Then, beads were washed thrice with IP washing buffer (20 mM HEPES pH 7.9, 150 mM KCl, 0.5 mM EDTA, 10% glycerol) containing 0.1% Triton X-100, followed by two washes without Triton X-100. Proteins complexes on the beads were eluted in 100 μL 1x LDS Sample Buffer (NuPAGE Thermo Fisher Scientific) by incubating for 30 min at 37°C on a thermoshaker (400 rpm). 50 mM 1,4-dithiothreitol (DTT) was added to the eluates and samples were heated at 95°C for 5 min.

In-solution digestion

Proteins in NuPAGE sample buffer (Invitrogen) were reduced in 50 mM DTT for 10 min at 70°C, and alkylated with 120 mM iodoacetamide for 20 min at room temperature in the dark. Protein was precipitated overnight at −20°C with fourfold volumes of acetone. Pellets were washed four times with acetone at −20°C. Precipitated proteins were dissolved in 100 μl 4 M urea in 100 mM ammonium bicarbonate and digested with 0.25 μg Lys-C (Wako) for 2 h at 30°C followed by an overnight digestion with 0.25 μg trypsin at 37°C. Prior to trypsin digest, samples were diluted to 2 M urea by adding 100 μl 100 mM ammonium bicarbonate. Peptides were desalted using C18 Stage Tips. Each Stage Tip was prepared with three discs of C18 Empore SPE Discs (3M) in a 200 μl pipet tip. Peptides were eluted with 60% acetonitrile in 0.3% formic acid, dried in a vacuum concentrator (Eppendorf), and stored at −20°C. Peptides were dissolved in 2% acetonitrile, 0.1% formic acid prior to nanoLC-MS/MS analysis (Rappsilber et al., 2003).

NanoLC-MS/MS analysis

NanoLC-MS/MS analyses were performed on an Orbitrap Fusion (Thermo Scientific) equipped with a PicoView Ion Source (New Objective) and coupled to an EASY-nLC 1000 (Thermo Scientific). Peptides were loaded on capillary columns (PicoFrit, 30 cm x 150 μm ID, New Objective) self-packed with ReproSil-Pur 120 C18-AQ, 1.9 μm (Dr. Maisch) and separated with a 120-minute linear gradient from 3% to 30% acetonitrile and 0.1% formic acid at a flow rate of 500 nl/min.

Both MS and MS/MS scans were acquired in the Orbitrap analyzer with a resolution of 60,000 for MS scans and 7,500 for MS/MS scans. HCD fragmentation with 35% normalized collision energy was applied. A Top Speed data-dependent MS/MS method with a fixed cycle time of 3 s was used. Dynamic exclusion was applied with a repeat count of 1 and an exclusion duration of 90 s; singly charged precursors were excluded from selection. Minimum signal threshold for precursor selection was set to 50,000. Predictive AGC was used with AGC a target value of 2e5 for MS scans and 5e4 for MS/MS scans. EASY-IC was used for internal calibration.

MS data analysis

Raw MS data files were analyzed with MaxQuant version 1.6.2.2 (Cox and Mann, 2008). Database searching was performed with Andromeda, which is integrated in the utilized version of MaxQuant. The search was performed against the UniProt human reference proteome database (download date: 2020-08). Additionally, a database containing common contaminants was used. The search was performed with tryptic cleavage specificity with 3 allowed miscleavages. Protein identification was under control of the false-discovery rate (FDR; < 1% FDR on protein and PSM level). In addition to MaxQuant default settings, the search was performed with the following variable modifications: protein N-terminal acetylation, Gln to pyro-Glu formation (N-term. Gln), and oxidation (Met). Carbamidomethyl (Cys) was set as fixed modification. Further data analysis was performed using R scripts developed in-house. LFQ intensities were used for protein quantitation. Proteins with less than two razor/unique peptides were removed. Missing LFQ intensities in the control samples were imputed with values close to the baseline. Data imputation was performed with values from a standard normal distribution with a mean of the 5% quantile of the combined log_{10}-transformed LFQ intensities and a standard deviation of 0.1. For the identification of significantly enriched proteins, median log_2 transformed protein ratios were calculated from the three replicate experiments and boxplot outliers were identified in intensity bins of at least 300 proteins. Log_2 transformed protein ratios of sample versus control with values outside a 1.5x (significance 1) or 3x (significance 2) interquartile range (IQR), respectively, were considered as significantly enriched in the individual replicates. The RNAPII interactome was defined as the proteins with ‘logFC.limma.minus_vs_untag’ > 2.0 AND ‘adj.P.Val.limma.minus_vs_untag’ < 0.05 and with values in at least 2 replicate pairs (auxin and control), N = 144.

CellPress
OPEN ACCESS

QUANTIFICATION AND STATISTICAL ANALYSIS

Transcription readthrough score calculation

To avoid artifacts originating from low expression and length of genes, the following criteria were applied: (a) genes should be longer than 5 kb, and (b) the sum of read densities over 150 bp sized non-overlapping bins in the PAS-5 kb to PAS+5kb should be > 4. A total of 7562 genes met these criteria and used throughout this study (unless mentioned otherwise) as the set of expressed genes in the U2OS cell line. For genes with multiple TSSs, the longest gene unit was considered.

Sequencing depth-normalized BAM files from 4sU-seq were cleaned of rRNA, exonic and blacklisted regions (Amemiya et al., 2019). Only reads aligning on the sense strand were considered. The data for all expressed genes in the "pre-PAS window" and "readthrough window" were selected and divided into 150 bp wide bins as described (Shen et al., 2014). For ChIP-seq, these windows ranged from PAS-3 kb to PAS and from PAS+5 kb to PAS+15 kb, respectively. For 4sU-seq, they ranged from PAS-5 kb to PAS and PAS+5 kb to PAS+15 kb. The reads in the two windows were summed, and $\log_2$ fold changes were calculated for all expressed genes. This fold change was termed the "readthrough score," and it is shown for all expressed genes in Table S1.

Transcription completion score calculation

Sequencing depth-normalized BAM files from 4sU-seq were cleaned of rRNA, exonic and blacklisted regions (Amemiya et al., 2019). The data were divided into 150 bp wide bins as described (Shen et al., 2014) across all 7,562 expressed genes normalized for length. The values across bins in the first and last 15% of the gene body ("proximal window" and "distal window," respectively) were summed, and $\log_2$ fold changes were calculated for all expressed genes. This fold change was termed the "completion score," and it is shown for all expressed genes in Table S1.

Completion scores were used to generate z-scores, by subtracting the mean and dividing by standard deviation of the anti-log of the completion scores. Z-scores were displayed in a heatmap using *pheatmap* package in R (https://cran.r-project.org/web/packages/pheatmap/index.html).

γH2AX read density comparison

Reads falling in the promoter (TSS – 1 kb to TSS + 2 kb) and readthrough regions (PAS + 5kb) were calculated from spike-normalized BAM files of γH2AX ChIP-Rx were using coverageBed in Bedtools suite after their conversion into respective BED files.

Antisense and PROMPT expression analyses

Stranded metagene plots were prepared by suppressing reads mapping to forward strands for antisense reads and by suppressing reads mapping to reverse strands for sense reads. This analysis was done using –*opposite* and –*same* flags in *ngs.plot.r.*

Expression comparison for PROMPTs was carried out over 1052 PROMPTs annotated earlier (Schlackow et al., 2017). The 4sU-seq reads falling within the annotated PROMPT regions were subjected to $\log_2$FC and significance calculation in *limma* package in R, along with robust statistics correction implementation.

Image processing

Images captured from Leica SP8 microscope were opened in ImageJ and the channels were separated (Schneider et al., 2012). For the PLA channel, a maximum intensity projection was carried out and a Gaussian blur filter (sigma = 0.8 for quantification and 1.2 for the images displayed) was applied to the resulting images. Brightness and contrast correction was then applied over same values in control and auxin-treated cells (1400-2000 minima to 8000-10000 maxima), and merged with Hoechst and phalloidin staining channels.

Peak calling and visualization

Peak calling for reads from siSPT6-treated samples was carried out using the *callpeak* functionality of MACS2 (Zhang et al., 2008) (–*keep-dup* 20 –*pvalue* 1e-4). Peaks were annotated within regions using Bedtools, with definitions as per the UCSC Table Browser for genomic regions or publications (lncRNA (Hon et al., 2017), eRNA (Andersson et al., 2014)). Peaks were ensured to fall only within a single category.

Motif search and enrichment analysis

DNA sequences within peaks called from siSPT6 were analyzed with MEME suite tools (Bailey et al., 2009), such as MEME-Chip and DREME analysis against 4 non-overlapping databases, namely JASPAR2018_POLII, JASPAR_CORE_REDUNDANT_2016_vertebrates, jolma2013 and HOCOMOCOv11_core_HUMAN_mono_meme_format. All outputs were subjected to Centrimo, FIMO and TOMTOM in order to align to the motifs found earlier. The occurrences of motifs in peak regions (siSPT6 or same number of random regions) from FIMO against JASPAR2018_POLII were used to compare RNAPII binding sites.

Metagene plots

Normalized BAM files were used to generate metagene plots using NGSplots v2.41.3, with normalization to library size restricted wherever spike-normalized BAM files were used (Shen et al., 2014). Stranded metagene were prepared by suppressing reads

mapping to forward strands for antisense reads and by suppressing reads mapping to reverse strands for sense reads; this work was done using *–opposite* and *–same* flags in *ngs.plot.r*. Alternatively, matrix generation was carried out in the same way as for heatmaps. The resulting matrix was then converted to a density plot via*–plotprofile* tool in deeptools (Ramírez et al., 2016). We used the positions of TSS and PAS from UCSC Table Browser based on the GRCh37/hg19 human genome assembly and selected genes annotated in the NCBI RefSeq database (O'Leary et al., 2016).

Heatmaps

Normalized BAM files were used to generate relative heatmaps using DeepTools v3.3.0, with reads first converted to matrices with a step size of 5 bp, and $\log_2$ relative scale of z-scores, generated for control and treated conditions in the same matrix. We used the positions of TSS and PAS from UCSC Table Browser based on the GRCh37/hg19 human genome assembly and selected genes annotated in the NCBI RefSeq database (O'Leary et al., 2016).

To generate a p-value heatmap, we used the unpaired two-sided Wilcoxon test on all genes from which the average density was calculated over each bin. In case of replicates, the largest p-value among the replicates was chosen. If the p-value was < 0.001, it was replaced with 0.001 and the $-\log_{10}$ was calculated. Finally, the values were plotted as heatmap using *pheatmap* package in R.

Volcano plots

Volcano plots were made based on the fold changes and p-values derived from the Bayesian test with robust correction on $\log_2$ reads (or scores) using *limma* package in R.

LogFC calculations between auxin-treated and control samples for MS data

To calculate the $\log_2$FC between auxin and control conditions, the values for each replicate were normalized to the average value of the bait, POLR2C (HA-RPB3), for both conditions. (using *limma* package, the $\log_2$FC was calculated with Bayesian fit). RNAPII interacting proteins that significantly changed their interaction upon SPT6 depletion were defined as those having a $\log_2$FC < -0.5, $p < 0.05$.

ODE model of transcription

To simulate RNAPII occupancy and the 4sU-seq and DRB-4sU-seq signals, we first discretized a hypothetical gene with length 80 kb into non-overlapping 1 kb windows. The parameters that determine the evolution of RNAPII occupancy over time are:

- The average time p between successful initiation events (where successful means that a polymerase proceeds into the elongation phase)
- The elongation rate e in kilobases per minute
- The processivity defect d in percent per kilobase (i.e., the percentage of polymerases that stop transcribing within each window)

For each window, we defined the variables o_i and b_i as the number of polymerases having their nascent RNA molecule not labeled (o_i) and labeled (b_i) with 4sU, respectively. The changes in o_i over time without simulating 4sU labeling are described by the following system of ordinary differential equations (ODEs):

$$\frac{do_i}{dt} = \begin{cases} \frac{1}{p} - (e + d \cdot e) \cdot o_1 & \text{for } i = 1 \\ e \cdot o_{i-1} - (e + d \cdot e) \cdot o_i & \text{for } i > 1 \end{cases} \quad \#$$

To simulate the changes in o_i and b_i after the onset of 4sU labeling, we switch from these ODEs (called no4sU mode) to the following system of ODEs (called 4sU mode):

$$\frac{do_i}{dt} = -(e + d \cdot e) \cdot o_i \frac{db_i}{dt} = \begin{cases} \frac{1}{p} - (e + d \cdot e) \cdot b_1 & \text{for } i = 1 \\ e \cdot (o_{i-1} + b_{i-1}) - (e + d \cdot e) \cdot b_i & \text{for } i > 1 \end{cases}$$

We started all simulations by setting all $o_i = 0$ and $b_i = 0$. Except in simulations of DRB experiments, we let the system equilibrate by running the simulation in no4sU mode for 1,000 min. Then, we switched to the 4sU mode for 15 min (4sU labeling time). Finally, we obtained the ChIP-seq profile by summing $o + b$, and the 4sU-seq profile by computing the cumulative sum over b, i.e., the ith element of the 4sU-seq profile is $\sum_{j=i}^{I} b_j$.

For the DRB simulation, we ran the 4sU mode for 20 min without first letting the system equilibrate, and computed the DRB-4sU-seq signal in the same manner as the 4sU-seq signal above.

CellPress
OPEN ACCESS

The parameters used for the figures were:

	p	*e*	*d*
Unperturbed	2 min	3 kb min^{-1}	0% kb^{-1}
Slow	2 min	**1.5 kb min^{-1}**	0% kb^{-1}
Unprocessive	2 min	3 kb min^{-1}	**2% kb^{-1}**

Definition of transcription units

For the LFC analysis, we defined a transcription unit (TU) as consisting of a transcription start site (TSS), a polyadenylation site (PAS), and a number of introns. We started with all transcripts that are annotated as *protein_coding* or *lincRNA* from the latest release in the Ensembl database for the genome assembly hg19.

To define the TSS, we considered the combined CAGE peaks from the FANTOM5 project in addition to all 5′ ends of the Ensembl transcripts. From these, we removed all TSSs that explained less than 50% of the intronic 4sU reads of the gene (i.e., TSS with more than 50% of the reads in the gene upstream). For each remaining candidate TSS *i*, we computed the average coverage of 4sU reads (number of reads / intronic length; only reads that did not overlap with an Ensembl exon, clone C1 without auxin treatment) downstream (d_i) and upstream (u_i) of *i*. We then selected the candidate with maximal coverage increment d_i-u_i.

To define the PAS, we utilized our Quant-seq based SLAM-seq data. Quant-seq libraries are composed of read clusters directly upstream of poly-A tails, which enable the identification of PAS. We first computed the SLAM-seq fragmentation profile by counting the number of SLAM-seq reads (after pooling all samples without auxin treatment) starting at each position within the gene. This profile was smoothed with a Gaussian kernel (bandwidth = 20). We then identified the 3′ most position *p* where the smoothed profile was > 1% of the maximal peak in the profile. If *p* was < 150 nt upstream of an Ensembl-annotated PAS, we selected the annotated PAS. Otherwise we defined the PAS as *p+100* (which was the average distance to annotated PAS). To define the introns, we excluded the union of all Ensembl-annotated exons between TSS and PAS.

LFC regression in gene bodies

For LFC regression on 4sU-seq and ChIP-seq data, we first divided each TU into non-overlapping 1 kb windows, and counted the reads for each. For the 4sU-seq data, we pooled all three replicates of clone C1, considered all reads mapping to the same strand as the TU, and flagged all windows overlapping an exon of the TU as missing values. For the ChIP-seq data, we considered the reads from clone C1 mapping to any strand. We performed background correction for the ChIP-seq read counts by subtracting the read counts from the input sample. Spike-normalized read counts were used throughout this analysis.

For each experiment (4sU, RNAPII ChIP, pS2-RNAPII ChIP) and each TU, we considered two vectors *a* and *c* corresponding to the read counts in 1 kb windows in auxin-treated samples (a) and control samples (c). The log likelihood *f* of the local $\log_2$ fold change l_i in window *i* is:

$$f(l_i) = \log\left(dlfc\left(l_i; \frac{a_i + p_a}{d} + 1, \frac{c_i + p_c}{d} + 1\right)\right)$$

Here, *dlfc* is the density function of the log fold change (Erhard and Zimmer, 2015):

$$dlfc(x, \alpha, \beta) = \frac{2^{x \cdot \alpha} \cdot \log 2}{Beta(\alpha, \beta) \cdot (1 + 2^x)^{\alpha + \beta}}$$

The pseudocounts were set to small values $p_a = \frac{s_a}{s_a + s_c}$ and $p_c = \frac{s_c}{s_a + s_c}$ with $s_a = \sum a_i$ and $s_c = \sum c_i$ corresponding to a weak prior on l_i reflecting the average log fold change (Erhard, 2018). *d* is the downsampling factor for modeling overdispersion.

To model the observed linear decline of local fold changes, we defined the function

$$l(i) = o + s \cdot \min(i, b)$$

This is a continuous function that is linear (with intercept *o* and slope *s*) for $i < b$, and constant for $i \geq b$. We fitted the parameters *o* ($\log_2$ fold change at TSS), *s* (slope of the decline), *b* (turning point from the linear to the constant part of *l*), and the additional nuisance parameter *d* (downsampling factor) by numerically maximizing the total log likelihood $\sum f(l(i))$ over all windows *i* that are not flagged as missing values. We estimated confidence intervals by the quadratic approximation of the log likelihood function using the numerically computed Hessian matrix.

Negative binomial regression in the termination zone

To analyze the decline in 4sU signal downstream of the PAS, we first partitioned 5 kb upstream and 25 kb downstream of each PAS into non-overlapping 250 nt windows, and counted reads for each. We pooled all three replicates of clone C1, considered all reads

mapping to the same strand as the TU, and flagged all windows overlapping an exon of the TU as missing values. Furthermore, if the TU was shorter than 5 kb, all windows that were upstream of it were also flagged as missing values. If there was another TU downstream within 25 kb on the same strand, all windows downstream of its TSS were also flagged as missing values. Spike-normalized read counts were used throughout this analysis.

For each TU, we first computed the mean m and standard deviation s of the upstream windows. If this was not possible due to missing values, we used the first five downstream windows. For negative binomial regression, we considered the vector a of the read counts downstream of PAS. To mitigate the effects of noise and spurious downstream peaks in the 4sU-seq data, we identified all cases of four consecutive windows with < 1% of the read density at PAS, i.e., if $a_i < 0.01 \cdot m$, and flagged all windows downstream as missing values. The log likelihood f of the 4sU signal s_i in window i is:

$$f(s_i) = \log(dnb(a_i;\ s_i, d))$$

Here, dnb is the density function of the negative binomial distribution:

$$dnb(x; n, d) = \binom{x+d-1}{x}\left(\frac{d}{d+n}\right)^d\left(\frac{n}{d+n}\right)^x$$

The parameter $d > 0$ is used to model overdispersion. To model the observed behavior of the read counts downstream of PAS, we defined the function

$$s(i) = o \cdot e^{-ri}$$

This is an exponential decay function that starts with level o at $i = 0$, and then approaches 0 with rate r. We fitted the parameters o (level at PAS), r (exponential decline of 4sU signal), and the additional nuisance parameter d (overdispersion factor) by numerically maximizing the total log likelihood $\sum f(l(i)) + p(o) \cdot n$ with prior p over all windows i that were not flagged as missing values. As prior function on o we used the log density of a Gaussian distribution with mean and standard deviation set to the above-defined m and s. We set equal weights to the prior and the log likelihood by multiplying the prior with the total number of non-missing values n in a. We estimated the confidence intervals by the quadratic approximation of the log likelihood function using the numerically computed Hessian matrix.

Negative binomial regression for estimating the wavefront in DRB-4sU-seq data

To analyze DRB-4sU-seq data, we first fitted a background model for each sample. This was done by extracting the density d (number of reads / intronic length) of intronic reads in the last 5 kb of all genes longer than 150 kb for each DRB-4sU-seq data. In addition, we determined the spike-normalized reads per kilobase r for each TU for the pooled 4sU-seq data (in control or auxin-treated samples; only clone C1; all replicates pooled). We then performed quantile regression (using the rq function from the quantreg R package) with log d as dependent variable and log r as independent variable, to obtain the fits for the 5% and 95% percentiles of each DRB-4sU-seq sample. This enabled us to robustly predict the range of potential background levels for any gene dependent on its expression strength.

For each of the six DRB-4sU-seq samples (10 min, 20 min, 30 min; auxin-treated and control) and each TU, we considered the vector a corresponding to the read counts in 1 kb windows. All windows overlapping an exon were flagged as missing values. Furthermore, to mitigate the effect of the peak we frequently observed at the TSS for DRB-4sU-seq data, we flagged the first 5 kb as missing values. We considered the same log likelihood function f based on the negative binomial distribution as for the termination zone. To model the wavefront, we defined the function

$$w(i) = \left(o + \frac{bg \cdot 2^{-r \cdot b} - o}{b}\min(i, b)\right) \cdot 2^{r \cdot \min(i,b)}$$

For $r = 0$, this is the same function as the one defined for LFC regression, i.e., a continuous function that is linear for $i < b$, starting at o for $i = 0$ and reaching background level bg for $i = b$, and that is constant for $i \geq b$ with $w(i) = bg$. For $r < 0$, the linear part becomes the product of a linear function and an exponential decay function with rate r. The linear part models the triangular shape expected for DRB-4sU data, and the exponential decay models the processivity defect. In both cases, the position b is the wavefront.

For auxin-treated samples, we set $r = s$, where s is the slope of the LFC regression fit for the 4sU-seq data. For control samples, we set $r = 0$. We fitted the parameters o (level at the TSS), b (wavefront), the background level bg, and the additional nuisance parameter d (overdispersion factor) by numerically maximizing the total log likelihood $\sum f(w(i)) + p(bg)$ with prior p over all windows i that are not flagged as missing values. As prior function on bg we used the log density of a Gaussian distribution with 5% and 95% percentiles matching the values predicted by the quantile regression model defined above. We estimated confidence intervals by the quadratic approximation of the log likelihood function using the numerically computed Hessian matrix.

Analysis of SLAM-seq data

We used the GRAND-SLAM pipeline (version 2.0.5g) (Jürges et al., 2018) to process SLAM-seq data. The json config file necessary to set up the pipeline is available on Zenodo. Briefly, 10 nt (6 nt unique molecular identifier [UMI] + 4 nt spacer) were trimmed from the

5′ ends of reads (FastqFilter program from the GRAND-SLAM pipeline), and the sequencing adaptor (AGATCGGAAGAGCACACGT CTGAACTCCAGTCA) was trimmed from the 3′ end using reaper from the Kraken package (version 13-274). Next, bowtie2 (version 2.3.0) (Langmead and Salzberg, 2012) with default parameters was used to discard reads mapping to rRNA (GenBank identifier U13369.1) and to verify the absence of mycoplasma contamination. STAR (version 2.5.3a) was used to map all remaining reads with length at least 18 nt against a combined index of the human genome (hg19, Ensembl 87) and ERCC92 spike-ins (parameters:–outFilterMismatchNmax 20–outFilterScoreMinOverLread 0.4–outFilterMatchNminOverLread 0.4–alignEndsType Extend5pOfReads12–outSAMattributes nM MD NH). Finally, all reads mapping at the same genomic location sharing the same UMI were collapsed, and only mismatches that occurred in the majority of these reads were retained (DedupUMI program of the GRAND-SLAM pipeline).

The GRAND-SLAM program was run with parameter –trim 15 against a combined index of the TUs defined above and the ERCC92 spike-ins, to count reads and to estimate the new-to-total RNA ratio (NTR) for each sample and each TU. Read counts per sample were normalized by dividing by the size factors (estimateSizeFactorsForMatrix from the DESeq2 R package) computed from either the total number of ERCC92 mapped reads, the total number of TU mapped reads. Since the read counts all represent the steady state, the variance for the four time points of 4sU labeling (0, 1, 2 and 4 h) in control samples reflects the precision of the spike-in normalization. We noticed that the variance among the 12 samples (four time points, each with three replicates) was greater than the differences to the auxin-treated samples. Thus, the effect of auxin on total RNA levels was smaller than the measurement uncertainty of the spike-ins. Therefore, we continued with TU size factor-normalized counts. Of note, for these, the overall fit of the kinetic model was better than for the spike-normalized reads.

Kinetic modeling of SLAM-seq data

We modeled the change in total RNA levels a at time t of a TU with transcription rate σ and degradation rate δ by the following differential equation:

$$\frac{da}{dt} = \sigma - \delta \cdot a(t)$$

With total RNA level $a(0) = a_0$ at time $t = 0$, this has the following closed-form solution:

$$a(t) = \left(a_0 - \frac{\sigma}{\delta}\right)e^{-t\delta} + \frac{\sigma}{\delta}$$

Thus, the following equations for new and old RNA levels in auxin-treated or control samples can be derived:

$$f^{old,aux}(t,\sigma,\delta) = a_0 e^{-t\delta}$$

$$f^{old,ctrl}(t,\sigma,\delta) = \frac{\sigma}{\delta}e^{-t\delta}$$

$$f^{new,aux}(t,\sigma,\delta) = \frac{\sigma}{\delta}\cdot\left(1 - e^{-t\delta}\right) = f^{new,ctrl}(t,\sigma,\delta)$$

Note that, under non-steady-state conditions, old RNA levels depend on the level at time 0, a_0. Control samples are in steady state, i.e., new and old RNA levels are modeled by $f^{new,ctrl}$ and $f^{old,ctrl}$, respectively, whereas auxin-treated samples are not in steady state and are modeled by $f^{new,aux}$ and $f^{old,ctrl}$.

For each of the 24 samples, we computed the estimated new and old RNA levels $g_{l,t,a,r}$, where $l \in \{old, new\}$ indicates old or new, $t \in \{0, 1, 2, 4\}$ is the time period of 4sU labeling, $a \in \{aux, ctrl\}$ whether the sample was treated with auxin, and $r \in \{1, 2, 3\}$ the replicate, per TU by multiplying the normalized read count by NTR and 1-NTR, respectively. We fitted the transcription and degradation rates σ_c and δ_c without auxin treatment by minimizing the residuals $g_{l,t,ctrl,r} - f^{l,ctrl}(t,\sigma_c,\delta_c)$ (all with equal weights) using the nls.lm function from the minpack.lm R package. Similarly, we fitted the transcription and degradation rates σ_a and δ_a with auxin treatment by minimizing the residuals $g_{l,t,aux,r} - f^{l,aux}(t,\sigma_c,\delta_c)$ (all with equal weights), while setting $a_0 = \frac{1}{3}\sum_{r=1}^{3} g_{old,0,aux,r}$ to the average of the RNA levels at time 0.

We noticed that genes with short-lived RNAs appeared to be downregulated in the 4 h 4sU samples compared to the 0 h 4sU samples. This indicates that either prolonged 4sU treatment inhibited transcription or that the high number of T- > C mismatches resulted in fewer mapped reads. In both cases, the estimated old RNA levels are unaffected. Thus, we excluded $g_{new,4,a,r}$ for all a and r from the fit; because we also observed this effect, very weakly, in the 2 h samples, we also excluded $g_{new,4,a,r}$. Thus, degradation rates were estimated using all time points, and the synthesis rates were estimated from the 1 h time point and the degradation rates. We estimated confidence intervals by the quadratic approximation of the log likelihood function using the numerically computed Hessian matrix.

Molecular Cell, Volume *81*

Supplemental information

Targeted protein degradation reveals a direct role of SPT6 in RNAPII elongation and termination

Ashwin Narain, Pranjali Bhandare, Bikash Adhikari, Simone Backes, Martin Eilers, Lars Dölken, Andreas Schlosser, Florian Erhard, Apoorva Baluapuri, and Elmar Wolf

Figure S1

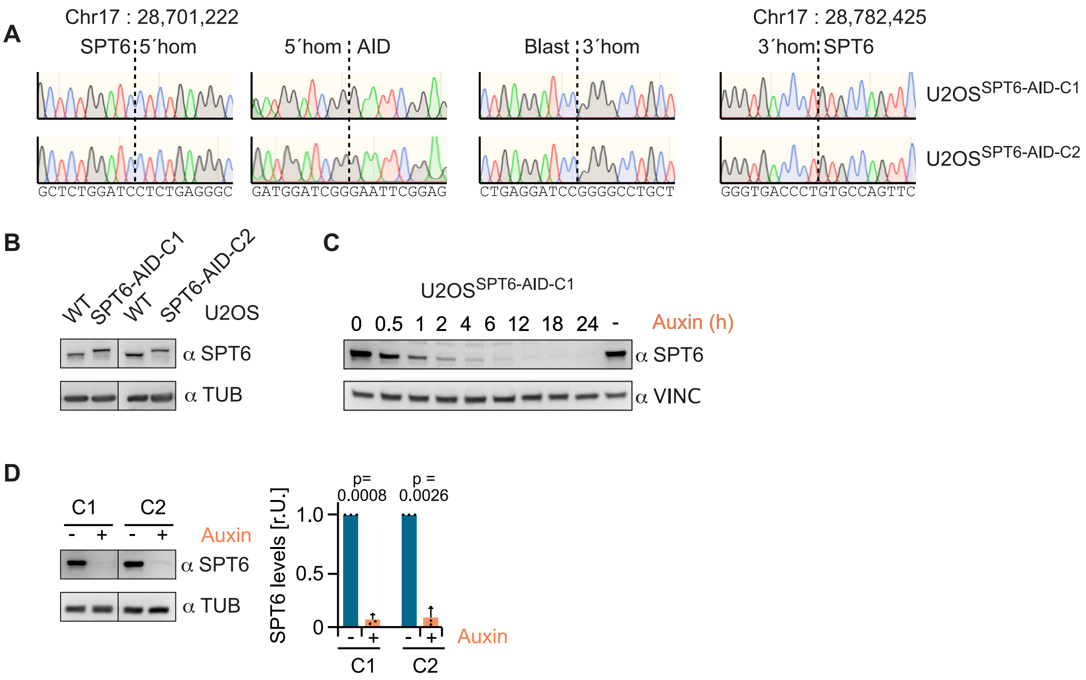

U2OS cells expressing SPT6 with C-terminal auxin-inducible degron. Related to Figure 1. (A) Sanger sequencing electropherogram of the genomic locus of U2OS$^{SPT6\text{-}AID\text{-}C1}$ and U2OS$^{SPT6\text{-}AID\text{-}C2}$ clones. 5'hom, 5' homology arms; 3'hom, 3' homology arms; Blast, blasticidin resistance gene. Genomic locations are indicated. **(B)** Immunoblot of SPT6 in wildtype (WT) U2OS, U2OS$^{SPT6\text{-}AID\text{-}C1}$ and U2OS$^{SPT6\text{-}AID\text{-}C2}$ cells. Both clones were analyzed on separate membranes. TUB, tubulin loading control. **(C)** Immunoblot of SPT6 in U2OS$^{SPT6\text{-}AID\text{-}C1}$ cells treated with auxin for the indicated times. VINC, vinculin loading control. **(D)** Immunoblot (left) of SPT6 in U2OS$^{SPT6\text{-}AID\text{-}C1}$ and U2OS$^{SPT6\text{-}AID\text{-}C2}$ cells treated with auxin (4 h). Both clones were analyzed on separate membranes. TUB, tubulin loading control. Quantification (right) of biological replicates. Values are mean ± SD, n=3. P-values were calculated with a two-tailed unpaired *t* test assuming equal variance.

Figure S2

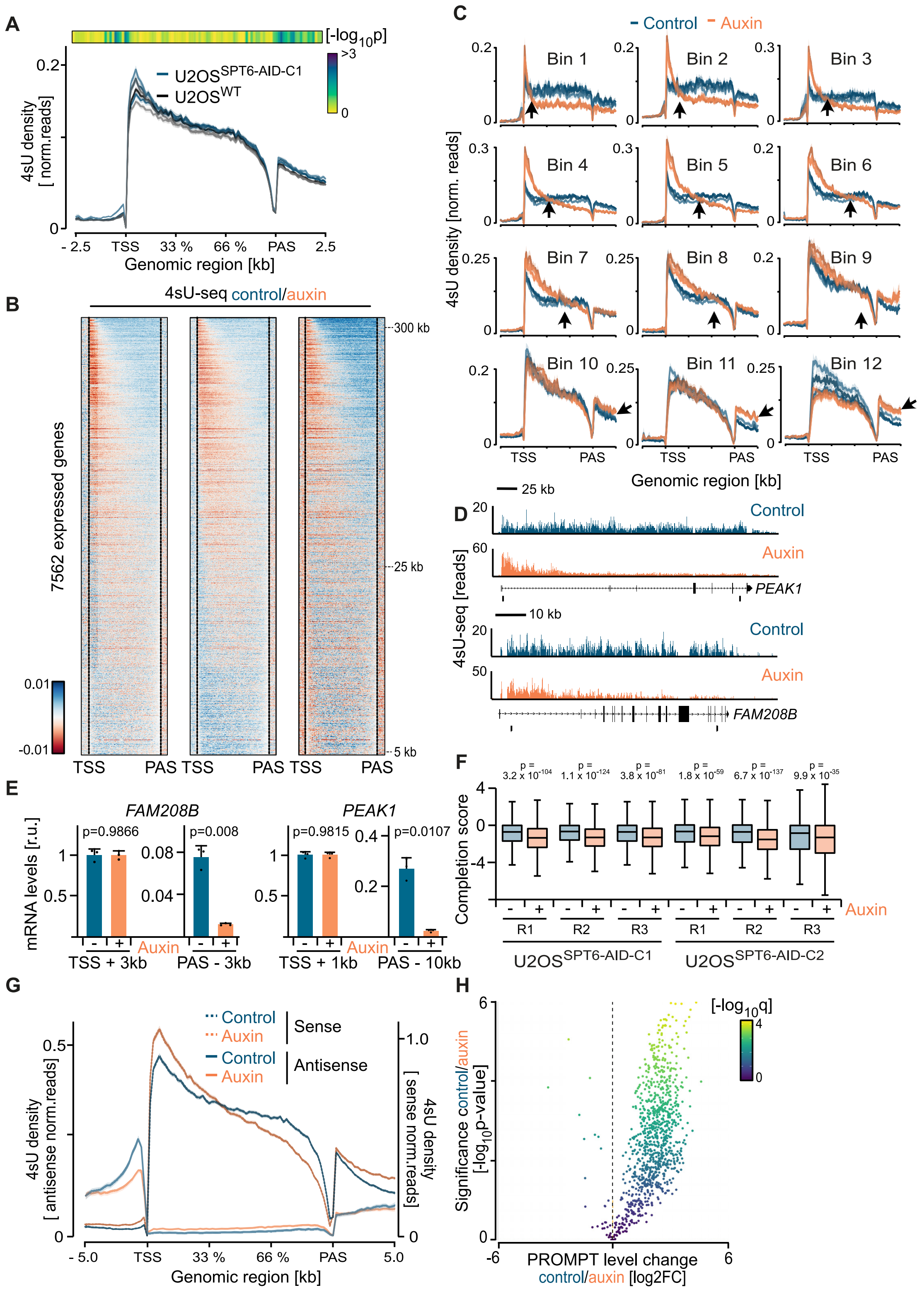

A
[-log10p]
U2OS SPT6-AID-C1
U2OS WT
4sU density [norm.reads]
Genomic region [kb]
B
4sU-seq control/auxin
7562 expressed genes
300 kb
25 kb
5 kb
TSS PAS TSS PAS TSS PAS
C
Control Auxin
Bin 1 Bin 2 Bin 3 Bin 4 Bin 5 Bin 6 Bin 7 Bin 8 Bin 9 Bin 10 Bin 11 Bin 12
4sU density [norm. reads]
Genomic region [kb]
D
25 kb
Control
Auxin
PEAK1
10 kb
FAM208B
4sU-seq [reads]
E
FAM208B
PEAK1
mRNA levels [r.u.]
p=0.9866 p=0.008 p=0.9815 p=0.0107
TSS + 3kb PAS - 3kb TSS + 1kb PAS - 10kb
Auxin
F
Completion score
R1 R2 R3 R1 R2 R3
U2OS SPT6-AID-C1 U2OS SPT6-AID-C2
G
Control Auxin Sense
Control Auxin Antisense
4sU density [antisense norm.reads]
4sU density [sense norm.reads]
Genomic region [kb]
H
[-log10q]
Significance control/auxin [-log10p-value]
PROMPT level change control/auxin [log2FC]

Effect of SPT6 depletion is conserved across replicates. Related to Figure 2. (A) Metagene plot showing the distribution of read density over the gene body from TSS to PAS averaged over 7,562 expressed genes for U2OS^{WT} and untreated U2OS$^{SPT6\text{-}AID\text{-}C1}$ cells for three biological replicates. Shadows around curves indicate SEM values. P-values (two-sided Wilcoxon test) for the difference (auxin/control), calculated from the density values of individual genes (n=7,562) at each genomic location, are shown in a heatmap. **(B)** Relative heatmaps for three replicates showing normalized $\log_2$ fold change between control and auxin-treated U2OS$^{SPT6\text{-}AID\text{-}C1}$ cells from 4sU-seq reads in 7,562 expressed genes sorted by length and scaled to the same length. Orange indicates less reads and blue indicates more reads in control condition. **(C)** Metagene plots of the distribution of read density over the gene body from TSS to PAS, averaged over different bins of genes stratified by length. Shadows around curves indicate SEM values, and arrows indicate the crossover points between curves. Genes were sorted according to length and grouped into bins: Bin 1 contains the longest genes and bin 12 the shortest genes. **(D)** Browser tracks of one replicate from a 4sU-seq experiment for *PEAK1* and *FAM208B* in U2OS$^{SPT6\text{-}AID\text{-}C1}$ cells in the presence or absence of auxin (6 h). Primers for quantitative PCR are indicated below. **(E)** qPCR analysis of RNAPII processivity. Graphs show signals at *PEAK1* and *FAM208B* genes (primers shown in Fig S2D), relative to TSS proximal primers. Values are mean ± SD (n=3). Two-tailed unpaired *t* test assuming equal variance. **(F)** Completion scores for three biological replicates in U2OS$^{SPT6\text{-}AID\text{-}C1}$ and U2OS$^{SPT6\text{-}AID\text{-}C2}$ cells in presence (orange) or absence (blue) of auxin. Unpaired two-sided Wilcoxon test. **(G)** Strand-separated metagene plot showing the distribution of sense and antisense read densities over the gene body from TSS to PAS, averaged over 7,562 expressed genes for U2OS$^{SPT6\text{-}AID\text{-}C1}$ control cells compared to cells treated with auxin (6 h) for reads combined from three biological replicates. **(H)** Volcano plot comparing $\log_2$ fold change and the statistical significance of antisense reads in 1,052 annotated PROMPTs (Schlackow et al., 2017), calculated in control and auxin conditions. The color of individual data points indicates the corresponding PROMPT's adjusted p-value ($-\log_{10}$(qvalue)).

Figure S3

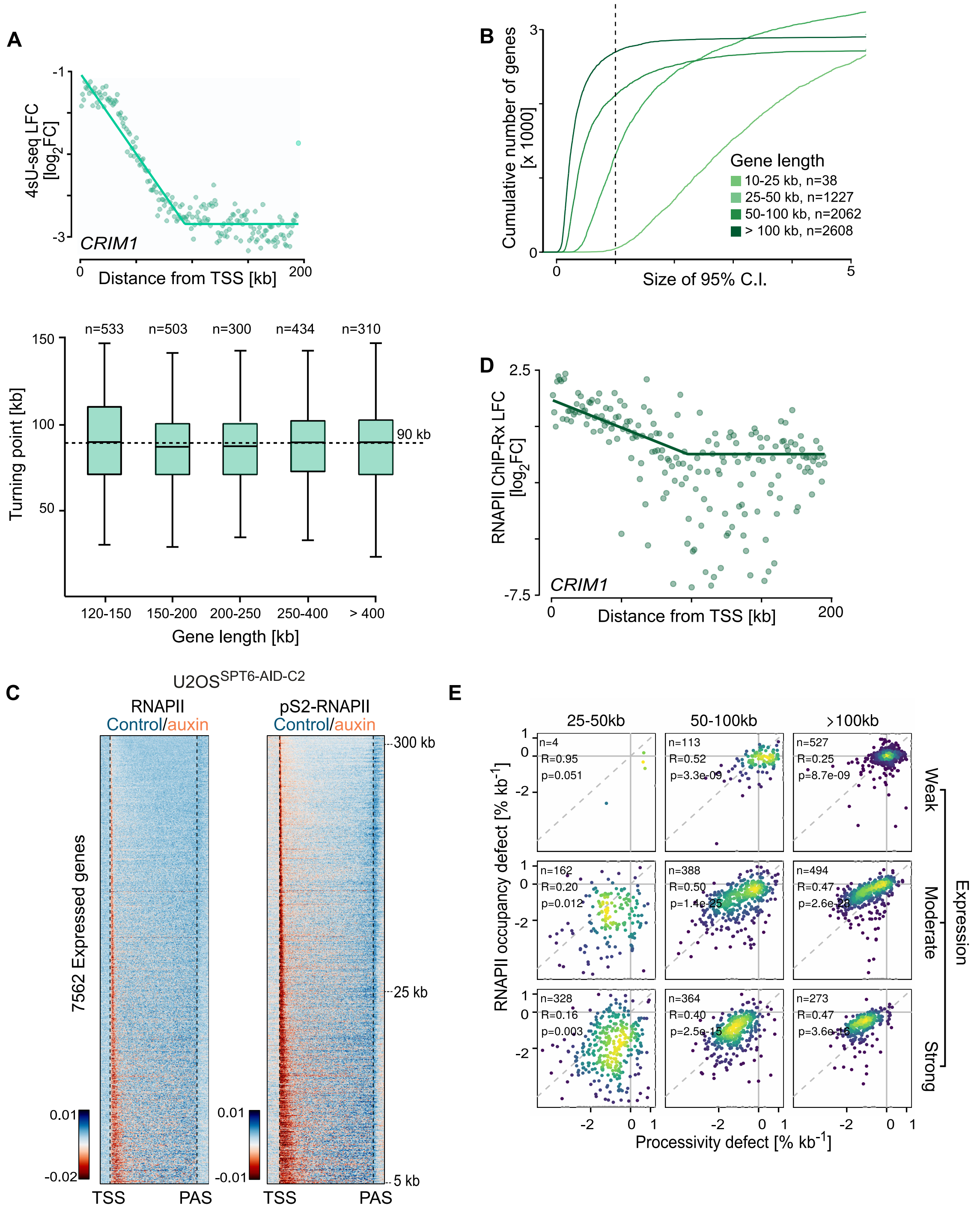

A
4sU-seq LFC [log2FC]
CRIM1
Distance from TSS [kb]
Turning point [kb]
n=533
n=503
n=300
n=434
n=310
90 kb
120-150
150-200
200-250
250-400
> 400
Gene length [kb]
B
Cumulative number of genes [x 1000]
Size of 95% C.I.
Gene length
10-25 kb, n=38
25-50 kb, n=1227
50-100 kb, n=2062
> 100 kb, n=2608
D
RNAPII ChIP-Rx LFC [log2FC]
CRIM1
Distance from TSS [kb]
C
U2OS SPT6-AID-C2
RNAPII
pS2-RNAPII
Control/auxin
7562 Expressed genes
300 kb
25 kb
5 kb
TSS
PAS
E
25-50kb
50-100kb
>100kb
Weak
Moderate
Strong
Expression
RNAPII occupancy defect [% kb-1]
Processivity defect [% kb-1]
n=4
R=0.95
p=0.051
n=113
R=0.52
p=3.3e-09
n=527
R=0.25
p=8.7e-09
n=162
R=0.20
p=0.012
n=388
R=0.50
p=1.4e-25
n=494
R=0.47
p=2.6e-28
n=328
R=0.16
p=0.003
n=364
R=0.40
p=2.5e-15
n=273
R=0.47
p=3.6e-16

Differentiation between elongation rate defects and processivity defects. Related to Figure 3. (A) Top, local fold change (LFC) for example gene *CRIM1*. The plot shows log_2 fold changes in read counts in non-overlapping 1 kb windows for the pooled 4sU-seq replicates in U2OS$^{SPT6\text{-}AID\text{-}C1}$ cells in presence vs. absence of auxin (6 h). Bottom, distributions of turning point positions in LFC fits for 4sU signal, according to gene length. The number of genes per bin is indicated. **(B)** Cumulative distribution of size of the 95% confidence interval (CI) for the processivity defect parameter estimated with LFC regression, for four gene groups according to length. The number of genes in each length-based bin is shown. The size of the 95% CI for the rate of the exponential decline in 4sU signal along the gene body for auxin-treated samples compared to control samples (% kb^{-1}) is shown on the X-axis. **(C)** Relative heatmaps of ChIP-Rx experiments in U2OS$^{SPT6\text{-}AID\text{-}C2}$ cells with antibodies against pS2-RNAPII (right) or RNAPII (left) over 7,562 expressed genes scaled to the same length. Orange indicates less reads and blue indicates more reads in control cells. **(D)** LFC for example gene *CRIM1*. The plot shows log_2 fold change of read counts in non-overlapping 1 kb windows for a RNAPII ChIP-Rx experiment in U2OS$^{SPT6\text{-}AID\text{-}C1}$ cells in presence vs. absence of auxin (6 h). **(E)** Scatter plots correlating processivity defects with RNAPII occupancy for 2,393 genes with accurate LFC fits for the 4sU and ChIP data. Processivity defect is the rate of exponential decline in 4sU signal along the gene body for auxin-treated samples compared to control samples. Y-axis indicates the same parameter for RNAPII ChIP signal. The genes are stratified by expression level (weak, moderate, strong) and length.

Figure S4

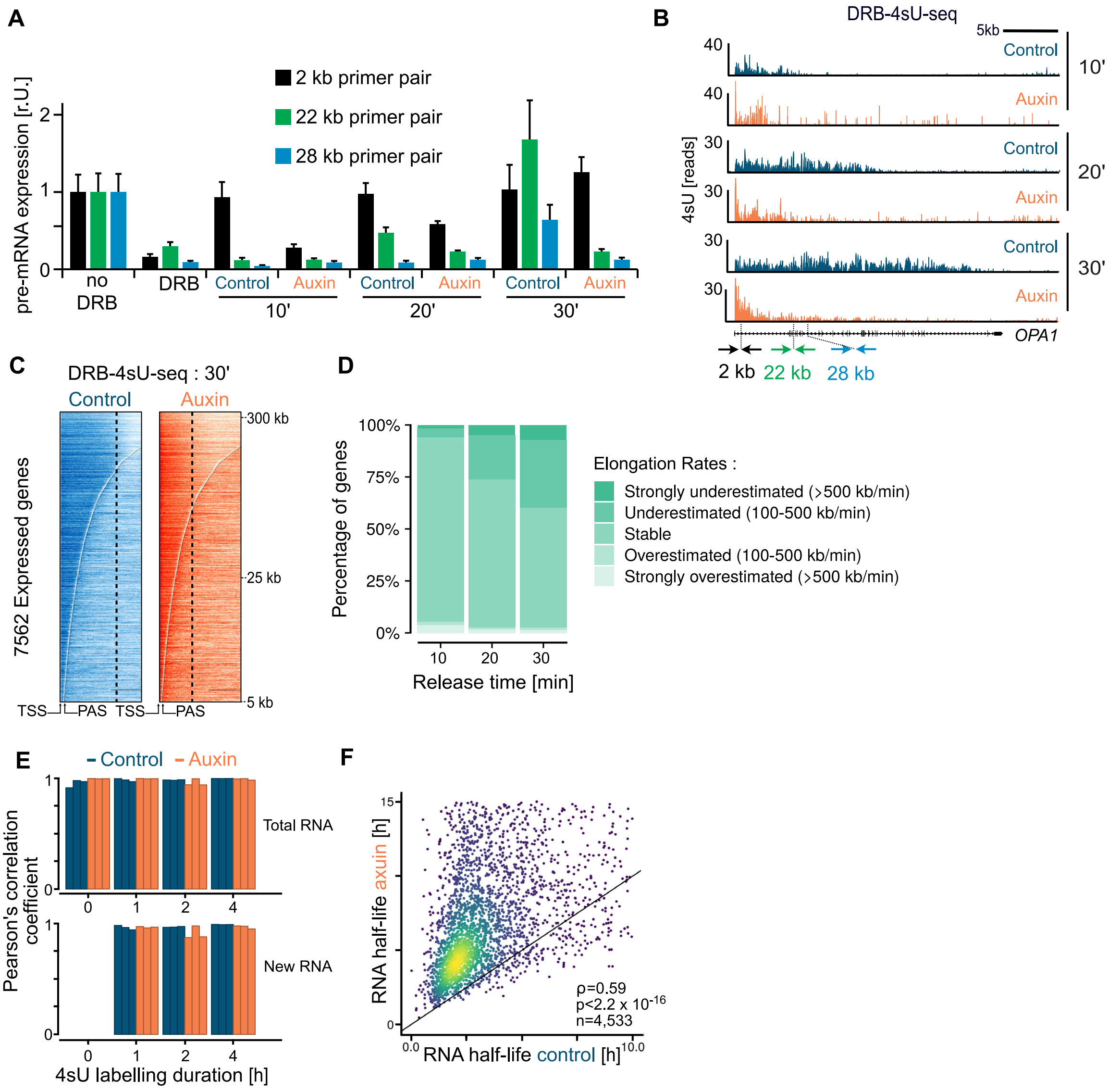
A
2 kb primer pair
22 kb primer pair
28 kb primer pair
pre-mRNA expression [r.U.]
no DRB
DRB
Control
Auxin
10'
20'
30'
B
DRB-4sU-seq
5kb
4sU [reads]
OPA1
2 kb
22 kb
28 kb
C
DRB-4sU-seq : 30'
7562 Expressed genes
300 kb
25 kb
5 kb
TSS
PAS
D
Percentage of genes
Release time [min]
Elongation Rates :
Strongly underestimated (>500 kb/min)
Underestimated (100-500 kb/min)
Stable
Overestimated (100-500 kb/min)
Strongly overestimated (>500 kb/min)
E
Pearson's correlation coefficient
Total RNA
New RNA
4sU labelling duration [h]
F
RNA half-life axuin [h]
RNA half-life control [h]
ρ=0.59
p<2.2 x 10-16
n=4,533

Acute SPT6 depletion leads to processivity loss. Related to Figure 4. (A) Quantification of 4sU-seq reads using RT-qPCR in example gene *OPA1* after DRB inhibition and release. Locations of primers are indicated as the distance from TSS, shown in panel (B). **(B)** Browser tracks showing a DRB-4sU-seq experiment at *OPA1* gene in U2OS$^{SPT6\text{-}AID\text{-}C1}$ cells in presence or absence of auxin (6 h), followed by DRB inhibition and release. Positions of qPCR primers are shown below. **(C)** Heatmaps sorted by gene length for DRB-4sU-seq experiments in U2OS$^{SPT6\text{-}AID\text{-}C1}$ cells in presence or absence of auxin (6 h), followed by DRB inhibition and release for 30 min over 7,562 expressed genes. Dotted lines indicate the visual wavefront. **(D)** Estimation of elongation rates by regression using linear vs. exponential functions in DRB-4sU-seq experiments for auxin-treated samples. The X-axis shows the three samples (10, 20 and 30 min DRB washout). The Y-axis shows the percentages of genes with accurate regression fit, according to how their elongation rates were estimated with the linear function. **(E)** All pairwise correlation coefficients for expression levels of total and newly transcribed RNA at different times among the three replicates of SLAM-seq experiments in U2OS$^{SPT6\text{-}AID\text{-}C1}$ cells incubated in presence or absence of auxin (6 h), followed by 4sU labeling. **(F)** Scatter plot comparing RNA half-lives for 4,533 genes, in control and auxin-treated U2OS$^{SPT6\text{-}AID\text{-}C1}$ cells, estimated by mathematical modeling using SLAM-seq data. Spearman's ρ and p values computed by asymptotic t approximation are indicated.

Figure S5

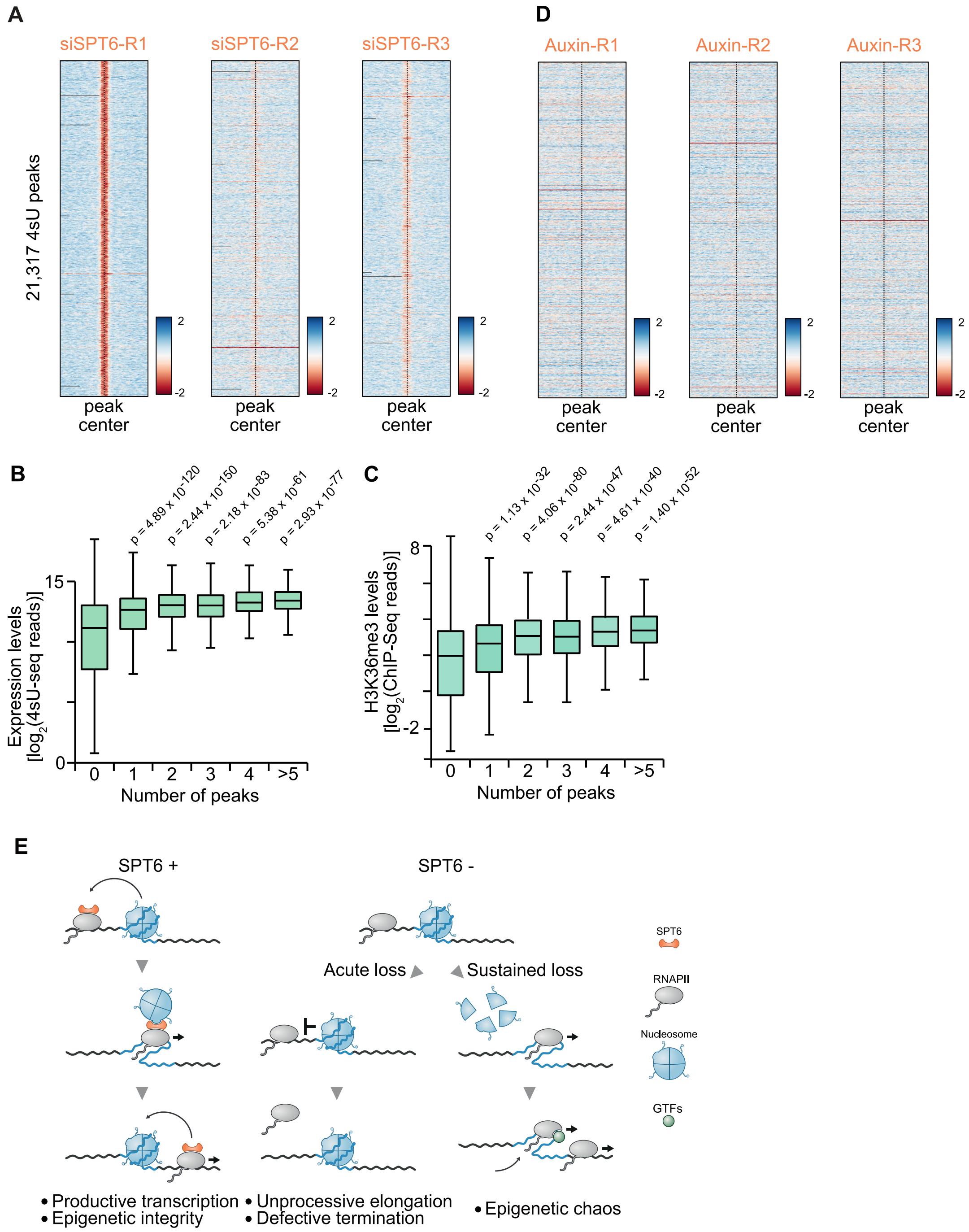

A
siSPT6-R1
siSPT6-R2
siSPT6-R3
21,317 4sU peaks
peak center
D
Auxin-R1
Auxin-R2
Auxin-R3
B
p = 4.89 x 10-120
p = 2.44 x 10-150
p = 2.18 x 10-83
p = 5.38 x 10-61
p = 2.93 x 10-77
Expression levels [log2(4sU-seq reads)]
Number of peaks
C
p = 1.13 x 10-32
p = 4.06 x 10-80
p = 2.44 x 10-47
p = 4.61 x 10-40
p = 1.40 x 10-52
H3K36me3 levels [log2(ChIP-Seq reads)]
Number of peaks
E
SPT6 +
SPT6 -
Acute loss
Sustained loss
SPT6
RNAPII
Nucleosome
GTFs
• Productive transcription
• Epigenetic integrity
• Unprocessive elongation
• Defective termination
• Epigenetic chaos

Cryptic initiation sites on sustained loss of SPT6. Related to Figure 5. (A) Relative heatmaps centered at 21,317 peaks (±2.5 kb) found in siSPT6 condition. Shown are z-scores comparing 4sU-seq reads from siSPT6-treated and siCTR-treated cells (48 h) for three replicates. **(B)** Transcription rates according to the number of 4sU peaks called in gene bodies in siSPT6-treated U2OS cells. Two-sided unpaired Wilcoxon test. **(C)** H3K36 trimethylation levels according to the number of 4sU peaks in gene bodies in siSPT6-treated U2OS cells. H3K36me3 was re-analyzed from a published dataset (Wen et al., 2014). Unpaired two-sided Wilcoxon test. **(D)** Relative heatmaps centered at 21,317 peaks (±2.5 kb) found in siSPT6 condition. Shown are z-scores comparing 4sU reads from auxin-treated (6 h) to control U2OS$^{SPT6\text{-}AID\text{-}C1}$ cells for three replicates. **(E)** Proposed model showing the state of nucleosomes and RNAPII upon acute and sustained SPT6 loss. GTFs, general transcription factors

Figure S6

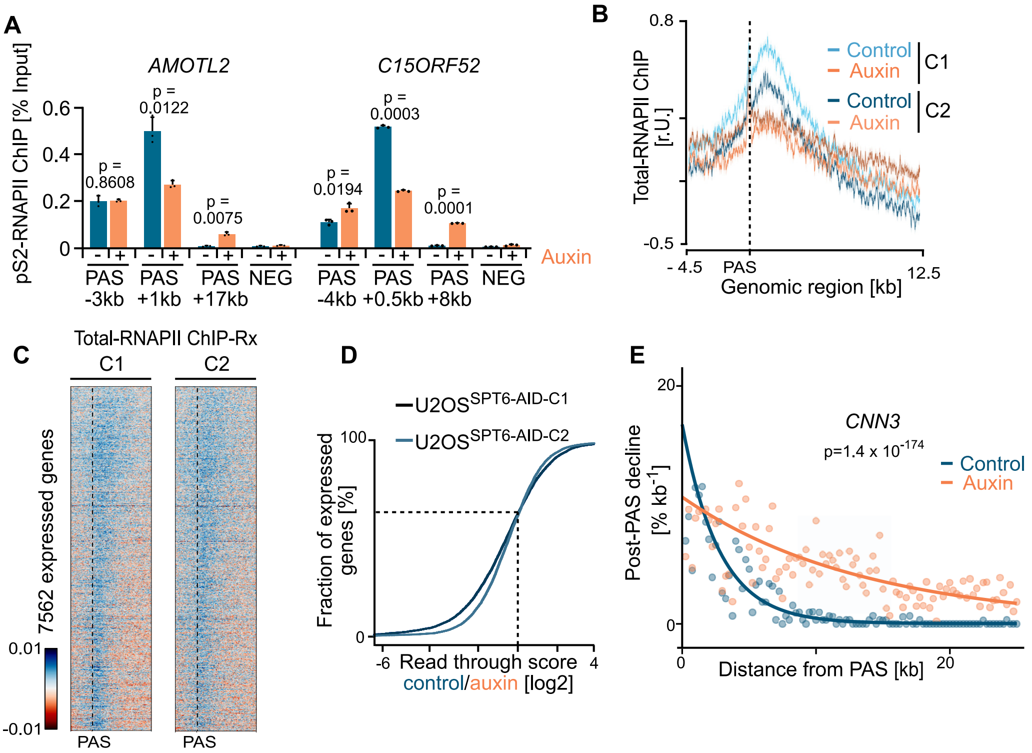

Acute SPT6 depletion causes readthrough at protein-coding genes. Related to Figure 6. **(A)** qPCR validation of pS2-RNAPII ChIP-Rx data. Graph shows occupancy at *AMOTL2* and *C15ORF52* genes (primer pairs indicated in Fig 6F), relative to PAS upstream primers. Values are mean ± SD (n=3). Two-tailed unpaired *t* test assuming equal variance. **(B)** Metagene plots showing distribution of read density from total RNAPII ChIP-Rx positioned around PAS, averaged for 4.5 kb upstream and 12.5 kb downstream in U2OS$^{SPT6\text{-}AID\text{-}C1}$ and U2OS$^{SPT6\text{-}AID\text{-}C2}$ cells in presence or absence of auxin (6 h). Shadows around curves indicate SEM values. **(C)** Relative heatmaps for read density from total RNAPII ChIP-Rx. Shown are z-scores calculated from $\log_2$ fold changes between spike-normalized reads from control and auxin-treated U2OS$^{SPT6\text{-}AID\text{-}C1}$ and U2OS$^{SPT6\text{-}AID\text{-}C2}$ cells for 7,562 expressed genes, sorted by length. Heatmaps are positioned around PAS, showing 4.5 kb upstream and 12.5 kb downstream regions. **(D)** Empirical cumulative distribution of the difference in $\log_2$ fold changes of readthrough scores based on pS2-RNAPII ChIP-Rx from control and auxin-treated cells (6 h) showing the fraction of 7,562 expressed genes. **(E)** Termination zone for example gene *CNN3.* The plots show read counts in non-overlapping 1 kb windows for the pooled 4sU-seq experiments in U2OS$^{SPT6\text{-}AID\text{-}C1}$ cells in presence or absence of auxin (6 h). Y-axis indicates read count values in the pooled 4sU-seq data. The fits of the exponential decline model are indicated as solid lines.

Figure S7

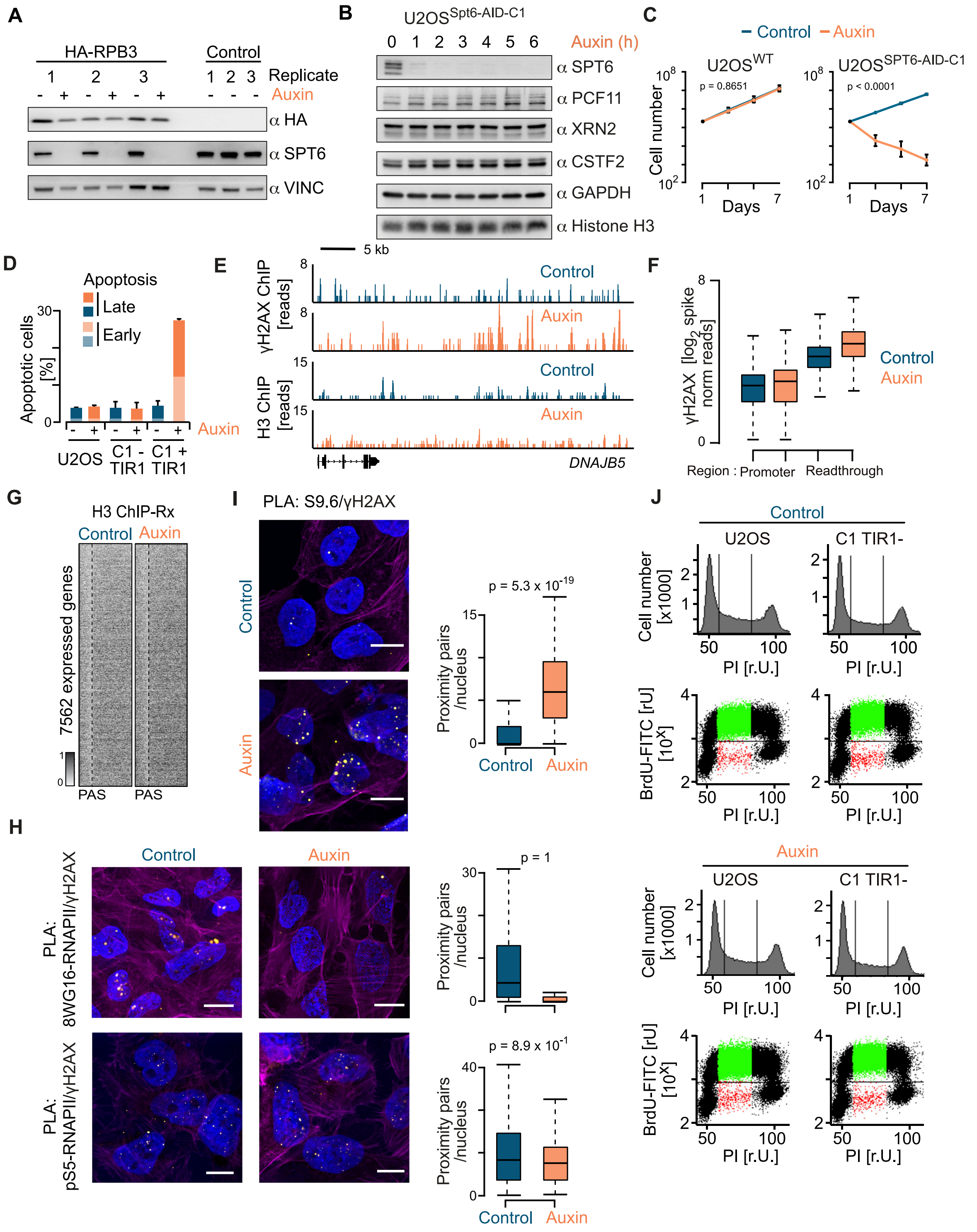

A
HA-RPB3
Control
Replicate
Auxin
α HA
α SPT6
α VINC
B
U2OS Spt6-AID-C1
Auxin (h)
α SPT6
α PCF11
α XRN2
α CSTF2
α GAPDH
α Histone H3
5 kb
C
Control
Auxin
U2OS WT
U2OS SPT6-AID-C1
p = 0.8651
p < 0.0001
Cell number
Days
D
Apoptosis
Late
Early
Apoptotic cells [%]
Auxin
U2OS
C1 TIR1
E
γH2AX ChIP [reads]
H3 ChIP [reads]
Control
Auxin
DNAJB5
F
γH2AX [log2 spike norm reads]
Region : Promoter Readthrough
Control
Auxin
G
H3 ChIP-Rx
Control
Auxin
7562 expressed genes
PAS
I
PLA: S9.6/γH2AX
Control
Auxin
p = 5.3 x 10^-19
Proximity pairs /nucleus
J
Control
U2OS
C1 TIR1-
Cell number [x1000]
PI [r.U.]
BrdU-FITC [rU] [10^x]
Auxin
H
PLA: 8WG16-RNAPII/γH2AX
PLA: pS5-RNAPII/γH2AX
p = 1
p = 8.9 x 10^-1

SPT6 is essential for the recruitment of termination factors and prevents replication stress. Related to Figure 7. (A) Immunoblot of HA-RPB3-expressing and control U2OS$^{SPT6\text{-}AID\text{-}C1}$ cells. Cells were treated with auxin for 4 h and used for quantitative mass spectrometry. VINC, vinculin loading control. Membrane was cut just above the Vinculin bands. **(B)** Immunoblot of termination-associated proteins. U2OS$^{SPT6\text{-}AID\text{-}C1}$ cells expressing HA-RPB3 were treated with auxin for the indicated times. Histone H3 or GAPDH, loading control (separate membranes). **(C)** Growth curves of U2OS^{WT} and U2OS$^{SPT6\text{-}AID\text{-}C1}$ cells in presence or absence of auxin. Values are mean ± SD (n=3). Two-way ANOVA. **(D)** Annexin V-PI assay. Cells were treated or not with auxin (24 h), stained with annexin V, Pacific Blue conjugate and propidium iodide (PI), and counted by flow cytometry (50,000 sorted events). Early apoptosis, annexin+ PI; late apoptosis, annexin+ PI+. Values are mean ± SD, n=3. **(E)** Browser tracks showing γH2AX and histone-H3 ChIP-Rx experiments for *DNAJB5* gene in U2OS$^{SPT6\text{-}AID\text{-}C1}$ cells in presence or absence of auxin (24 h). **(F)** Box plot of γH2AX ChIP-Rx reads compared between auxin and control in the promoter and readthrough region, displayed across 7,562 expressed genes. **(G)** Heatmaps of histone-H3 ChIP-Rx experiments in U2OS$^{SPT6\text{-}AID\text{-}C1}$ cells in presence or absence of auxin (24 h), around PAS, averaged for 4.5 kb upstream and 12.5 kb downstream over 7,562 expressed genes, sorted by length. **(H)** Immunofluorescence images (left) of proximity ligation assays (PLAs) between unphosphorylated-RNAPII (top), and pS5-RNAPII (bottom) and γH2AX in U2OS$^{SPT6\text{-}AID\text{-}C1}$ cells treated with or without auxin. Quantification (right) of PLA foci per nucleus from the corresponding conditions. Unpaired one-sided Wilcoxon test for the likelihood of auxin being more than control is calculated. **(I)** Immunofluorescence images (left) of PLAs between S9.6 (RNA-DNA hybrid) and H2AX in U2OS$^{SPT6\text{-}AID\text{-}C1}$ cells treated or not with auxin (scale bar: 15 µm). Quantification (right) of PLA foci per nucleus. Unpaired two-sided Wilcoxon test. **(J)** Cell cycle distribution assay. Cells were treated or not with auxin (24 h), labeled with BrdU, stained with PI, and analyzed by flow cytometry. Shown are the amount of intercalating PI (top) and the correlation of BrdU to PI (bottom). Cells that are BrdU-positive in S-phase are marked in green, while those that are negative are red.
